# Nitrogen fixation efficiency enhancement using plasma jet: Impact of grounded electrode

**DOI:** 10.1371/journal.pone.0346219

**Published:** 2026-09-01

**Authors:** Yasin Arafat, Mojahidur Rahman, Atik Sheikh, M. R. Talukder

**Affiliations:** Department of Electrical and Electronic Engineering, Plasma Engineering Laboratory, University of Rajshahi, Rajshahi, Bangladesh; Donghua University, CHINA

## Abstract

Energy cost reduction for nitrogen fixation (NF) using non-thermal plasma remains a global challenge. Researchers are exploring to reach the theoretical energy cost limit of 0.2 MJ.mol^−1^ for NF. A fixed grounded electrode (FGE) plasma jet has designed, studied NO_x_ production efficiency and energy cost, and compared the results with the traditional floating water ground electrode (FWGE) configuration. The plasma was characterized by the optical emission spectroscopic (OES) technique. The experimental setup was optimized for gas composition and flow rate. The efficient gas composition ratio was identified as air:O_2_::5:3 with the flow rate of 3 L.min^−1^, and the temporal evolution of NO_x_ production and efficiency were analyzed for both electrode configurations. For FGE, the NO_x_ synthesis rate was 3.83 mmol.h^−1^ and the energy cost was 25.74 MJ.mol^−1^ for a 10 min treatment, and the synthesis rate was decreased, whereas the energy cost was increased with water treatment time. While for FWGE, the synthesis rate and energy cost were 1.71 mmol.h^−1^ and 71.5, MJ.mol^−1^, respectively, for the same treatment time. The synthesis rate was decreased with treatment time, whereas the energy cost was increased initially but decreased as the treatment time progresses. The promising result of this experiment is that the FGE configuration achieved a significantly lower energy cost and higher synthesis rate compared to FWGE. The proposed FGE plasma jet system might provide a way in plasma-assisted NF at low energy cost through the synergistic optimization of ground electrode, jet configuration, plasma-catalyst incorporation, gas flow dynamics, and treatment duration.

## 1. Introduction

Recent improvements in atmospheric pressure cold (APC) plasma have drawn curiosity among researchers because of its versatile applicability in a wide range of research fields [1]. APC plasmas are produced using a variety of high-voltage (HV) power sources and admixture of feed gases. Neutral gas molecules and electrons in the APC plasmas retain a sizable temperature differential, in contrast to the thermal plasmas, where the entire gas is in thermal equilibrium with the electron temperature (${\text{T}}_{\text{e}}$) [2]. The arc discharge jet operates under atmospheric pressure with relatively low discharge power and high gas flow rate, which promotes non-equilibrium characteristics of arc discharge plasma jet. One advantage of these conditions is that the heavy particles, e.g., molecules and ions, remain at low temperatures, which makes this method useful for treating the surface of heat-sensitive materials as well as effectively interacting with the surface and bulk water molecules for ${\text{NO}}_{\text{x}}({\text{NO}}_{\text{2}}^{-}+{\text{NO}}_{\text{3}}^{-})$ synthesis. As opposed to that, ${\text{T}}_{\text{e}}$ rises to such a point where it produces a broad range of species, including reactive nitrogen species (RNS) and reactive oxygen species (ROS), which are mainly responsible for seed surface modifications, ${\text{NO}}_{\text{x}}$ production in water, surface modification, plasma nitriding, and so on [3].

Nitrogen fertilizer is dominantly produced by well-established Haber-Bosch (H-B) technology [4]. H-B technology includes a number of processes, such as (i) ${\text{N}}_{\text{2}}$ extraction from air, (ii) production of hydrogen (${\text{H}}_{\text{2}}$) from fossil fuels using steam reforming techniques, and (iii) catalytic transformation of ${\text{N}}_{\text{2}}$ and ${\text{H}}_{\text{2}}$ via iron-based catalysts to produce ${\text{NH}}_{\text{3}}$ in the extreme temperatures of 400–650°C at 200–400 atm pressure [5]. The most alarming issues involved with the H-B processes are that they consume 1–2% of global energy, 3–5% of the world’s fossil fuel, and terribly release about 300 million tons of carbon dioxide (CO_2_) annually [4]. Therefore, in order to establish a sustainable solution to address these issues towards emissions-free industries and societies for renewable ${\text{NH}}_{\text{3}}$ or ${\text{NO}}_{\text{x}}$ synthesis, the plasma-driven, electrochemical, and photochemical synthesis of ${\text{NH}}_{\text{3}}$ or ${\text{NO}}_{\text{x}}$ might be the alternatives [5]. APC plasma is one of the promising technologies that would be a substitute for ${\text{NH}}_{\text{3}}$ or ${\text{NO}}_{\text{x}}$ synthesis for agricultural produce through ecologically friendly technology. The mechanism through which ${\text{N}}_{\text{2}}$ is turned into ${\text{NH}}_{\text{3}}$ or ${\text{NO}}_{\text{x}}$ is called nitrogen fixation (NF). The dominant pathway for nitric oxide (NO) formation is the Zeldovich mechanism. In this process, molecular nitrogen (N_2_) contributes either through vibrational excitation or electronic excitation [6]. NF in water by plasma technology is termed as plasma water-based nitrogen fixation (PWBNF). Plasma-liquid interactions produce aquatic reactive species, such as nitrogen oxides (NO) and hydroxyl radicals (OH•), which are capable of efficiently oxidizing ${\text{N}}_{\text{2}}$ at the plasma phase, and later the oxidized species can be transported to the bulk water during plasma-liquid interactions. This plasma-activated water (PAW), contains plasma-generated species (${\text{NO}}_{\text{2}}^{-}$, ${\text{NO}}_{\text{3}}^{-}$ H_2_O_2_, ${\text{NH}}_{\text{4}}^{+}$), can be absorbed by plants as an immediate source of nutrients [7]. Despite the fact that PWBNF presents a viable substitution for the well-established H-B process, but its energy requirement is still a significant constraint. In order to synthesize ${\text{NH}}_{\text{3}}$, the researchers have been investigating the direct interaction of plasma jets in water since the last decade, and this process requires a remarkable amount of energy ~1016.9 MJ.mol^−1^ with the production rate of 1.062 mmol.h^−1^ [7]. However, the researchers are continuing their research on the reduction of energy cost for NF to establish an eco-friendly, and cost-effective way. Bian et. al [8] designed a pulsed discharge plasma source that fixes nitrogen with 403 MJ.mol^−1^ of energy cost. Peng et. al. [9] designed a bubble discharge plasma reactor that fixes nitrogen at the rate of 0.672 mmol.h^−1^ with 696.4 MJ.mol^−1^ of energy cost. Hawtof et. al. [10] reported an NF rate of 0.024 mmol.h^−1^ using a plasma-electrolytic system at 138.9 MJ.mol^−1^, and Toth et. al. [11] achieved a higher rate of 0.06 mmol.h^−1^ with a plasma-water droplet reactor at an energy cost of 1900 MJ.mol^−1^. Fujera et.al. [12] achieved higher rate using streamer spark discharge of 2.9 mmol.h^−1^ with energy cost of 62.5 MJ.mol^−1^. Gorbanev et. al. [13] employed a plasma jet and produced 0.024 mmol.h^−1^ of ${\text{NO}}_{\text{x}}$ with a significantly improved efficiency of 15 MJ.mol^−1^, while Dinh et. al. [14] demonstrated the most energy-efficient design, an arc-DBD hybrid system that required only 8 MJ.mol^−1^ for producing ${\text{NO}}_{\text{x}}$.

The production rate of ${\text{NO}}_{\text{x}}$ and production cost mainly depend on the plasma properties. The four fundamental plasma properties are rotational temperature (${\text{T}}_{\text{r}}$), vibrational temperature (${\text{T}}_{\text{v}}$), electron excitation temperature (${\text{T}}_{\text{ex}}$), and electron density (${\text{n}}_{\text{e}}$). The plasma parameters are governed by many factors, such as electrode gap, gas flow rate, gas mixture, pressure, modifications in plasma-liquid interface, utilization of catalyst, UV, presence of magnetic field, amplitude and frequency of power supply, and so on [15–18]. ${\text{T}}_{\text{r}}$, frequently regarded as gas temperature (${\text{T}}_{\text{g}}$) in nonequilibrium plasma, typically provides details regarding the translational energy as well as demonstrates the ground-level rotational population of the plasma [19]. Further, the vibrational excitation often regulates the interactions involving electrons and molecular species [20,21]. In plasma, the energetic electrons excite ${\text{N}}_{\text{2}}$ molecules more effectively to their vibrationally excited states through several collisional pathways in the gas phase or disintegrate the nitrogen bond (N≡N).

In our previous works [22–25], water was employed as a floating ground electrode. Replacement of floating water ground electrode (FWGE) with fixed ground electrode (FGE) configuration may offer several advantages. These include the facilitation of a more stable and continuous discharge, improved control over plasma conditions, high species densities, and minimized susceptibility to discharge interruption caused by gas flow dynamics. However, the key distinction between the proposed experimental setup and the arc jet discharge with FWGE configuration [24,25] lies in the design of the ground electrode. In a water-grounded bubble discharge system, water acts as a floating ground. As the gas bubbles are introduced into the water through the capillary tubes, they create irregular and dynamic electrode spacing between the power electrode and the grounded water, often creating discontinuous discharge due to longer electrode spacing and thereby requiring increased discharge voltage. In contrast, the proposed setup employed an FGE for constant electrode spacing, which ensures a fixed electrode gap that enables the generation of continuous and sustainable discharges, potentially enhancing the overall discharge performance. In this investigation, we have focused on the efficiencies of $\text{N}{\text{O}}_{\text{x}}$ production rate in water and their energy cost in an arc discharge jet (ADJ) plasma source with FGE and FWGE, and accordingly, an ADJ plasma source has been designed. In order to reveal the physicochemical mechanisms for NF in water, ADJ gas phase plasma properties by optical and electrical diagnostics and liquid phase by spectrophotometric test kit methods are employed for characterization. The ADJ plasma configuration was deployed for water treatment applications, with particular emphasis on evaluating its influence on the $\text{N}{\text{O}}_{\text{x}}$ synthesis rate and corresponding energy consumption.

## 2. Materials and methods

### 2.1. Experimental details

**Fig 1:**
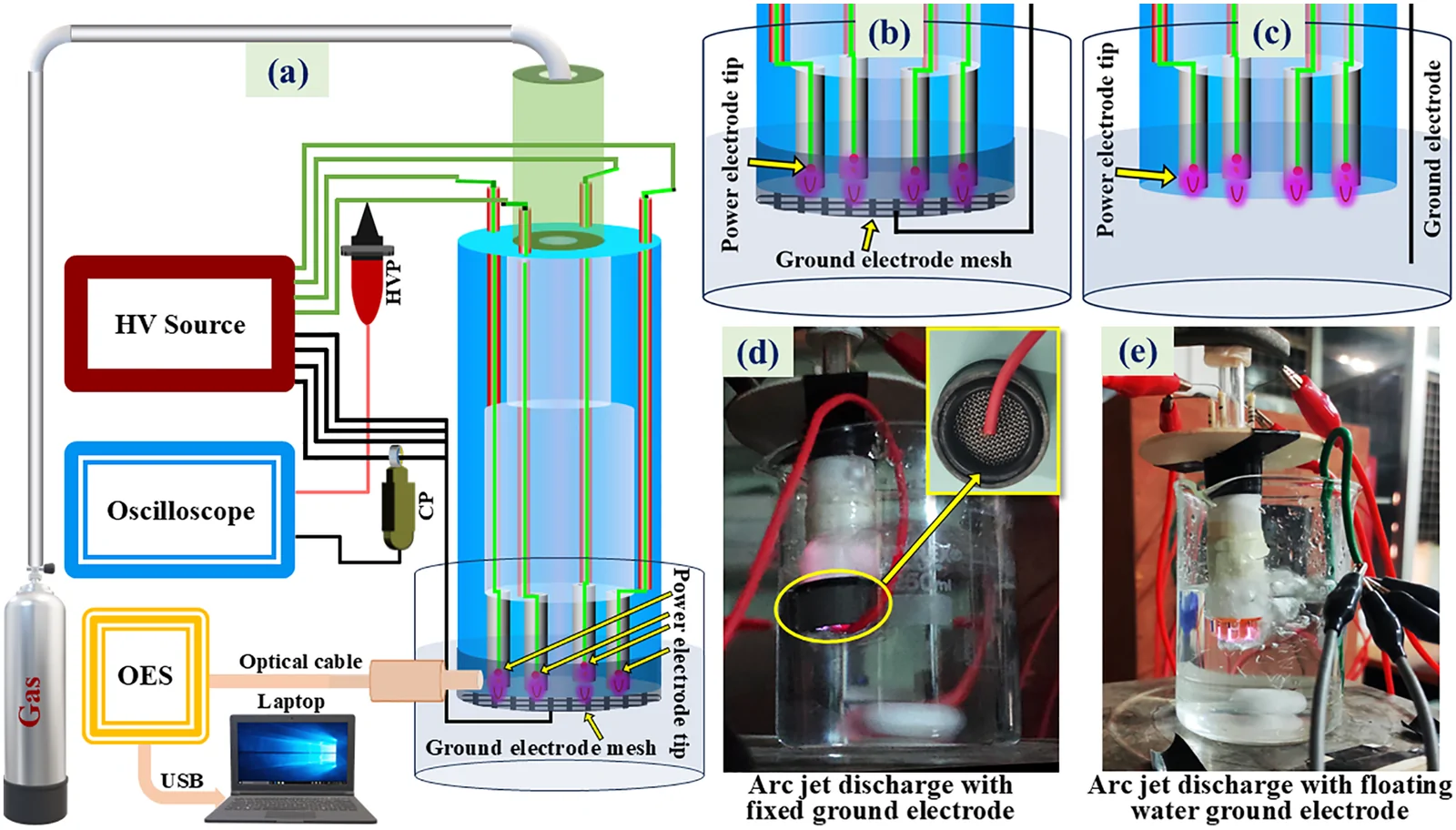
Schematic of the (a) experimental setup and (b) enlarged view of fixed ground electrode (FGE) configuration and (c) enlarged view of floating water ground electrode (FWGE) configuration. Photograph of the (d) fixed ground electrode setup and (e) floating water ground electrode setup during water treatment.

An ADJ plasma generator was designed aimed at increasing NO_x_ synthesis in water. A schematic of the whole arrangement, including water treatment, current voltage measurement, and optical measurement, is depicted in Fig 1(a). The ADJ plasma generator was constructed with a Teflon rod (length 60 mm and diameter 19 mm). A cylindrical channel (diameter 9 mm and depth 30 mm) was drilled along the central axis of the Teflon rod. A Pyrex glass tube (outer diameter 9 mm and length 95 mm) was inserted through this channel to deliver gas admixture into the four plasma jets. Additionally, a cavity (diameter 14 mm and depth 20 mm) was made at the end of the cylindrical channel to serve as a gas reservoir. Four Pyrex glass capillary tubes (inner diameter 1 mm, outer diameter 6.4 mm, and length 17 mm) were inserted, keeping equal distance from each other at the periphery of the Teflon rod, into 10 mm depth at the end of the Teflon rod, and four bores (from top to the capillary bores) were prepared axially for insertion of power electrodes along with a gas injection facility. Four power electrodes, made of nichrome wire (diameter 0.45 mm and length 56 mm), were inserted through the four Teflon holes as well as the capillary tubes. The ground electrode is a fixed, externally connected, common ground electrode fabricated as a stainless-steel (SS) mesh enclosed within a cylindrical Polyvinyl Chloride (PVC) holder. The PVC holder is aligned longitudinally such that the SS mesh makes direct physical contact with the bottom exterior surface of the Pyrex glass capillary tube, and the spacing between the power electrodes and ground electrode mesh was 3.5 mm.

The four power lines were then separately connected to the four individual identical DC high-voltage (HV) sources (10 kV) through four ballast resistors with a resistance of 10 kΩ, whereas the four ground lines were collectively connected to the fixed mesh ground electrode (for FGE configuration as shown in Fig 1(d)) and submerged floating ground electrode (for FWGE configuration as shown in Fig 1(e)). The discharge voltage was measured using an HV probe (HVP) by connecting it to the power line, and the discharge current was measured using a clamp-type current probe (CP) by connecting it to the ground line. An oscilloscope was connected to the HVP and CP to measure and record the discharge voltage and current data and waveforms. The optical data emitted from the plasma discharge was recorded using two optical emission spectrometers (OES) (Ocean Optics and Avantes). Figs 1(b) and (c) present the schematic of the enlarged fixed ground electrode configurations and the floating water ground electrode, respectively, whereas Figs 1(d) and 1(e) show the photograph of the ADJ experimental setup during water treatment for FGE and FWGE configurations, respectively. Air, nitrogen (N_2_), oxygen (O_2_), and argon (Ar) gas cylinders were connected to the inlet of the Pyrex glass tube through a gas flow controller valve. To synthesize NO_x_ in water, a 250 mL Pyrex glass beaker was filled with 150 mL distilled water. Then the end of the Teflon tube, where the plasma jet was generated, was immersed in the water to be treated. Initially, experiments were conducted with the FGE configuration by systematically varying the gas composition ratio (${\text{N}}_{\text{2}}$, air, air:${\text{O}}_{\text{2}}$:: 5:3, air: ${\text{O}}_{\text{2}}$::1:3, and ${\text{O}}_{\text{2}}$) with a flow rate of 4 L.min^−1^ to identify the mixture that produces the highest ${\text{NO}}_{\text{x}}$ while keeping other parameters constant. Once the optimal composition was identified, it was fixed for the subsequent step, where gas flow was varied across a range of 2 L.min^−1^ to 6 L.min^−1^. The experiments compared the temporal evolution of the FGE and FWGE setups under optimized gas composition and flow rate conditions that maximize ${\text{NO}}_{\text{x}}$ yields. Throughout the experimentation a fraction of Ar gas (≈1%) was used in order to study the physical properties of plasma.

### 2.2. Plasma diagnostics

OES is a well-known technique that is frequently employed to assess atmospheric pressure plasma characteristics as well as detect the species produced in plasma. Two OES devices were utilized in this experiment. An OES ‘Ocean Optics (USB2000+XR1)’ was utilized to record the emitted spectra from the ADJ for the purpose of species detection. Besides, a high-resolution OES device, ‘AvaSpec-2018 (ULS3648-2-USB2, Avantes)’ was utilized for the determination of the plasma parameters, such as T_r_, T_v_, T_ex_, and n_e_. The specifications of the two above mentioned spectrometers are presented in Table 1. To record the OES data directly from the multi-electrode arc discharge zone, a 3D-movement stand was employed to set the optical cable. The stand can move left-to-right, up-to-down, and front-to-back with the help of the Vernier wheel so that the cable can be placed as per requirement.

**Table 1:** Specifications of spectrometers and power measuring instruments.

Model	Specification
Ocean Optics (USB2000 + XR1)	Wavelength range: 200–1100 nm, resolution: 1.7 nm, slit length: 25 μm, and grating: 800 lines/mm
AvaSpec-2018 (ULS3648–2-USB2, Avantes)	Wavelength range: 300–500 nm, resolution: 0.07 nm, slit length: 10 μm, and grating: 2400 lines/mm
Digital Oscilloscope	RIGOL DS1104 z plus, 4 Channel, Bandwidth: DC ~ 100 MHz, Real time sampling rate: 1 GSa/s, Time base accuracy: 25 ppm, DC gain accuracy: ± 4% (<10 mV/div), ± 3% (≥10 mV/div)
High Voltage Probe	Gould Advance, UK, Max voltage: 40 kV
Current Probe	CP6220 (ELDITEST), 100 Amp AC/DC, Bandwidth: DC ~ 300 kHz, Rise and fall time 1.2 µs, DC measurement accuracy ±3% (100 mV/A) and ±4% (10 mV/A)

**Fig 2:**
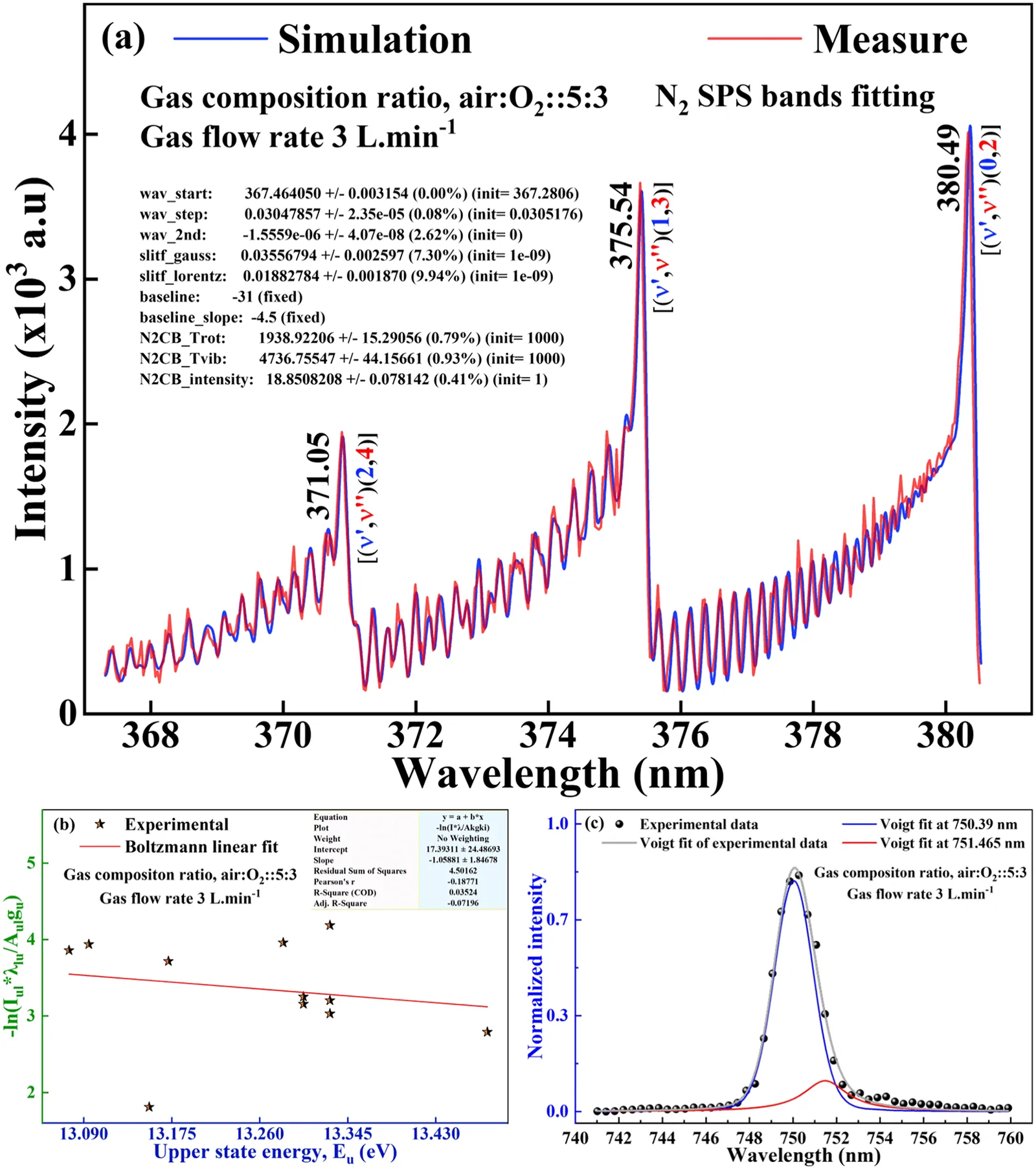
(a) N_2_ (C-B) band fitting using massiveOES software for estimating rotational (T_r_) and vibrational (${𝐓}_{𝐯}^{𝐂}$) temperature, (b) Boltzmann linear fit for estimating the T_ex_, and (c) Voigt fit of Ar I (750.39 nm) line for determining electron density n_e_ for fixed ground electrode configuration with gas composition ratio of air:O_2_::5:3 and gas flow rate of 3 L.min^−1^.

There are many radiative transitions that have non-overlapping diatomic molecular bands, which are usually employed to determine T_r_ and T_v_ [21]. These bands include ${\text{N}}_{\text{2}}({\text{C}}^{\text{3}}\Pi_{\text{g}}\to {\text{B}}^{\text{3}}\Pi_{\text{u}})$ [26,27], ${\text{N}}_{\text{2}}^{+}({\text{B}}^{\text{2}}\Sigma_{\text{g}}\to {\text{X}}^{\text{2}}\Sigma_{\text{u}})$ [19,27,28], and $\text{OH}({\text{A}}^{\text{2}}\Sigma^{+}\to {\text{X}}^{\text{2}}\Pi_{\text{i}})$ [29,30]. The rotational and vibrational energy distribution, regarding plasma volume and excited species population, are the primary determinants of plasma species’ optical emission intensity. In this investigation, T_r_ and vibrational temperature of the N_2_(C, v) excited state (${\text{T}}_{\text{v}}^{\text{C}}$) were calculated using the band fitting of ${\text{N}}_{\text{2}}({\text{C}}^{\text{3}}\Pi_{\text{g}}\to {\text{B}}^{\text{3}}\Pi_{\text{u}})$ by massiveOES software [31] within 367‒381 nm of the wavelength range, since this band provided the highest emission intensity. Importantly, ${\text{T}}_{\text{v}}^{\text{C}}$ is far different from the ground state vibrational temperature, T_v_. However, a non-linear correlation exists between them, as ${\text{T}}_{\text{v}}^{\text{C}}$ is an increasing function of T_v_[33]. The excitation process, collisional quenching, and vibrational relaxation in the manifold of the excited state are the mechanisms by which this correlation persists [32]. An automatic fitting approach is a good option for improving the reliability of assessing temperature, as ${\text{T}}_{\text{r}}$ and ${\text{T}}_{\text{v}}^{\text{C}}$ were determined, while ${\text{T}}_{\text{ex}}$ was obtained by performing fitting of experimental data [33]. Fig 2(a) shows the Lorentzian and Gaussian fitting of the N_2_(C-B) band using massiveOES software for the ADJ with FGE configuration at a gas flow rate of 3 L.min^−1^ with a composition of air:O_2_::5:3.

Without taking into account the inelastic collisions between heavy particles, T_ex_ may be roughly represented as T_e_ in the context of extremely dense non-equilibrium plasma [34,35]. This is consistent with the hypothesis that free electron impact serves as the primary source of molecular and atomic excitations, which should adhere to the Boltzmann distribution function of energy for a specific excitation. The Boltzmann plot is a useful technique for determining T_ex_ that uses the relative intensity of more than two spectral lines using equation (1) [16]. The values of transition parameters necessary for equation (1) were taken from the NIST database [36]. In this investigation, T_ex_ was determined using the Ar I (at 696.54, 706.72, 738.39, 750.39, 763.51, 772.42, 794.82, 801.47, 811.53, 826.45, and 866.79 nm) [37,38] lines with the concern that the upper (${\text{E}}_{\text{u}}$) and lower (${\text{E}}_{\text{l}}$) energy levels have a disparity in energy substantially greater in relation to electron energy $\left({\text{E}}_{\text{u}}-{\text{E}}_{\text{l}}\gg {\text{k}}_{\text{B}}{\text{T}}_{\text{e}}\right)$. The Boltzmann plot for standard Ar emission lines, generated using Origin 2024 software under ADJ with FGE operation at a gas flow rate of 3 L.min^−1^ (air:O_2_::5:3), is illustrated in Fig 2(b).


$$\begin{array}{c} n\left(\frac{I_{ul}\lambda_{lu}}{A_{ul}g_{u}}\right)=-\frac{E_{u}}{k_{B}T_{ex}}+C. \end{array}$$ (1)

Notation details: $E_{u}$ = upper-level energy, $k_{B}$ = Boltzmann constant, C = constant related to Plank’s constant, $I_{ul}$ = emitted light intensity, $\lambda_{lu}$ = transition wavelength, $A_{ul}$ = transition probability, and $g_{u}$ = statistical weight.

On the other hand, n_e_ was determined using the broadening profile of the spectral line. There may be several phenomena involved in the plasma that are responsible for the line broadening of spectral emission. These line broadenings include ${\Delta \lambda }_{\text{S}}$ = Stark broadening, ${\Delta \lambda }_{\text{W}}$ = van der Waals broadening, ${\Delta \lambda }_{\text{D}}$ = Doppler broadening, ${\Delta \lambda }_{\text{I}}$ = instrumental broadening, ${\Delta \lambda }_{\text{N}}$ = natural broadening, and ${\Delta \lambda }_{\text{R}}$ = resonance broadening [39]. Among them, ${\Delta \lambda }_{\text{S}}$ and ${\Delta \lambda }_{\text{W}}$ obey the Lorentzian profile of distribution, whereas ${\Delta \lambda }_{\text{D}}$ and ${\Delta \lambda }_{\text{I}}$ obey the Gaussian profile of distribution. ${\Delta \lambda }_{\text{N}}$ and ${\Delta \lambda }_{\text{R}}$ are trivial for the conditions of this investigation [25,39]. The Voigt fitting convolves the two distribution functions. The Lorentzian distribution, which is expressed as ${\Delta \lambda }_{\text{L}}={\Delta \lambda }_{\text{S}}+{\Delta \lambda }_{\text{W}}+{\Delta \lambda }_{\text{N}}+{\Delta \lambda }_{\text{R}}$, and the Gaussian distribution, which is expressed as ${\Delta \lambda }_{\text{G}}=\sqrt{{{\Delta \lambda }_{\text{D}}}^{\text{2}}+{{\Delta \lambda }_{\text{I}}}^{\text{2}}}$. All the broadening mechanisms are included in the FWHM value of the Voigt function, and ${\Delta \lambda }_{\text{W}}$ is approximately accounted for as 30–35% of the FWHM value given by the Lorentzian profile [40]. In this investigation, the standard overlapped Ar lines of 750.39 nm and 751.465 nm was used for double component Voigt fitting and FWHM value of the Stark broadening at 750.39 nm was used for n_e_ calculation using the simplified equation (2) [41]. The function of Voigt fitting was carried out by Origin software (2021) [42,43]. Fig 2(c) presents the double component Voigt profile fitting of the standard Ar emission lines, performed using Origin 2024 software under ADJ with FGE operation at a gas flow rate of 3 L.min^−1^ with a gas composition of air:O_2_::5:3.


$$\begin{array}{c} n_{e}=\frac{{\Delta \lambda }_{\text{1}/\text{2}}}{\text{2}\omega }\times {\text{1}\text{0}}^{\text{1}\text{6}}\;{\text{cm}}^{-\text{3}}. \end{array}$$ (2)

Notation details: ${\Delta \lambda }_{\text{1}/\text{2}}$ = FWHM value of ${\Delta \lambda }_{S}$ in nm, ω = electron impact width parameter value taken from Griem [44].

### 2.3. Liquid phase analysis

In this investigation, the pH and the concentrations of other species, such as NO_x_ ($={\text{NO}}_{\text{2}}^{-}+{\text{NO}}_{\text{3}}^{-})$, H_2_O_2_, and O_3_, in PAW were measured. The species concentrations were measured by the optical absorbance method, and pH was measured by a pH tester. In the optical absorbance method, a required volume (according to the manufacturer’s suggestion) of PAW was taken, then the powder from the specific test kit (for specific species measurement, like ${\text{NO}}_{\text{2}}^{-}$, or ${\text{NO}}_{\text{3}}^{-}$, or H_2_O_2_, or O_3_) was mixed gently with the sample to establish the suggested color. After establishing the required color of the sample, the optical absorbance was taken by a UV-VIS spectrophotometer at their highest absorption peak. The absorption peaks were found at 390.00, 507.00, and 351.50 nm for ${\text{NO}}_{\text{3}}^{-}$, ${\text{NO}}_{\text{2}}^{-}$, and H_2_O_2_, respectively. O_3_ was quantified using a test kit. The concentration of each species was determined from the corresponding absorbance value using the predetermined absorbance calibration curves. It should be noted here that all the measurements were completed within 10 min after the treatment. The model and specifications of the measuring equipment are presented in Table 2.

**Table 2:** Model and specifications of the characterization equipment.

Equipment	Specification & Model
pH tester	Range: 0–14 pH (0–50°C), resolution: 0.1 pH/0.1°C, and accuracy: ± 0.1 pH/ ± 0.5°C (pHep, HI98107)
UV-VIS spectrophotometer	UV-1900i, Shimadzu Corporation, Japan; wavelength range: 180–1100 nm, resolution: 1 nm
Test kit (for ${\text{NO}}_{\text{3}}^{-}$)	HI3874–0, Hanna Instrument, USA
Test kit (for ${\text{NO}}_{\text{2}}^{-}$)	HI3873–0, Hanna Instrument, USA
Test kit (for ${\text{H}}_{\text{2}}{\text{O}}_{\text{2}}$)	HI3844, Hanna Instrument, USA
Test kit (for ${\text{O}}_{\text{3}}$)	HI38054, Hanna Instrument, USA

### 2.4. Measurement of dissipated power and energy cost

In this study, the electrical energy cost (E_NOx_) for NO_x_ production was calculated from the effective electrical discharge power (P_d_) and total moles production of NO_x_. The effective discharge power was calculated over a period (T) of the discharge voltage and discharge current measured by an oscilloscope. The value of P_d_ was calculated by summing the product of the discharge voltage and current over a period by equation (3). Then the value of E_NOx_ was calculated from P_d_, treatment time (t), and total mol production of ${\text{NO}}_{\text{3}}^{-}$ and ${\text{NO}}_{\text{2}}^{-}$ with the help of equation (4) [7,25].


$$\begin{array}{c} P_{d}=\sqrt{\frac{\text{1}}{N}\sum_{n=\text{1}}^{N}v(n).i(n),} \end{array}$$ (3)

$$\begin{array}{c} E_{{NO}_{x}}=\left(\frac{P_{d}\times t}{C_{{NO}_{x}}}\right)\times {\text{1}\text{0}}^{-\text{6}}\;{\text{MJ}.\text{mol}}^{-\text{1}}. \end{array}$$ (4)

Notation details: *N* = Number of data point, v(n) = discharge voltage, i(n) = discharge current, and $C_{{NO}_{x}}$ = total number of moles of ${\text{NO}}_{\text{3}}^{-}$ and ${\text{NO}}_{\text{2}}^{-}$. The specifications of instruments for the measurement of electrical power are presented in Table 1. The electrical power determined using the instruments include error in the range of 5–10%.

## 3. Results and discussion

### 3.1. Gas phase plasma properties

**Fig 3:**
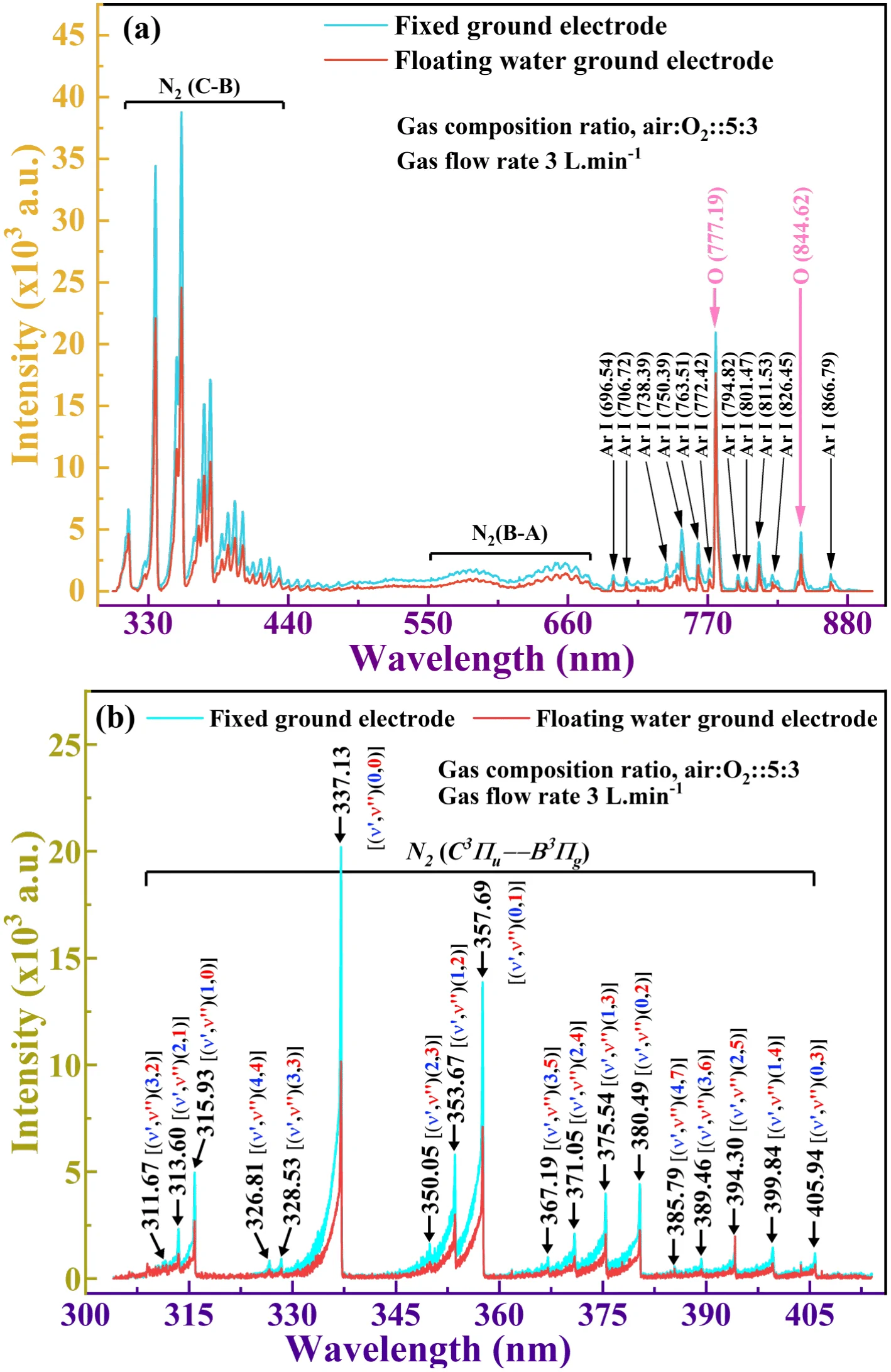
(a) OES spectra analysis of AJD plasma, species production along with the difference between the floating water ground electrode and the fixed ground electrode in the wavelength range 301–900 nm. (b) OES spectra of ${\text{N}}_{\text{2}}(\text{C}-\text{B})$ transitions in wavelength 304–414 nm of floating water ground electrode and fixed ground electrode configuration.

As mentioned earlier, the plasma diagnostics were performed using OES, as the OES spectra exhibit the dominant emitting plasma species’ spatial distribution, and the spectra of the species are time averaged [16]. Fig 3(a) exhibits the emitted spectra from the single jet of the ADJ source when the gas flow rate was 3 L.min^−1^ (air:O_2_::5:3). O_2_ was admixed with air to increase the oxidation of nitrogen [45]. According to Fig 3(a), the dominant plasma species in the gaseous phase detected in ADJ plasma was nitrogen second positive system (SPS), ${\text{N}}_{\text{2}}({\text{C}}^{\text{3}}\Pi \to {\text{B}}^{\text{3}}\Pi )$. Also, O radicals at 775.86 nm and 843.3 nm, and Ar lines were detected. According to Fig 3(b), the densely populated species of ${\text{N}}_{\text{2}}({\text{C}}^{\text{3}}\Pi \to {\text{B}}^{\text{3}}\Pi )$ band were at 315, 337, 353, 357, 367, 370, 375, and 380 nm within the wavelength range from 300 nm to 420 nm. All the species were produced in this ADJ plasma through several collisional mechanisms. The SPS (N_2_(C-B)) produced in this air/O_2_ ADJ plasma are due to the direct electron impact and dissociation, stepwise excitation, and ionization reactions via metastable nitrogen species N_2_(A) as represented by R1-R5 [16],


$$\begin{array}{c} {\text{N}}_{\text{2}}(\text{X})+\text{e}\overset{\alpha_{\text{1}}=\text{3}.\text{4}\times {\text{10}}^{-\text{10}}\text{c}{\text{m}}^{\text{3}}.{\text{s}}^{-\text{1}}\;}{\to }\;{\text{N}}_{\text{2}}(\text{C})+\text{e}, \end{array}$$ (R1)

$$\begin{array}{c} {\text{N}}_{\text{2}}(\text{X})+\text{e}\overset{\alpha_{\text{2}}=\text{1}.\text{0}\times {\text{10}}^{-\text{9}}\text{c}{\text{m}}^{\text{3}}.{\text{s}}^{-\text{1}}\;}{\to }{\text{N}}_{\text{2}}(\text{A})+\text{e}, \end{array}$$ (R2)

$$\begin{array}{c} {\text{N}}_{\text{2}}(\text{A})+\text{e}\overset{\alpha_{\text{3}}=\text{2}.\text{4}\times {\text{10}}^{-\text{9}}\text{c}{\text{m}}^{\text{3}}.{\text{s}}^{-\text{1}}\;}{\to }{\text{N}}_{\text{2}}(\text{C})+\text{e}, \end{array}$$ (R3)

$$\begin{array}{c} {\text{N}}_{\text{2}}(\text{A})+{\text{N}}_{\text{2}}(\text{A})\overset{\alpha_{\text{4}}=\text{1}.\text{5}\times {\text{10}}^{-\text{9}}\text{c}{\text{m}}^{\text{3}}.{\text{s}}^{-\text{1}}\;}{\to }{\text{N}}_{\text{2}}(\text{C})+{\text{N}}_{\text{2}}(\text{X}), \end{array}$$ (R4)

$$\begin{array}{c} {\text{N}}_{\text{2}}(\text{X},\text{A})+\text{e}\overset{\alpha_{\text{5}}=\text{2}.\text{4}\times {\text{10}}^{-\text{12}}\text{c}{\text{m}}^{\text{3}}.{\text{s}}^{-\text{1}}\;}{\to }{\text{N}}_{\text{2}}^{+}(\text{B})+\text{2e}, \end{array}$$ (R5)

The O radicals are produced most probably due to dissociation and dissociative ionization by electrons, as presented by R6-R8 [16],


$$\begin{array}{c} {\text{O}}_{\text{2}}(\text{X})+\text{e}\overset{\alpha_{\text{6}}=\text{3}.\text{2}\times {\text{10}}^{-\text{11}}\text{c}{\text{m}}^{\text{3}}.{\text{s}}^{-\text{1}}\;}{\to }\text{O}\left({\text{3}}_{\text{P}}\right)+\text{O}\left({\text{1}}_{\text{D}}\right)+\text{e}, \end{array}$$ (R6)

$$\begin{array}{c} {\text{O}}_{\text{2}}(\text{X})+\text{e}\overset{\alpha_{\text{7}}=\text{1}.\text{9}\times {\text{10}}^{-\text{12}}\text{c}{\text{m}}^{\text{3}}.{\text{s}}^{-\text{1}}\;}{\to }{\text{O}}^{-}+\text{O}, \end{array}$$ (R7)

$$\begin{array}{c} {\text{O}}_{\text{2}}(\text{X})+\text{e}\overset{\alpha_{\text{8}}=\text{4}.\text{6}\times {\text{10}}^{-\text{16}}\text{c}{\text{m}}^{\text{3}}.{\text{s}}^{-\text{1}}\;}{\to }{\text{O}}^{+}+\text{O}+\text{2e}. \end{array}$$ (R8)

The metastable Ar^m^ atoms may be produced by direct electron impact with reaction R9 [29], which generates $\text{OH}({\text{A}}^{\text{2}}\Sigma^{+}\to {\text{X}}^{\text{2}}\Pi )$ radicals through dissociation of H_2_O vapor by R10 [29]. Also, OH radicals may be generated by direct electron impact dissociation of H_2_O vapor by R11 [29],


$$\begin{array}{c} \text{Ar}+\text{e}\overset{\alpha_{\text{9}}=\text{6}.\text{63}\times {\text{10}}^{-\text{7}}\text{c}{\text{m}}^{\text{3}}.{\text{s}}^{-\text{1}}\;}{\to }{\text{Ar}}^{\text{m}}+\text{e}, \end{array}$$ (R9)

$$\begin{array}{c} {\text{H}}_{\text{2}}\text{O}+{\text{Ar}}^{\text{m}}\overset{\alpha_{\text{10}}=\text{7}.\text{8}\times {\text{10}}^{-\text{10}}{\text{cm}}^{\text{3}}.{\text{s}}^{-\text{1}}\;}{\to }\text{OH}(\text{A})+\text{H}+\text{e}, \end{array}$$ (R10)

$$\begin{array}{c} {\text{H}}_{\text{2}}\text{O}+\text{e}\overset{\alpha_{\text{11}}=\text{1}.\text{9}\times {\text{10}}^{-\text{10}}{\text{cm}}^{\text{3}}.{\text{s}}^{-\text{1}}\;}{\to }\text{OH}(\text{A})+\text{H}+\text{e}. \end{array}$$ (R11)

The gas phase ozone (O_3_) may be produced by the following reaction (R12).


$$\begin{array}{c} {\text{O}}_{\text{2}}+\text{O}\overset{\alpha_{\text{14}}=\text{1}.\text{69}\times {\text{10}}^{-\text{12}}{\text{cm}}^{\text{3}}.{\text{s}}^{-\text{1}}\;}{\to }{\text{O}}_{\text{3}}. \end{array}$$ (R12)

It should be noted here that $\alpha_{\text{1}}-\alpha_{\text{14}}$ are the rate coefficients of the corresponding reactions.

Furthermore, Figs 3(a) and 3(b) illustrate the effect of FGE and FWGE configurations on species population densities. As shown in Figs 3(a) and 3(b), the relative intensities of the ${\text{N}}_{\text{2}}(\text{C}-\text{B})$ and O radicals are approximately two times higher in the FGE configuration. This indicates enhanced species population densities, more uniform plasma distribution, and more stable and continuous discharge in the FGE configuration compared to the FWGE.

**Fig 4:**
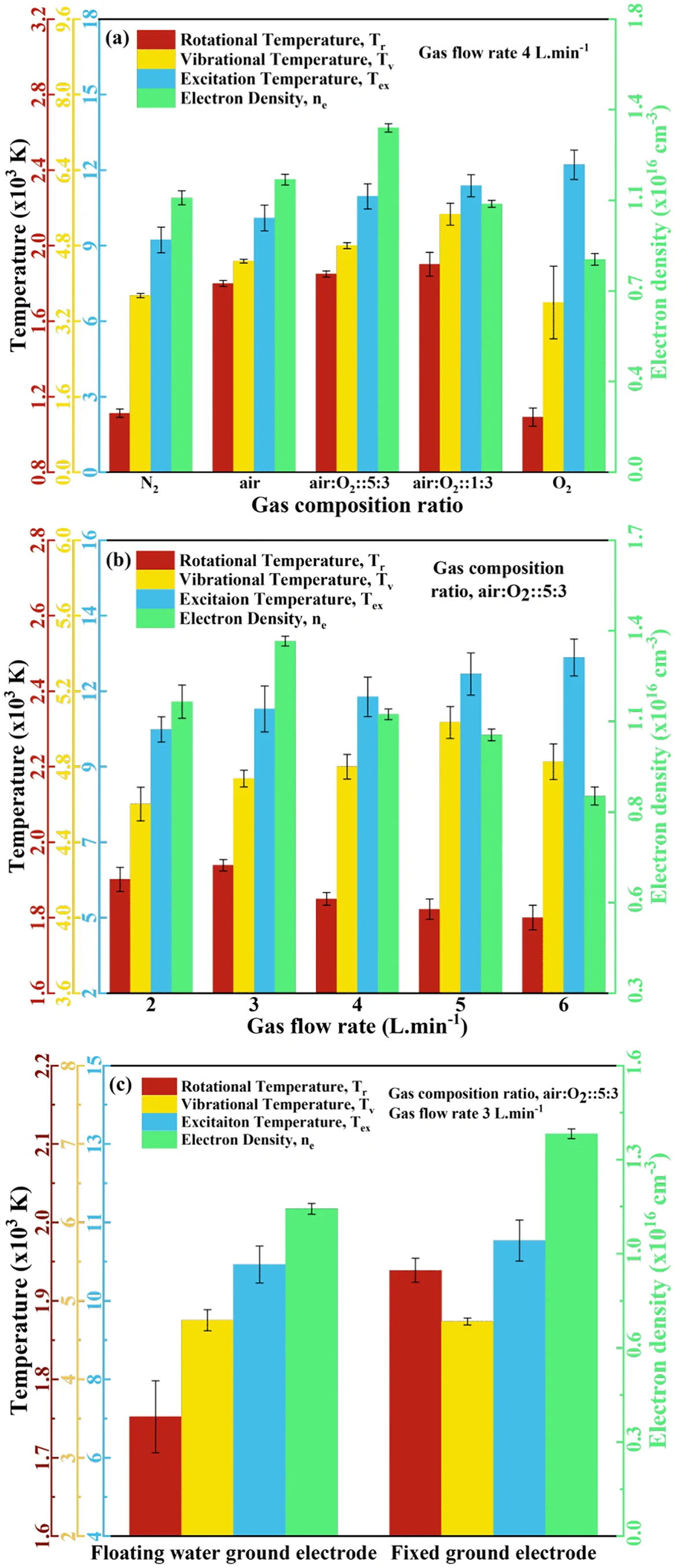
Influence of (a) gas composition ratio and (b) gas flow rate on plasma parameters (T_r_, ${𝐓}_{𝐯}^{𝐂}$, T_ex_, and n_e_) using fixed ground electrode configuration. (c) Comparison of plasma parameters for fixed ground electrode and floating water ground electrode configuration.

Fig 4(a) shows the variation of plasma parameters, namely T_r_, ${\text{T}}_{\text{v}}^{\text{C}}$, T_ex_, and n_e_, as a function of gas composition. An increase in O_2_ content leads to a corresponding rise in T_r_, ${\text{T}}_{\text{v}}^{\text{C}}$, and T_ex_. n_e_ initially increases; however, once the air:O_2_ ratio exceeds 5:3 (i.e., N_2_:O_2_::1:1, considering 78% N_2_ and 21% O_2_ in air), n_e_ begins to decrease. This behavior can be attributed to the increased gas phase electrical resistance caused by higher O_2_ content in the gas mixture, resulting in enhanced Joule heating [46]. In addition, the electronegative nature of O_2_ increases the sustaining voltage and electric field strength, thereby raising the electron kinetic energy and contributing to higher T_r_ and ${\text{T}}_{\text{v}}^{\text{C}}$. Furthermore, electrons quenched by excited O radicals that reduce the electron density and hence lessen the effective electron-neutral collision frequency [34,35].

Fig 4(b) illustrates the influence of gas flow rate (2.6 L.min^−1^) on key plasma parameters. With increasing gas flow rate, both ${\text{T}}_{\text{v}}^{\text{C}}$ and T_ex_ exhibit a slight increasing trend. In contrast, T_r_ and n_e_ initially increase up to a flow rate of 3 L.min^−1^ and subsequently decrease at higher flow rates.

The initial enhancement of plasma parameters can be attributed to the increased molecular density within the plasma jet at higher gas flow rates, which results in more frequent electron-neutral collisions. These collisions promote efficient energy transfer to gas molecules through the electron-impact excitation process [47]. Beyond a flow rate of 3 L.min^−1^, the gas flow transition occurs from laminar to turbulent regimes, leading to a reduced energy transfer time, increased breakdown voltage, and a higher Reynolds number (R_e_), which is defined as


$$\begin{array}{c} R_{e}=\frac{n_{g}v_{g}I_{c}}{\mu_{d}}. \end{array}$$ (5)

Where $n_{g},v_{g},I_{c}$ and $\mu_{d}$ denote the gas density, gas velocity, characteristic length, and dynamic viscosity, respectively [34]. A higher Reynolds number indicates an increased accumulation of gas molecules in the discharge region, which restricts electrons from acquiring sufficient energy from the applied electric field. Consequently, energy transfer to gas molecules through momentum-transfer collisions is reduced [48]. This phenomenon accounts for the observed decrease in T_r_ and n_e_ beyond a gas flow rate of 3 L.min^−1^.

Furthermore, the reduction in n_e_ leads to an increase in the electron mean free path and a decrease in collision frequency. As a result, electrons can attain higher energies, contributing to the observed rise in T_ex_ at elevated gas flow rates [49].

Fig 4(c) shows the effect of ground electrode geometry on plasma parameters at a gas flow rate of 3 L.min^−1^ with a gas composition of air:O_2_::5:3. As expected, all plasma parameters show a maximum for the FGE configuration compared to the FWGE. This is because the FWGE produces irregular and dynamically varying electrode spacing between the power electrode and the ground electrode, which makes non-symmetric field distribution resulting in discharge discontinuity because a higher electrode gap requires higher potential for sustaining discharge that significantly influences the plasma parameters [50].

It is noted that the low electric field generally favors vibrational excitation of N_2_. However, efficient NF also requires O atoms, which are generated through dissociation processes where high T_e_ is advantageous [6]. Incorporation of FGE in the ADJ plasma leads to an increase in the effective electric field. Whereas, in FWGE configuration, the distance between the power electrode and the ground electrode varies due to gas flow, but in FGE configuration it remains constant, resulting in a more uniform and stronger electric field. This enhances NF, particularly via the Zeldovich mechanism, where N_2_ participates through vibrational excitation. As shown in Fig 4(c), both the rotational and vibrational temperatures are higher in the FGE configuration compared to FWGE, supporting this observation.

### 3.2. Plasma treated water (PAW) properties

**Fig 5:**
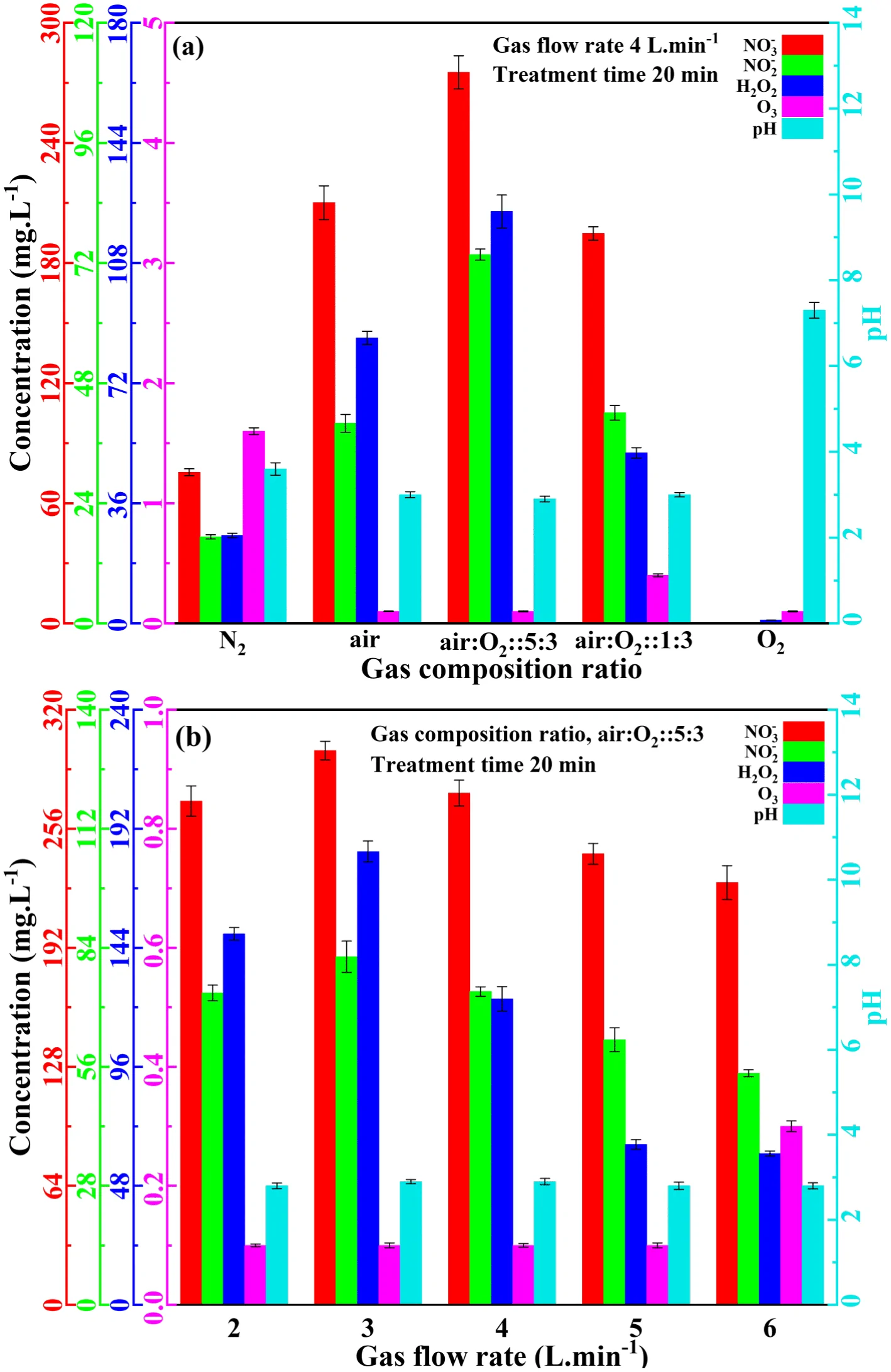
Effect of (a) gas composition ratio and (b) gas flow rate on species (${NO}_{2}^{-},\;{NO}_{3}^{-},{𝐇}_{2}{𝐎}_{2},and\;{𝐎}_{3}$) concentration and pH of treated water using fixed ground electrode configuration.

Plasma treated water was characterized in terms of gas flow rate and gas composition, and a comparison is drawn between the ADJ system with FGE and FWGEs. The characterization was performed by measuring pH, $\text{pH},\;{\text{O}}_{\text{3}},\;{\text{NO}}_{\text{2}}^{-},\;{\text{NO}}_{\text{3}}^{-},$ and ${\text{H}}_{\text{2}}{\text{O}}_{\text{2}}$ concentrations. Figs 5(a) and 5(b) present the dependences of gas composition and gas flow rate on the above parameters, respectively, for a treatment duration of 20 min.

As shown in Fig 5(a), noticeable pH variations are observed for ${\text{N}}_{\text{2}}$ and ${\text{O}}_{\text{2}}$ gases compared with mixed gas compositions. This behavior is attributed to the lower production of hydrogen ions associated with reactive nitrogen species (RNS) in the cases of pure ${\text{N}}_{\text{2}}$ and ${\text{O}}_{\text{2}}$. The minimum pH value of 2.8 was obtained when the difference between ${\text{NO}}_{\text{x}}$ and ${\text{H}}_{\text{2}}{\text{O}}_{\text{2}}$ concentrations was maximum. In the absence of ${\text{N}}_{\text{2}}$, the water becomes alkaline due to the lack of RNS formation. Fig 5(b) shows only a slight variation in pH (2.9 ± 0.1) over the investigated gas flow rates.

Fig 5(a) further indicates that increasing the fraction of ${\text{O}}_{\text{2}}$ in the gas mixture enhances ${\text{NO}}_{\text{x}}$ and the ${\text{H}}_{\text{2}}{\text{O}}_{\text{2}}$ concentrations. The maximum ${\text{H}}_{\text{2}}{\text{O}}_{\text{2}}$ and ${\text{NO}}_{\text{x}}$ concentrations ~123.43 mg.L^−1^ and 348.92 mg.L^−1^, respectively, were obtained at air:O_2_ ratio of 5:3. This trend reflects changes in discharge characteristics with increasing ${\text{O}}_{\text{2}}$ content. As ${\text{O}}_{\text{2}}$ is an electronegative gas, a higher ${\text{O}}_{\text{2}}$ fraction reduces n_e_, thereby suppressing ${\text{NO}}_{\text{x}}$ production through electron-impact processes. In addition, the reduced ${\text{N}}_{\text{2}}$ content in the gas mixture limits atomic nitrogen formation.

The dependence of ${\text{NO}}_{\text{x}}$ and ${\text{H}}_{\text{2}}{\text{O}}_{\text{2}}$ production on gas flow rate is illustrated in Fig 5(b). ${\text{NO}}_{\text{x}}$ concentration exhibits a non-monotonic behavior, increasing with gas flow rate and reaching a maximum at 3 L.min^−1^, followed by a gradual decrease. This trend results from a trade-off between gas residence time in the plasma region and gas replenishment. At low flow rate, residence time is sufficient for nitrogen activation, but gas refilling is limited. At intermediate flow rates, shorten the residence time and enhance the removal of reactive species from the discharge region.

${\text{O}}_{\text{3}}$ also exhibits non-monotonic behavior with respect to both gas composition and gas flow rate. Although ${\text{O}}_{\text{3}}$ is relatively long-lived above the water surface, dissolved ${\text{O}}_{\text{3}}$ is rapidly consumed through reactions with ${\text{H}}_{\text{2}}{\text{O}}_{\text{2}}$ (R13) and ${\text{NO}}_{\text{2}}^{-}$ (R14) [51]. ${\text{O}}_{\text{3}}$ decomposition via ${\text{H}}_{\text{2}}{\text{O}}_{\text{2}}$ proceeds slowly at low pH but becomes significant at pH>5 according to R13:


$$\begin{array}{c} {\text{O}}_{\text{3}}+{\text{H}}_{\text{2}}{\text{O}}_{\text{2}}+{\text{OH}}^{-}\to \text{ OH}\cdot +{\text{HO}}_{\text{2}}+{\text{OH}}^{-}+{\text{O}}_{\text{2}} \end{array}$$ (R13)

${\text{O}}_{\text{3}}$ decomposition by ${\text{NO}}_{\text{2}}^{-}$ follows as R14:


$$\begin{array}{c} {\text{O}}_{\text{3}}+{\text{NO}}_{\text{2}}^{-}\to \;{\text{NO}}_{\text{3}}^{-}+{\text{O}}_{\text{2}} \end{array}$$ (R14)

The generation of ${\text{O}}_{\text{3}}$ in ${\text{N}}_{\text{2}}$ plasma can be attributed to the diffusion, evaporation, or sputtering of ${\text{H}}_{\text{2}}\text{O}$ molecules into the discharge zone, leading to the formation of atomic oxygen and OH radicals. Optical emission spectra confirm the presence of OH radicals in the range of 306–309 nm and atomic oxygen lines at 822 and 868 nm [25,52].

The probable reaction pathways responsible for the formation of ${\text{O}}_{\text{3}}$ and ${\text{H}}_{\text{2}}{\text{O}}_{\text{2}}$ in water are presented below [53].


$$\begin{array}{c} {\text{H}}_{\text{2}}{\text{O}}_{\left(\text{liq}\right)}+\text{e}\to {\text{H}}_{\text{2}}{\text{O}}_{\left(\text{liq}\right)}^{+}+\text{2e} \end{array}$$ (R15)

$$\begin{array}{c} {\text{H}}_{\text{2}}{\text{O}}_{\left(\text{liq}\right)}+{\text{H}}_{\text{2}}{\text{O}}_{\left(\text{liq}\right)}^{+}\to {\text{H}}_{\text{3}}{\text{O}}_{\left(\text{liq}\right)}^{+}+{\text{OH}}_{\left(\text{liq}\right)}. \end{array}$$ (R16)

${\text{H}}_{\text{2}}{\text{O}}_{\text{2}}$ can dominantly (≈ 99.6%) be generated in the liquid phase by the following reaction (R17) at the plasma-liquid interface [54].


$$\begin{array}{c} {\text{OH}}_{\left(\text{liq}\right)}+{\text{OH}}_{\left(\text{liq}\right)}\to {\text{H}}_{\text{2}}{\text{O}}_{\text{2}(\text{liq})}. \end{array}$$ (R17)

The dominant production pathway of ${\text{H}}^{+}$, which leads to a decrease in pH, occurs through the reaction (R18) at the plasma-liquid interface [55].


$$\begin{array}{c} {\text{NO}}_{\text{2}(\text{liq})}+{\text{NO}}_{\text{2}(\text{liq})}+{\text{H}}_{\text{2}}{\text{O}}_{\left(\text{liq}\right)}\to \text{N}{\text{O}}_{\text{2}(\text{liq})}^{-}+\text{N}{\text{O}}_{\text{3}(\text{liq})}^{-}+\text{2}{\text{H}}_{\left(\text{liq}\right)}^{+}. \end{array}$$ (R18)

The primary pathways for the generations of $\text{N}{\text{O}}_{\text{2}}^{-}$ and $\text{N}{\text{O}}_{\text{3}}^{-}$ through the reactions of $\text{N}{\text{O}}_{\left(\text{liq}\right)}$ and $\text{N}{\text{O}}_{\text{2}(\text{liq})}$ in the liquid phase (R19 and R20) [55].


$$\begin{array}{c} \text{N}{\text{O}}_{\left(\text{liq}\right)}+\text{N}{\text{O}}_{\text{2}(\text{liq})}+{\text{H}}_{\text{2}}{\text{O}}_{\left(\text{liq}\right)}\to \text{N}{\text{O}}_{\text{2}(\text{liq})}^{-}+\text{N}{\text{O}}_{\text{3}(\text{liq})}^{-}+{\text{2H}}_{\left(\text{liq}\right)}^{\;+}, \end{array}$$ (R19)

$$\begin{array}{c} {\text{4NO}}_{\text{2}(\text{liq})}+{\text{O}}_{\text{2}(\text{liq})}+{\text{2H}}_{\text{2}}{\text{O}}_{\left(\text{liq}\right)}\to {\text{4NO}}_{\text{3}(\text{liq})}^{-}+{\text{4H}}_{\left(\text{liq}\right)}^{\;+}. \end{array}$$ (R20)

However, the concentrations of ${\text{NO}}_{\text{2}}^{-}$, ${\text{NO}}_{\text{3}}^{-}$, and ${\text{H}}_{\text{2}}{\text{O}}_{\text{2}}$ evolve in the treated water with time. The two dominant pathways for ${\text{NO}}_{\text{2}}^{-}$ conversion are disproportionation of ${\text{NO}}_{\text{2}}^{-}$ when pH is lower than 3.5 (R21), and ${\text{NO}}_{\text{2}}^{-}$ and ${\text{H}}_{\text{2}}{\text{O}}_{\text{2}}$ reaction (R22) [45]. Also, in lower pH conditions, ${\text{NO}}_{\text{2}}^{-}$ forms peroxynitrite (O=NOOH) by reacting with ${\text{H}}_{\text{2}}{\text{O}}_{\text{2}}$ (R23) [56]. The ${\text{H}}_{\text{2}}{\text{O}}_{\text{2}}$ and ${\text{O}}_{\text{3}}$ also diminish through the peroxone process (R24) [19,57].


$$\begin{array}{c} {\text{3NO}}_{\text{2}(\text{liq})}+{\text{2H}}_{\left(\text{liq}\right)}^{\;+}\to {\text{2NO}}_{\left(\text{liq}\right)}+\text{N}{\text{O}}_{\text{3}(\text{liq})}^{-}+{\text{H}}_{\text{2}}{\text{O}}_{\left(\text{liq}\right)}, \end{array}$$ (R21)

$$\begin{array}{c} {\text{NO}}_{\text{2}(\text{liq})}^{-}+{\text{H}}_{\text{2}}{\text{O}}_{\text{2}(\text{liq})}\to \text{N}{\text{O}}_{\text{3}(\text{liq})}^{-}+{\text{H}}_{\text{2}}{\text{O}}_{\left(\text{liq}\right)}. \end{array}$$ (R22)

$$\begin{array}{c} {\text{NO}}_{\text{2}(\text{liq})}^{-}+{\text{H}}_{\text{2}}{\text{O}}_{\text{2}(\text{liq})}\to \text{O}=\text{NOOH}+{\text{H}}_{\text{2}}{\text{O}}_{\left(\text{liq}\right)}. \end{array}$$ (R23)

$$\begin{array}{c} {\text{H}}_{\text{2}}{\text{O}}_{\text{2}(\text{liq})}+{\text{O}}_{\text{3}(\text{liq})}\to \text{O}{\text{H}}_{\left(\text{liq}\right)}+\text{H}{\text{O}}_{\text{2}(\text{liq})}+{\text{O}}_{\text{2}(\text{liq})} \end{array}$$ (R24)

**Fig 6:**
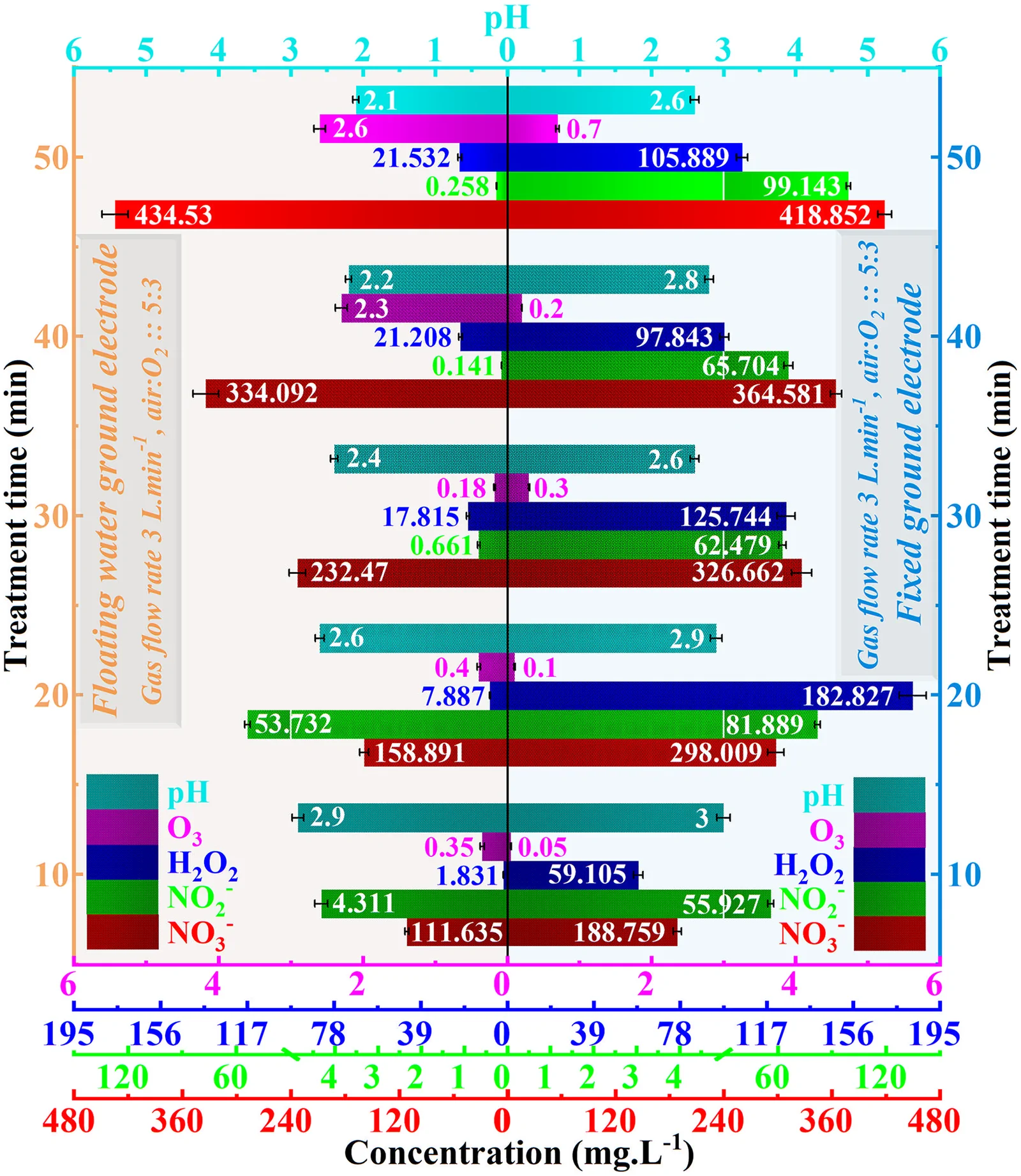
Temporal evolution (vertical axes) and comparison of reactive species (NO_2_^-^, NO_3_^-^, H_2_O_2_, and O_3_) concentration (lower horizontal axes) and pH (upper horizontal axes) of the treated water between fixed ground electrode (left side) and floating water ground electrode (right side) configuration.

Fig 6 shows the temporal evolution of reactive species concentration and pH for FWGE and FGE configurations at a gas flow rate of 3 L.min^−1^ with air:O_2_ ratio of 5:3. In the FWGE configuration, ${\text{NO}}_{\text{3}}^{-}$ concentration increased monotonically from 111.6 mg.L^−1^ at 10 min to 434.5 mg.L^−1^ at 50 min, while pH decreased from 2.9 to 2.1. The monotonic increment of ${\text{NO}}_{\text{3}}^{-}$ is due to the decomposition of ${\text{O}}_{\text{3}}$ by ${\text{NO}}_{\text{2}}^{-}$ through reaction R14, as seen from the figure that ${\text{O}}_{\text{3}}$ increases dramatically with treatment time. ${\text{H}}_{\text{2}}{\text{O}}_{\text{2}}$ concentration remained low throughout the treatment, reaching a maximum of 21.5 mg.L^−1^ at 50 min. ${\text{NO}}_{\text{2}}^{-}$concentration exhibits a transient peak of 53.7 mg.L^−1^ at 20 min, followed by a rapid decline to negligible levels. This decrease is attributed to enhanced conversion of $\;{\text{NO}}_{\text{2}}^{-}$ to ${\text{NO}}_{\text{3}}^{-}$ under acidic conditions via proton and ${\text{H}}_{\text{2}}{\text{O}}_{\text{2}}$ assisted reactions (R25, R26) [45]:


$$\begin{array}{c} \text{3}{\text{NO}}_{\text{2}}^{-}+\text{2}{\text{H}}^{+}\to \text{2NO}+{\text{NO}}_{\text{3}}^{-}+{\text{H}}_{\text{2}}\text{O} \end{array}$$ (R25)

$$\begin{array}{c} {\text{NO}}_{\text{2}}^{-}+{\text{H}}_{\text{2}}{\text{O}}_{\text{2}}\to {\text{NO}}_{\text{3}}^{-}+{\text{H}}_{\text{2}}\text{O} \end{array}$$ (R26)

Additionally, increasing water temperature during prolonged treatment reduces the Henry’s law constant, thereby decreasing ${\text{NO}}_{\text{2}}^{-}$ solubility.

In contrast, the FGE configuration produced higher concentrations of ${\text{NO}}_{\text{2}}^{-}$ and ${\text{H}}_{\text{2}}{\text{O}}_{\text{2}}$. Although ${\text{NO}}_{\text{3}}^{-}$ followed a similar increasing trend, reaching 418.8 mg.L^−1^ at 50 min, ${\text{H}}_{\text{2}}{\text{O}}_{\text{2}}$ peaked at 125.7 mg.L^−1^ at 30 min and ${\text{NO}}_{\text{2}}^{-}$ remained significant throughout the treatment, attaining 99.1 mg.L^−1^ at 50 min. The pH decrease was less pronounced and stabilized at approximately 2.6. The higher persistence of ${\text{NO}}_{\text{2}}^{-}$ indicates a lower conversion rate to ${\text{NO}}_{\text{3}}^{-}$, which can be attributed to the relatively higher pH and lower water temperature.

${\text{O}}_{\text{3}}$ concentration was higher in the FWGE configuration compared to the FGE case. This behavior is attributed to the higher ${\text{NO}}_{\text{2}}^{-}$ concentration in the fixed electrode configuration, which enhances ${\text{O}}_{\text{3}}$ consumption through reactions R14 leading to ${\text{NO}}_{\text{3}}^{-}$ formation.

### 3.3. Efficiency of NOx synthesis

**Fig 7:**
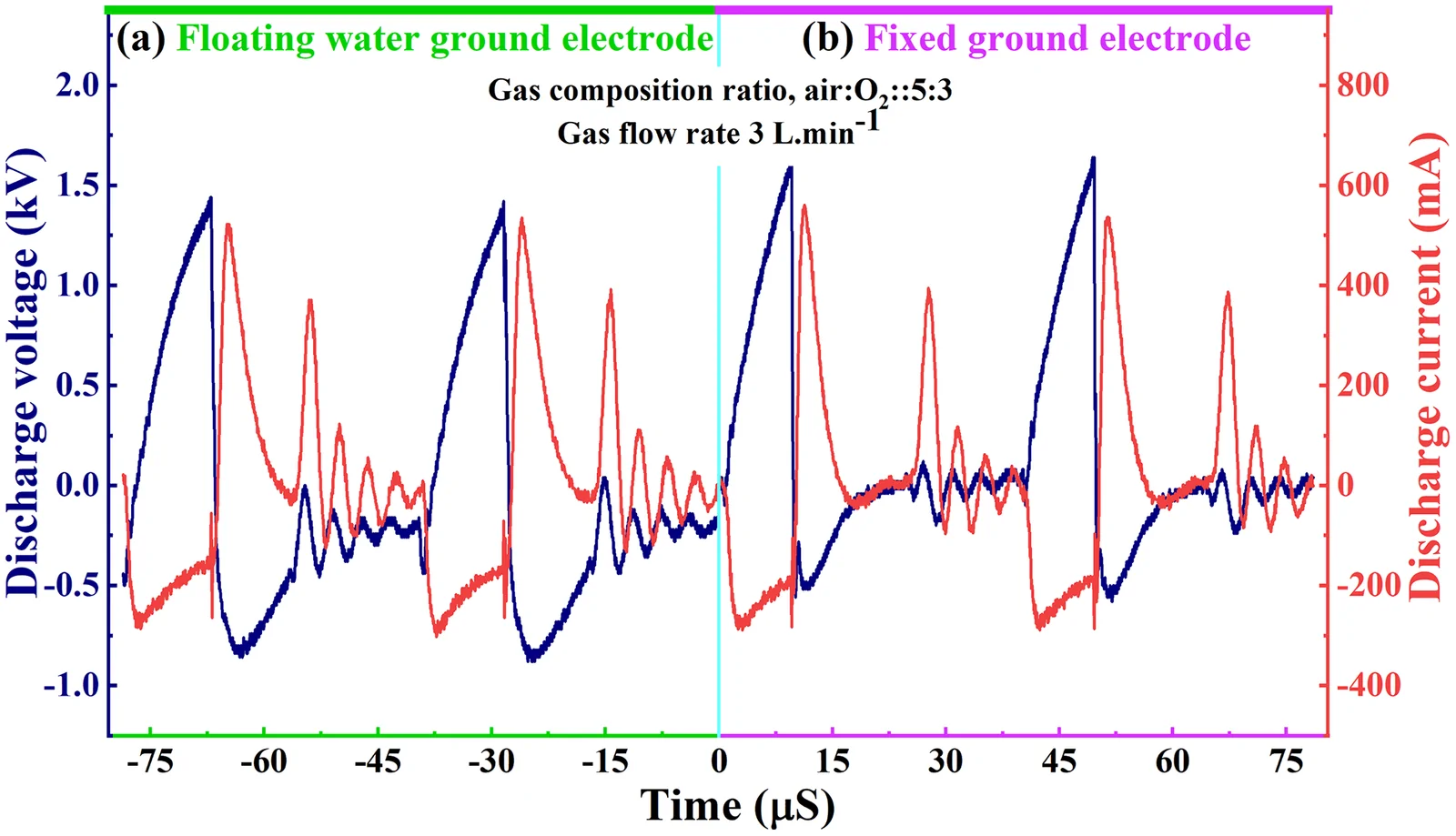
Discharge voltage and current waveforms of atmospheric pressure arc jet discharge plasma of (a) floating water ground electrode and (b) fixed ground electrode.

**Fig 8:**
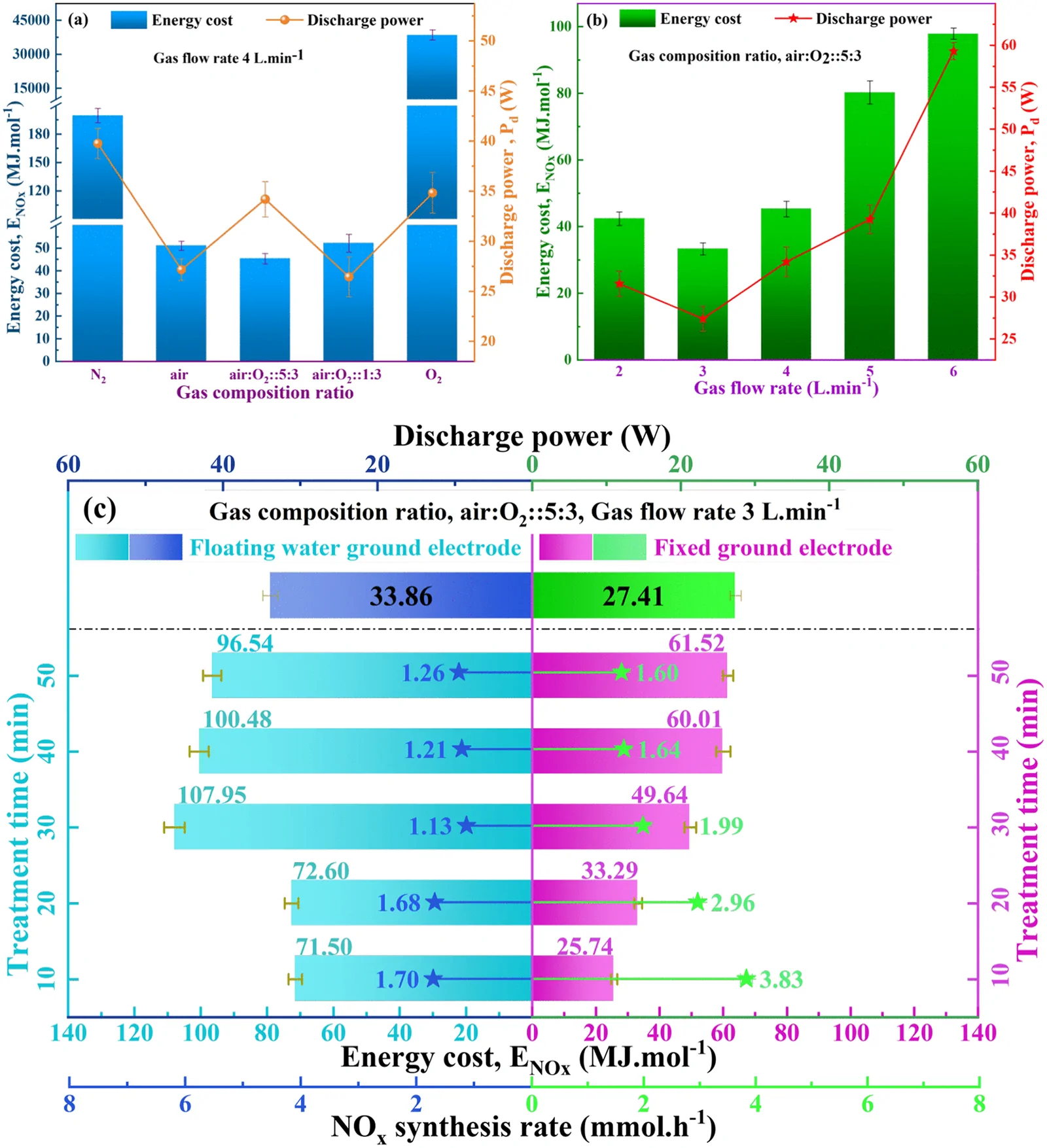
Consequences of (a) gas composition variation and (b) gas flow rate variation on ${NO}_{𝐱}$ production cost and discharge power using fixed ground electrode configuration. (c) Time-dependent consequences of floating water ground electrode and fixed ground electrode configuration on ${\text{NO}}_{\text{x}}$ production cost, ${\text{NO}}_{\text{x}}$ synthesis rate, and discharge power.

The energy cost usually means the amount of energy consumed for the production of NO_x_ (${\text{NO}}_{\text{2}}^{-}$+${\text{NO}}_{\text{3}}^{-}$) per unit mole in PAW. The power is calculated from the discharge voltage and current as depicted in Fig 7. Fig 8 presents the effect of discharge power and NO_x_ energy cost with gas composition, gas flow rate, treatment time, and electrode configurations. As shown in Fig 8(a), the minimum energy cost is obtained at an air:O_2_ ratio of 5:3; the higher NO_x_ yield at a 5:3 ratio results in the lowest overall energy cost. Fig 8(b) indicates that both discharge power and NO_x_ energy cost are lowest at a gas flow rate of 3 L.min^−1^ and highest at 6 L.min^−1^. These results suggest that, in our experimental condition, an optimal operating condition for NO_x_ generation is achieved using a gas flow rate of 3 L.min^−1^ with air:O_2_ ratio of 5:3. Fig 8(c) compares the energy cost and discharge power for the FGE and FWGE electrode configurations. For the FGE, the energy cost ranges from a minimum of 25.74 MJ.mol^−1^ at 10 min of treatment to a maximum of 61.52 MJ.mol^−1^ at 50 min. In contrast, the FWGE exhibits significantly higher energy costs, approximately 2 times greater, varying from 71.50 MJ.mol^−1^ at 10 min to a peak of 107.95 MJ.mol^−1^ at 30 min. In the case of FGE configurations, the energy cost increases with treatment time, whereas in FWGE, the energy cost initially increases with treatment time and subsequently decreases. Which can be attributed to the fact that as the treatment time increases, the NO_x_ formation rate decreases for the FEG configuration, while for the FWGE configuration, the NO_x_ formation rate decreases initially and subsequently increases with treatment time beyond 30 min. Throughout the treatment period, the discharge power remains constant at 27.41 W for the FGE and 33.86 W for the FWGE.

A comparative summary of the production rate and energy cost by arc jet discharge plasma system is presented in Table 3.

**Table 3:** Comparison of the outcomes of the AJD plasma system with other recent studies.

Plasma source	Feedstock gas	Flow rate (L.min ^− 1^)	Power (w)	Production rate (mmol.h ^− 1^)	Energy cost (MJ.mol ^− 1^)	Ref.
Plasma jet	N_2_, H_2_O	30	0.1	0.678	14.89	[58]
Gliding arc discharge	Air	3.2	58.63	0.284	744.21	[59]
Needle bubble discharge	N_2_:O_2_::33.33%:66.67%	2	52.5	1.245	151.82	[60]
Plasma jet	N_2_, H_2_O	1.4, with 100% H_2_O saturation	0.1	0.024	15	[13]
Bubble discharge	N_2_	0.5	130	0.672	696.4	[9]
Streamer spark discharge	Synthetic air	2	13	2.9	62.5	[12]
DBD plasma with catalyst	Air	2.5	200	9.72	61	[61]
AJD with FGE	air:O_2_::5:3	3	27.41	3.83	25.74	This work
AJD with FWGE	air:O_2_::5:3	3	33.86	1.70	71.50	This work

## 4. Conclusion

An arc jet discharge plasma source was designed to study the discharge performance via NO_x_ production efficiency using FGE and FWGE configurations. Experiments were conducted at a gas flow rate of 3 L.min^−1^ and a gas composition ratio of air:O_2_ = 5:3. For the FGE configuration, the measured plasma parameters were T_r_ = 1938 K, ${\text{T}}_{\text{v}}^{\text{C}}$ = 4736 K, T_ex_ = 10959 K, and n_e_ = 1.37 × 10^16^ cm^-3^, whereas for the FWGE configuration they were T_r_ = 1752 K, ${\text{T}}_{\text{v}}^{\text{C}}$ = 4753 K, T_ex_ = 10381 K, and n_e_ = 1.11 × 10^16^ cm^-3^.

The NO_x_ production rate decreased and energy cost increased with treatment time, and they reached the values from 3.83 mmol.h^−1^ to 1.60 mmol.h^−1^ and 25.74 MJ.mol^−1^ to 61.52 MJ.mol^−1^ for the FGE configuration, whereas the FWGE configurations yield NO_x_ from 1.70 mmol.h^−1^ to 1.26 mmol.h^−1^ with a significantly higher energy cost from 71.50 MJ.mol^−1^ to 107.95 MJ.mol^−1^.

Overall, the highest NO_x_ production efficiency was obtained using both the FGE and FWGE configurations under conditions of air:O_2_::5:3, a gas flow rate of 3 L.min^−1^, and a treatment time of 10 min. The FGE provided a more stable and continuous discharge, leading to improved discharge performance and process sustainability, resulting in approximately two times reduced NO_x_ synthesis energy cost.

The relatively higher energy cost in the present study can be attributed by several factors. Firstly, NO_x_ species are primarily generated in the gas phase and subsequently transferred into the liquid phase through dissolution. However, the transfer efficiency is limited by species life-time, gas-liquid mass transport and residence time. So, a significant portion of the NO_x_ may remain in the gas phase without dissolving into the liquid. Secondly, the system operated without additional optimization strategies such as plasma-catalyst coupling, advanced power modulation, or optimized gas-liquid flow dynamics, which are known to significantly improve energy efficiency. Thirdly, a part of the input energy is lost through heat dissipation and non-productive excitation processes.

## Supporting information

S1 Table Dataset of Fig 2(c). (PDF)

S2 Table Dataset of Figs 3(a) and (b). (PDF)

S3 Table Dataset of Figs 4(a), (b) and (c). (PDF)

S4 Table Dataset of Figs 5(a) and (b). (PDF)

S5 Table Dataset of Fig 6. (PDF)

S6 Table Dataset of Fig 7. (PDF)

S7 Table Dataset of Figs 8(a), (b) and (c). (PDF)
