# Supplementary material for: Nitrogen fixation efficiency enhancement using plasma jet: Impact of grounded electrode
Source: PLoS One. 2026 Sep 1;21(9):e0346219. doi: 10.1371/journal.pone.0346219 (PMC13533402; doi:10.1371/journal.pone.0346219)
Supplement: Supplementary file 1 [file pone.0346219.s001.pdf]

Data of Fig 2(c).

Measured data		Voigt fitted data at 750.39 nm		Voigt fitted data at 751.465 nm		Cumulative Voigt fitted data of measured data	
Wavelength (nm)	Intensity (a.u.)	Wavelength (nm)	Intensity (a.u.)	Wavelength (nm)	Intensity (a.u.)	Wavelength (nm)	Intensity (a.u.)
741.01	982.34	741.01	1.52404	741.01	2.16776	741.01	3.6918
741.41	980.43	742.42218	2.16558	742.61399	3.00739	741.10487	3.76454
741.81	982.79	743.83435	3.33088	744.21799	4.44254	741.19975	3.83947
742.21	989.76333	745.24653	5.8397	745.82198	7.19148	741.29462	3.91669
742.62	990.78667	746.6587	13.98111	747.42597	13.44365	741.3895	3.9963
743.02	995.4	746.77136	15.54169	747.55806	14.28332	741.48437	4.07839
743.42	996.83333	746.98912	19.69148	747.81355	16.13822	741.57925	4.16307
743.83	999.72333	747.19697	25.88233	748.05769	18.25419	741.67412	4.25045
744.23	992.00333	747.39516	35.21065	748.29074	20.66648	741.76899	4.34065
744.63	993.47	747.58392	49.09028	748.51295	23.41333	741.86387	4.4338
745.04	995.27	747.76347	69.1417	748.72458	26.53536	741.95874	4.53002
745.44	1002.19667	747.93404	96.9803	748.92589	30.07448	742.05362	4.62946
745.84	1001.62	748.09587	133.93914	749.11713	34.07208	742.14849	4.73227
746.24	1001.03667	748.24918	180.79189	749.29857	38.56653	742.24337	4.8386
746.65	1005.58333	748.39421	237.54903	749.47046	43.58979	742.33824	4.94862
747.05	1012.64	748.53118	303.38047	749.63307	49.16302	742.43312	5.0625
747.45	1026.95667	748.66033	376.68125	749.78664	55.29147	742.52799	5.18043
747.85	1089.79333	748.78189	455.2587	749.93144	61.95897	742.62286	5.30262
748.26	1125.45667	748.89607	536.59278	750.06772	69.12268	742.71774	5.42927
748.66	1361.42	749.00313	618.1131	750.19575	76.70915	742.81261	5.5606
749.06	1700.71	749.10328	697.44304	750.31578	84.61276	742.90749	5.69687
749.46	2023.29667	749.19676	772.57953	750.42806	92.69786	743.00236	5.83832
749.86	2180.98667	749.28379	841.99635	750.53287	100.80517	743.09724	5.98523
750.27	2212.62	749.36461	904.67538	750.63045	108.76231	743.19211	6.13789
750.67	2007.85667	749.43944	960.08058	750.72106	116.39753	743.28698	6.29662
751.07	1849.67333	749.50852	1008.09349	750.80496	123.55423	743.38186	6.46175
751.47	1489.22333	749.57208	1048.92866	750.88242	130.10415	743.47673	6.63365
751.87	1246.67	749.63034	1083.04395	750.95368	135.95663	743.57161	6.8127
752.28	1121.12667	749.68354	1111.05635	751.019	141.06279	743.66648	6.99932
752.68	1066.39	749.73191	1133.66959	751.07865	145.41439	743.76136	7.19396
753.08	1076.25	749.77568	1151.61618	751.13288	149.0384	743.85623	7.3971
753.48	1044.79333	749.81507	1165.61423	751.18195	151.98885	743.95111	7.60926
753.88	1053.8	749.85032	1176.33765	751.22612	154.33769	744.04598	7.83103
754.28	1058.57	749.88166	1184.39755	751.26564	156.16614	744.14085	8.063
754.68	1033.17667	749.90931	1190.33256	751.30077	157.55746	744.23573	8.30585
755.08	1021.37667	749.93352	1194.60583	751.33178	158.5915	744.3306	8.5603
755.49	1029.33333	749.9545	1197.6066	751.35892	159.34108	744.42548	8.82715

755.89	1020.30667	749.97249	1199.65491	751.38244	159.87002	744.52035	9.10727
756.29	1017.64667	749.98772	1201.00822	751.40261	160.23229	744.61523	9.40159
756.69	1016.77333	750.00041	1201.86884	751.41968	160.47218	744.7101	9.71118
757.09	1003.9	750.01081	1202.3918	751.43392	160.62497	744.80497	10.03716
757.49	1003.23	750.01913	1202.69247	751.44557	160.7179	744.89985	10.38082
757.89	1000.08667	750.02561	1202.85383	751.4549	160.77135	744.99472	10.74355
758.29	1006.43333	750.03048	1202.93312	751.46217	160.80005	745.0896	11.12692
758.69	998.07333	750.03397	1202.96778	751.46763	160.81416	745.18447	11.53269
759.09	990.24333	750.03631	1202.98066	751.47155	160.82032	745.27935	11.96281
759.49	996.93333	750.03773	1202.98442	751.47417	160.82261	745.37422	12.41952
759.89	999.64333	750.03846	1202.98516	751.47576	160.82328	745.4691	12.90532
760.29	999.01	750.03873	1202.98523	751.47658	160.82341	745.56397	13.42314
760.69	993.44333	750.03877	1202.98523	751.47688	160.82343	745.65884	13.97631
		750.03881	1202.98523	751.47692	160.82343	745.75372	14.56902
		750.03907	1202.98517	751.47696	160.82343	745.84859	15.2054
		750.03977	1202.98447	751.47726	160.82341	745.94347	15.89124
		750.04115	1202.98092	751.47805	160.82329	746.03834	16.63331
		750.04342	1202.96878	751.4796	160.82266	746.13322	17.43989
		750.04681	1202.93611	751.48215	160.82049	746.22809	18.32134
		750.05154	1202.86136	751.48595	160.81466	746.32296	19.29069
		750.05784	1202.70925	751.49126	160.80133	746.41784	20.36459
		750.06592	1202.4258	751.49833	160.7742	746.51271	21.56441
		750.07601	1201.9328	751.5074	160.72367	746.60759	22.91779
		750.08834	1201.12143	751.51873	160.63582	746.70246	24.46051
		750.10312	1199.84552	751.53257	160.49137	746.79734	26.23897
		750.12059	1197.91417	751.54917	160.26454	746.89221	28.31318
		750.14096	1195.08442	751.56878	159.92193	746.98709	30.76035
		750.16446	1191.05393	751.59165	159.42154	747.08196	33.67914
		750.19131	1185.45469	751.61804	158.7121	747.17683	37.1945
		750.22174	1177.84809	751.64818	157.73285	747.27171	41.46292
		750.25596	1167.72293	751.68234	156.41415	747.36658	46.67796
		750.29421	1154.49745	751.72077	154.67924	747.46146	53.0756
		750.3367	1137.52728	751.76371	152.44738	747.55633	60.93891
		750.38366	1116.12145	751.81141	149.6388	747.65121	70.60142
		750.43532	1089.56853	751.86414	146.18127	747.74608	82.4483
		750.49189	1057.17508	751.92213	142.01824	747.84095	96.91446
		750.5536	1018.31776	751.98564	137.11748	747.93583	114.47856
		750.62067	972.50939	752.05492	131.47924	748.0307	135.65195
		750.69333	919.47692	752.13023	125.14211	748.12558	160.96184
		750.7718	859.24637	752.2118	118.18509	748.22045	190.9281
		750.8563	792.22575	752.2999	110.72487	748.31533	226.03374
		750.94706	719.27311	752.39477	102.90796	748.4102	266.68956
		751.04429	641.73305	752.49666	94.89894	748.50508	313.19423
		751.14824	561.42392	752.60583	86.86665	748.59995	365.69181
		751.25911	480.56028	752.72253	78.97066	748.69482	424.12959

		751.37712	401.60315	752.847	71.35026	748.7897	488.21937
		751.50252	327.04394	752.9795	64.1171	748.88457	557.40648
		751.63551	259.14621	753.12028	57.35208	748.97945	630.84996
		751.77632	199.68646	753.26959	51.10584	749.07432	707.41801
		751.92517	149.74634	753.42768	45.40193	749.1692	785.70127
		752.0823	109.60585	753.5948	40.24152	749.26407	864.0458
		752.24791	78.7681	753.7712	35.60872	749.35894	940.60607
		752.42224	56.11366	753.95714	31.4756	749.45382	1013.41637
		752.6055	40.14586	754.15286	27.80667	749.54869	1080.47746
		752.79793	29.26343	754.35861	24.56241	749.64357	1139.85334
		752.99974	21.99292	754.57465	21.70205	749.73844	1189.77191
		753.21117	17.13499	754.80123	19.18543	749.83332	1228.72191
		753.32055	15.32456	755.03859	16.97437	749.92819	1255.53888
		754.63444	6.41792	755.28699	15.03334	750.02307	1269.47299
		755.94833	3.69524	755.41541	14.15384	750.11794	1270.23296
		757.26222	2.41837	756.90694	7.77022	750.21281	1258.00218
		758.57611	1.71006	758.39847	4.87275	750.30769	1233.4251
		759.89	1.27467	759.89	3.33076	750.40256	1197.56456
						750.49744	1151.83295
						750.59231	1097.90228
						750.68719	1037.60019
						750.78206	972.80033
						750.87693	905.31646
						750.97181	836.80981
						751.06668	768.7186
						751.16156	702.21653
						751.25643	638.20345
						751.35131	577.32577
						751.44618	520.01856
						751.54106	466.55661
						751.63593	417.10097
						751.7308	371.73152
						751.82568	330.46288
						751.92055	293.24768
						752.01543	259.97471
						752.1103	230.46895

						752.20518	204.49733
						752.30005	181.78068
						752.39492	162.00967
						752.4898	144.86185
						752.58467	130.01728
						752.67955	117.17106
						752.77442	106.04204
						752.8693	96.37797
						752.96417	87.95754
						753.05905	80.59006
						753.15392	74.11357
						753.24879	68.392
						753.34367	63.31191
						753.43854	58.77917
						753.53342	54.71588
						753.62829	51.05747
						753.72317	47.75036
						753.81804	44.74985
						753.91291	42.01845
						754.00779	39.52452
						754.10266	37.24116
						754.19754	35.14535
						754.29241	33.21723
						754.38729	31.4396
						754.48216	29.79718
						754.57704	28.27732
						754.67191	26.86814
						754.76678	25.55939
						754.86166	24.34195
						754.95653	23.2077

						755.05141	22.1494
						755.14628	21.16056
						755.24116	20.23535
						755.33603	19.36853
						755.4309	18.55539
						755.52578	17.79167
						755.62065	17.0735
						755.71553	16.39739
						755.8104	15.76018
						755.90528	15.15898
						756.00015	14.59116
						756.09503	14.05434
						756.1899	13.54632
						756.28477	13.0651
						756.37965	12.60886
						756.47452	12.17592
						756.5694	11.76472
						756.66427	11.37386
						756.75915	11.00203
						756.85402	10.64801
						756.94889	10.31071
						757.04377	9.9891
						757.13864	9.68221
						757.23352	9.38919
						757.32839	9.1092
						757.42327	8.84149
						757.51814	8.58536
						757.61302	8.34015
						757.70789	8.10526
						757.80276	7.88012

						757.89764	7.6642
						757.99251	7.457
						758.08739	7.25806
						758.18226	7.06696
						758.27714	6.88328
						758.37201	6.70666
						758.46688	6.53673
						758.56176	6.37316
						758.65663	6.21564
						758.75151	6.06387
						758.84638	5.91759
						758.94126	5.77652
						759.03613	5.64043
						759.13101	5.50909
						759.22588	5.38227
						759.32075	5.25977
						759.41563	5.14141
						759.5105	5.02699
						759.60538	4.91634
						759.70025	4.8093
						759.79513	4.70571
						759.89	4.60543
