# Supplementary material for: Nitrogen fixation efficiency enhancement using plasma jet: Impact of grounded electrode
Source: PLoS One. 2026 Sep 1;21(9):e0346219. doi: 10.1371/journal.pone.0346219 (PMC13533402; doi:10.1371/journal.pone.0346219)
Supplement: Supplementary file 2 [file pone.0346219.s002.pdf]

Data of Fig 3(a).

Wavelength (nm)	Intensity (a.u.)	
	Fixed ground electrode config.	Floating water ground electrode config.
301.76	0	0
302.22	0	0
302.68	0	0
303.13	0	0
303.59	0	0
304.05	0	0
304.51	0	0
304.96	97.41667	93.65667
305.42	131.83	233.52
305.88	225.48667	367.09667
306.34	270.92333	298.04333
306.8	315.12667	445.43667
307.25	480.34333	673.17333
307.71	782.90333	983.27333
308.17	989.54667	1266.75667
308.62	1235.87667	1452.81667
309.08	1452.31333	1535.93333
309.54	1802.33333	1669.76333
310	2098.88	1808.11
310.45	2429.81667	1907.04667
310.91	2753.22333	2187.51333
311.37	3278.25333	2491.08333
311.83	4079.99	3000.55
312.28	4410.43	3200.67
312.74	4426.24667	3161.49667
313.2	4563.34333	3283.52333
313.65	5405.76333	3780.94333
314.11	6635.60667	4673.32667
314.57	6485.95333	4604.27333
315.02	4869.67667	3455.02667
315.48	2291.71667	1772.70667
315.94	900.41333	773.86333
316.39	515.74333	438.91333
316.85	366.34333	342.23333
317.31	314.36667	287.24667
317.76	282.23	231.01
318.22	278.21	275.95
318.68	278.46	206.15
319.13	282.73	152.41
319.59	224.22333	187.31333

320.04	243.30333	165.72333
320.5	254.11	114.75
320.96	183.54667	125.54667
321.41	273.68667	0
321.87	234.77667	84.86667
322.32	331.69333	153.16333
322.78	373.37667	205.39667
323.24	497.42	260.89
323.69	724.14333	367.09333
324.15	955.65667	431.37667
324.6	1125.9	612.16
325.06	1523.37667	782.14667
325.52	1811.88	1002.11
325.97	2049.66667	1131.41667
326.43	2198.05333	1266.25333
326.88	2322.34667	1283.57667
327.34	2445.38	1384.02
327.79	2152.86	1209.76
328.25	1965.04667	984.28667
328.7	2217.64667	1197.70667
329.16	2404.96	1364.69
329.62	2712.3	1522.12
330.07	2924.97667	1694.87667
330.53	3191.12667	1809.61667
330.98	3725.19667	2219.39667
331.44	4717.01	2728.37
331.89	6041.52	3613.71
332.35	7434.08333	4488.01333
332.8	9166.86667	5658.10667
333.26	11348.09667	6994.16667
333.71	14588.44	9088.77
334.17	18852.73667	11813.36667
334.62	25108.44	15797.95
335.08	31588.87333	20166.95333
335.53	34440.27667	22111.40667
335.98	29171.11	18621.48
336.44	17079.53	10836.38
336.89	6401.83667	4174.40667
337.35	2484.30667	1560.78667
337.8	968.21	512.48
338.26	639.27667	282.97667
338.71	494.65667	136.09667
339.17	512.72667	215.93667
339.62	521.51667	191.58667
340.07	312.10667	29.62667

340.53	539.34	166.47
340.98	582.03	193.34
341.44	641.79	228.99
341.89	698.28667	239.53667
342.34	720.63667	357.55667
342.8	915.73	357.55
343.25	967.20333	437.65333
343.71	1109.58	485.86
344.16	1187.41	555.41
344.61	1278.56333	622.45333
345.07	1598.95667	781.64667
345.52	1859.34	936.58
345.98	2202.32667	1112.33667
346.43	2565.15667	1350.87667
346.88	3113.54	1777.98
347.34	3644.35	2063.22
347.79	4362.72333	2580.47333
348.24	5281.46667	3121.06667
348.7	6039.26333	3524.82333
349.15	6480.43667	3875.60667
349.6	6924.62	4036.56
350.06	8330.48	4906.84
350.51	10098.91667	6037.25667
350.96	12152.84667	7349.20667
351.42	15658.6	9493.79
351.87	18758.07667	11461.08667
352.32	18977.02667	11511.30667
352.77	17058.68667	10453.95667
353.23	15136.08	9319.28
353.68	15122.51	9350.16
354.13	18298.32667	11280.05667
354.59	22809.69667	14114.62667
355.04	27456.4	17211.1
355.49	34574.86	21683.3
355.94	38779.64667	24585.66667
356.4	33855.99	21456.31
356.85	22338.14333	14084.49333
357.3	9705.21	6196.45
357.75	3988.59333	2498.61333
358.21	2145.83333	1303.66333
358.66	1159.79333	554.16333
359.11	1015.92	430.62
359.56	1040.02667	416.30667
360.02	1079.94667	420.07667
360.47	1211.27333	525.78333

360.92	1212.52	585.8
361.37	1324.00333	626.47333
361.82	1702.15	884.85
362.28	1908.80333	954.40333
362.73	2170.19	1173.6
363.18	2389.63667	1243.15667
363.63	2486.31667	1307.43667
364.08	2780.84	1457.34
364.54	3141.92	1749.11
364.99	3727.96667	2034.59667
365.44	4244.46333	2346.95333
365.89	4386.82667	2419.26667
366.34	4317.78333	2439.86333
366.79	4072.46333	2270.62333
367.24	4485.25	2479.28
367.7	5150.65	2957.11
368.15	6125.38667	3555.96667
368.6	7107.66	4198.51
369.05	8551.44	5056.24
369.5	9048.6	5326.67
369.95	8423.63333	4975.89333
370.4	6874.14667	4084.00667
370.86	6373.72	3678.5
371.31	6708.17333	3977.55333
371.76	7790.38	4637.16
372.21	9086.51333	5451.20333
372.66	10806.75	6501.77
373.11	13036.69	7869.98
373.56	15151.39333	9342.88333
374.01	15139.08667	9338.10667
374.46	12739.90333	8012.34333
374.91	9024.24667	5523.77667
375.36	6929.88667	4184.19667
375.81	6629.57667	4017.21667
376.26	7294.72333	4420.22333
376.72	8566.75333	5262.13333
377.17	10008.77333	6101.53333
377.62	12117.44667	7256.55667
378.07	14160.32667	8701.33667
378.52	17140.29333	10476.81333
378.97	17045.12667	10496.14667
379.42	13119.05667	7986.23667
379.87	7043.38	4411.44
380.32	3460.3	2018.53
380.77	2117.71	1131.67

381.22	1642.64667	802.73667
381.67	1492.49333	709.08333
382.12	1566.81333	746.49333
382.57	1772.70667	840.15667
383.02	2037.86	1057.85
383.47	2255.30333	1221.06333
383.92	2730.87667	1479.67667
384.37	2823.78	1438.5
384.82	2674.88	1343.09
385.27	2650.52667	1384.26667
385.71	2747.45667	1432.98667
386.16	3082.15667	1636.61667
386.61	3476.87	1779.74
387.06	4131.97	2190.78
387.51	4608.79667	2544.81667
387.96	4651.48333	2583.73333
388.41	4134.23333	2325.61333
388.86	3516.03667	1905.53667
389.31	3328.72333	1712.19333
389.76	3332.99667	1789.53667
390.21	3720.93667	1978.60667
390.66	3995.88	2197.81
391.11	4336.36	2500.62
391.55	5062.51333	2852.40333
392	5680.95	3357.1
392.45	6370.20333	3772.15333
392.9	6144.47333	3572.78333
393.35	4921.90667	2903.87667
393.8	3491.18	2082.56
394.25	3027.66667	1721.48667
394.7	2947.06667	1662.73667
395.14	3252.89333	1805.09333
395.59	3560.98333	2056.69333
396.04	4123.93333	2351.47333
396.49	4705.71	2827.8
396.94	5449.70333	3229.79333
397.39	6324.25	3848.99
397.83	7300.50333	4325.06333
398.28	7111.17667	4297.68667
398.73	5862.49667	3538.63667
399.18	3753.31667	2263.33667
399.63	2532.01	1441.27
400.07	2122.23	1203.23
400.52	2145.83	1247.17
400.97	2323.10333	1248.18333

401.42	2694.71667	1542.20667
401.87	3076.12	1766.93
402.31	3620.99333	2043.63333
402.76	4301.45667	2479.28667
403.21	4795.34667	2784.10667
403.66	5706.57	3334.5
404.1	6447.53667	3727.45667
404.55	5947.61	3561.24
405	4280.86333	2602.56333
405.45	2550.08333	1423.18333
405.89	1830.71333	1004.37333
406.34	1676.54	803.49
406.79	1743.83	821.82
407.24	1842.01	921.51
407.68	1990.15333	1060.61333
408.13	1862.09333	958.16333
408.58	1553.00333	804.24333
409.02	1407.37667	709.83667
409.47	1335.55333	649.32333
409.92	1443.78	719.13
410.37	1582.62667	773.61667
410.81	1692.61333	889.62333
411.26	1880.93	1013.91
411.71	2103.9	1184.15
412.15	2342.18	1368.2
412.6	2284.68	1250.44
413.05	2031.83667	1073.66667
413.49	1543.71667	834.88667
413.94	1194.18667	608.89667
414.39	1396.82667	728.66667
414.83	1258.47333	603.12333
415.28	1340.08333	642.54333
415.72	1402.1	746
416.17	1568.56667	797.21667
416.62	1739.57	859.74
417.06	2023.79667	1050.56667
417.51	2165.16667	1112.08667
417.96	2552.6	1439.26
418.4	2603.82333	1475.41333
418.85	2396.92667	1310.69667
419.29	1788.02333	953.39333
419.74	1205.23667	619.94667
420.18	1006.38	438.41
420.63	981.01667	456.73667
421.08	1036	472.55

421.52	1117.86333	520.51333
421.97	1234.12333	544.11333
422.41	1336.81	632.5
422.86	1497.01	724.15
423.3	1645.91333	886.60333
423.75	1939.43333	1012.15333
424.19	2090.34333	1204.48333
424.64	2292.21667	1288.09667
425.09	2733.89	1455.58
425.53	2466.98	1386.03
425.98	1882.43333	1025.96333
426.42	1276.55667	582.78667
426.87	908.95667	373.37667
427.31	778.64	313.87
427.76	768.59667	315.12667
428.2	734.95	353.04
428.65	873.29333	394.21333
429.09	865.51667	385.67667
429.54	961.43	480.09
429.98	1147.23667	515.24667
430.43	1171.34667	582.27667
430.87	1273.29	617.19
431.31	1493.49667	716.11667
431.76	1553.25667	814.29667
432.2	1779.48667	913.97667
432.65	1999.45	1054.09
433.09	1826.94667	959.16667
433.54	1541.2	834.63
433.98	1204.48667	531.05667
434.42	920.50333	445.18333
434.87	661.62333	271.43333
435.31	564.2	182.29
435.76	596.59	159.69
436.2	556.17	175.01
436.65	570.99	241.8
437.09	619.95	232.76
437.53	583.03333	211.67333
437.98	667.15	301.81
438.42	651.58	275.7
438.86	769.09667	322.39667
439.31	892.37333	344.74333
439.75	929.04	381.41
440.2	905.94	429.12
440.64	661.87667	336.46667
441.08	570.98	166.47

441.53	430.37	116.26
441.97	380.40667	41.42667
442.41	391.45	119.52
442.86	447.69333	165.21333
443.3	444.68667	117.76667
443.74	460.75667	161.70667
444.19	504.94333	202.88333
444.63	571.23667	193.08667
445.07	620.69333	231.25333
445.51	659.36667	253.34667
445.96	787.17667	353.28667
446.4	806.75333	399.23333
446.84	945.86667	417.06667
447.29	945.11667	423.09667
447.73	839.9	358.56
448.17	643.04667	222.71667
448.61	457.98667	174.75667
449.06	378.14	92.65
449.5	267.16667	41.17667
449.94	380.40333	63.27333
450.38	421.83333	140.11333
450.83	360.57	80.35
451.27	380.40333	110.73333
451.71	404.25667	138.34667
452.15	428.36667	131.57667
452.6	494.40333	187.81333
453.04	529.05333	177.27333
453.48	607.38667	234.51667
453.92	621.96	240.8
454.36	747.25	389.44
454.81	836.14	335.96
455.25	909.70333	420.07333
455.69	899.15667	398.22667
456.13	818.05667	374.37667
456.57	644.80667	275.69667
457.01	425.84667	114.74667
457.46	416.06	126.8
457.9	404.01	128.31
458.34	360.32	98.93
458.78	392.20667	56.24667
459.22	435.39667	131.06667
459.66	387.18667	95.66667
460.11	495.91	123.79
460.55	375.88333	65.53333
460.99	440.41667	142.87667

461.43	470.8	181.54
461.87	508.96333	183.55333
462.31	576.76	199.37
462.75	654.34333	219.70333
463.19	630.99667	221.21667
463.63	686.23	226.73
464.07	599.1	228.49
464.52	600.86667	223.47667
464.96	540.09667	177.01667
465.4	503.93667	206.39667
465.84	528.54667	154.92667
466.28	344.74667	130.81667
466.72	394.96333	121.53333
467.16	342.23333	82.35333
467.6	287.49667	63.77667
468.04	370.61333	91.14333
468.48	408.78333	133.08333
468.92	429.36667	100.93667
469.36	425.10333	135.84333
469.8	533.32	227.49
470.24	535.33	163.96
470.68	510.47333	164.71333
471.12	395.47333	155.17333
471.56	348.26333	131.32333
472	307.33333	79.84333
472.44	272.43667	26.10667
472.88	322.14667	50.21667
473.32	375.13333	32.39333
473.76	311.86	84.37
474.2	346.25667	60.76667
474.64	328.93333	57.75333
475.08	432.88667	94.66667
475.52	423.09	160.2
475.96	422.08667	83.10667
476.4	438.90667	143.62667
476.84	472.55667	156.93667
477.28	478.08333	132.32333
477.72	483.35	141.37
478.16	604.63	217.45
478.59	572.99333	182.04333
479.03	647.82	221.46
479.47	763.31667	256.36667
479.91	693.51667	291.26667
480.35	606.63667	248.07667
480.79	467.28	148.64

481.23	457.49	128.31
481.67	438.91	109.73
482.11	517	159.19
482.54	402.24667	146.13667
482.98	463.52	152.41
483.42	502.69	168.99
483.86	497.91	174.76
484.3	496.90667	165.46667
484.74	543.36667	192.33667
485.18	546.37333	228.49333
485.61	549.38667	204.38667
486.05	549.38333	248.83333
486.49	631.24	280.97
486.93	603.62333	231.50333
487.37	691.00333	281.97333
487.81	609.9	221.21
488.24	712.85333	298.54333
488.68	740.71667	295.53667
489.12	762.57	307.59
489.56	772.86333	304.32333
490	773.11667	364.08667
490.43	793.70333	311.60333
490.87	803.49667	407.26667
491.31	650.07	329.18
491.75	652.08	249.08
492.18	605.88667	219.45667
492.62	662.62667	256.61667
493.06	609.90333	230.25333
493.5	641.29333	288.00333
493.93	614.42333	222.71333
494.37	574.75	520.08
494.81	696.78	392.45
495.24	685.48	327.68
495.68	728.66667	284.23667
496.12	790.18333	253.85333
496.56	760.30333	300.05333
496.99	716.11	261.89
497.43	672.67333	195.85333
497.87	664.39	224.48
498.3	660.37667	213.67667
498.74	680.71	202.38
499.18	720.13	229.75
499.61	718.87667	257.87667
500.05	737.70667	265.40667
500.49	749.76	233.77

500.92	721.89	249.58
501.36	721.64333	266.66333
501.8	725.40667	272.68667
502.23	694.27333	298.04333
502.67	662.62667	270.17667
503.11	683.97333	254.60333
503.54	658.10667	209.90667
503.98	808.51333	263.89333
504.41	700.8	312.86
504.85	689.99333	246.32333
505.29	679.70333	334.70333
505.72	677.95	337.47
506.16	684.22333	251.84333
506.59	595.58333	218.19333
507.03	664.14333	282.98333
507.47	647.56	272.43
507.9	623.71	308.84
508.34	648.56667	268.16667
508.77	694.26667	284.48667
509.21	659.62333	315.37333
509.64	691.00667	293.27667
510.08	740.47333	306.58333
510.51	681.47	291.27
510.95	742.98667	333.19667
511.38	730.93	318.13
511.82	746.5	329.94
512.25	730.92667	306.83667
512.69	814.04333	321.39333
513.12	764.57	317.13
513.56	787.17	301.31
513.99	758.29333	350.02333
514.43	783.91	304.08
514.86	908.69667	349.01667
515.3	822.32667	383.16667
515.73	912.72333	406.52333
516.17	968.21333	383.66333
516.6	1008.39	454.73
517.04	891.88	366.84
517.47	835.88667	356.05667
517.91	810.27	389.19
518.34	834.63	351.78
518.78	903.68333	460.00333
519.21	916.99	383.67
519.64	926.28	371.12
520.08	930.79333	400.49333

520.51	912.71667	411.03667
520.95	956.91667	394.96667
521.38	933.81333	387.68333
521.81	894.14	388.69
522.25	1040.02667	471.29667
522.68	997.08333	461.51333
523.12	1013.41	514.74
523.55	951.13333	414.80333
523.98	880.07333	412.29333
524.42	884.35	427.11
524.85	930.04333	397.48333
525.28	952.89	446.69
525.72	911.46333	370.61333
526.15	858.73667	412.79667
526.58	922.26333	366.34333
527.02	922.51333	416.31333
527.45	920.75667	391.95667
527.88	912.46333	422.08333
528.32	936.82667	427.60667
528.75	945.10667	440.41667
529.18	955.65667	394.46667
529.62	904.18333	445.44333
530.05	910.45333	380.90333
530.48	834.63	412.04
530.92	829.85333	331.94333
531.35	871.04	395.72
531.78	913.47	445.69
532.21	876.56333	343.24333
532.65	837.89	402.5
533.08	877.06333	374.63333
533.51	839.15333	409.78333
533.94	832.11667	374.87667
534.38	872.79667	370.36667
534.81	777.38333	379.65333
535.24	879.82333	482.09333
535.67	824.33	364.84
536.11	875.05667	377.89667
536.54	845.92667	338.97667
536.97	889.87	373.12
537.4	879.32667	388.18667
537.83	897.9	401.5
538.27	910.71	371.36
538.7	954.15333	377.14333
539.13	901.42	435.9
539.56	883.09	367.85

539.99	901.16667	375.38667
540.42	857.47667	381.40667
540.86	823.57667	368.59667
541.29	833.12	407.52
541.72	826.34333	411.29333
542.15	831.61333	348.01333
542.58	858.23333	345.25333
543.01	839.15333	353.28333
543.44	855.72	336.71
543.88	854.72	347.01
544.31	894.88667	390.94667
544.74	840.91333	328.68333
545.17	828.6	392.45
545.6	829.86	350.02
546.03	805.50333	349.77333
546.46	830.61333	397.48333
546.89	813.03333	307.59333
547.32	806.00333	383.42333
547.75	860.99	341.99
548.19	876.81667	345.75667
548.62	853.21333	325.16333
549.05	881.58667	347.51667
549.48	894.64333	367.34333
549.91	878.82	362.08
550.34	901.92333	379.15333
550.77	850.20333	340.98333
551.2	864.51333	313.11333
551.63	858.48	325.16
552.06	834.63333	387.93333
552.49	1075.68667	515.99667
552.92	872.79667	394.46667
553.35	959.42	389.95
553.78	910.46	403.5
554.21	964.18667	375.88667
554.64	884.59667	366.34667
555.07	882.84	375.13
555.5	886.85	397.23
555.93	923.26	406.52
556.36	878.07333	358.30333
556.79	875.80333	386.93333
557.22	956.15333	440.16333
557.65	915.73	399.74
558.08	1040.78	417.06
558.51	1074.92667	461.75667
558.93	1031.23667	481.34667

559.36	1049.81667	467.53667
559.79	1006.63333	493.65333
560.22	1008.38667	365.83667
560.65	1016.17333	423.34333
561.08	1051.32	478.83
561.51	1009.13667	450.20667
561.94	1118.11667	490.63667
562.37	1094.76	513.23
562.8	1096.02	504.69
563.22	1099.53	500.68
563.65	1168.33	516.75
564.08	1142.97333	504.94333
564.51	1123.63667	511.97667
564.94	1170.09333	536.58333
565.37	1189.17333	527.79333
565.8	1185.41	577.51
566.22	1236.63333	549.64333
566.65	1379.75	597.85
567.08	1420.42333	667.90333
567.51	1338.06333	559.18333
567.94	1327.52667	634.50667
568.37	1347.86667	629.23667
568.79	1390.54667	646.31667
569.22	1350.87333	666.14333
569.65	1310.69667	625.21667
570.08	1299.15333	617.43333
570.5	1380.75667	673.42667
570.93	1406.87333	724.40333
571.36	1511.06667	711.84667
571.79	1469.38667	737.95667
572.22	1538.18333	760.05333
572.64	1528.15	733.44
573.07	1592.43	847.44
573.5	1557.77667	821.07667
573.93	1513.33333	746.49333
574.35	1416.91333	726.91333
574.78	1384.77667	722.64667
575.21	1495.75	728.92
575.63	1483.95667	731.42667
576.06	1574.09667	825.33667
576.49	1587.65667	783.90667
576.92	1621.55667	820.06667
577.34	1644.65	845.43
577.77	1678.8	865.26
578.2	1634.60667	830.10667

578.62	1634.85667	877.06667
579.05	1585.14	880.83
579.48	1637.87333	841.66333
579.9	1453.82333	760.05333
580.33	1562.04667	795.20667
580.76	1607.99	917.24
581.18	1697.12667	946.86667
581.61	1718.47333	968.21333
582.03	1726.75667	938.83667
582.46	1748.60667	971.97667
582.89	1713.95	925.27
583.31	1757.39	1001.1
583.74	1686.58667	920.50667
584.17	1620.04667	862.25667
584.59	1578.11667	825.58667
585.02	1549.23667	861.49667
585.44	1577.36333	814.29333
585.87	1618.29333	900.42333
586.29	1590.16	926.53
586.72	1620.8	952.64
587.15	1719.73333	1010.14333
587.57	1770.70333	997.84333
588	1787.26667	984.27667
588.42	1724.75	999.34
588.85	1668.75667	974.23667
589.27	1526.88667	893.38667
589.7	1454.07333	802.48333
590.12	1389.54	793.7
590.55	1478.43	785.41
590.97	1450.55333	774.11333
591.4	1525.64	867.27
591.82	1489.98667	797.72667
592.25	1578.61667	862.24667
592.67	1601.71667	828.85667
593.1	1618.79667	862.50667
593.52	1580.37	899.41
593.95	1673.53	861.5
594.37	1573.09333	827.34333
594.8	1528.64667	791.94667
595.22	1388.03667	728.16667
595.65	1468.13333	741.98333
596.07	1424.69667	729.42667
596.5	1426.20333	730.93333
596.92	1490.73333	753.27333
597.34	1488.98	753.78

597.77	1415.40667	745.73667
598.19	1435.99667	772.35667
598.62	1368.45667	764.32667
599.04	1310.19667	787.42667
599.46	1347.61	723.9
599.89	1285.59333	709.33333
600.31	1268.26	682.22
600.74	1205.24	607.89
601.16	1149.49667	560.43667
601.58	1272.28667	675.68667
602.01	1248.67667	576.00667
602.43	1283.33	603.87
602.85	1250.43667	610.15667
603.28	1275.29667	592.82667
603.7	1251.69333	663.38333
604.12	1218.29667	609.65667
604.55	1217.29667	638.77667
604.97	1167.57667	573.99667
605.39	1115.85333	605.88333
605.82	945.86333	431.37333
606.24	955.65333	450.21333
606.66	848.68667	389.18667
607.09	841.16333	338.72333
607.51	881.59	385.93
607.93	921.25333	390.95333
608.36	875.30667	440.66667
608.78	909.45333	419.82333
609.2	866.27	355.54
609.62	880.32667	406.51667
610.05	829.10667	371.86667
610.47	843.91667	382.16667
610.89	838.39667	370.60667
611.31	795.70667	324.15667
611.74	767.84333	328.68333
612.16	693.26667	274.44667
612.58	716.11667	294.27667
613	700.04333	324.91333
613.42	692.01333	274.69333
613.85	891.63	338.72
614.27	859.74333	350.52333
614.69	929.03667	359.56667
615.11	876.81333	369.86333
615.53	758.54333	297.54333
615.96	788.68	314.12
616.38	795.96333	310.85333

616.8	754.03	320.89
617.22	734.69	339.98
617.64	728.92333	237.78333
618.06	740.97667	286.74667
618.49	767.83333	281.97333
618.91	818.80667	276.45667
619.33	802.24333	309.59333
619.75	781.64667	310.84667
620.17	820.81667	395.97667
620.59	853.46	357.8
621.01	837.89	324.91
621.43	866.26667	362.32667
621.85	1147.49	611.16
622.28	915.98333	387.93333
622.7	895.14333	431.87333
623.12	917.48667	363.07667
623.54	913.97333	386.68333
623.96	927.53333	431.12333
624.38	917.48667	424.84667
624.8	854.97	381.91
625.22	927.53	364.08
625.64	1026.96667	400.99667
626.06	1018.18	504.44
626.48	1068.9	460.25
626.9	1100.53	463.26
627.32	1218.8	557.42
627.74	1203.23333	547.13333
628.16	1183.65	611.16
628.58	1272.03	615.18
629	1275.54667	605.88667
629.42	1213.77667	662.37667
629.84	1275.04333	576.00333
630.26	1236.62333	505.94333
630.68	1256.71667	586.29667
631.1	1236.12	588.31
631.52	1120.37	550.9
631.94	1229.6	538.84
632.36	1275.80333	621.95333
632.78	1248.17667	601.86667
633.2	1288.10333	635.01333
633.62	1372.97	684.48
634.04	1481.19	755.04
634.46	1541.95333	876.06333
634.87	1584.63667	855.46667
635.29	1598.71	877.07

635.71	1541.95667	840.65667
636.13	1658.46	934.57
636.55	1722.49	864.51
636.97	1720.23	832.87
637.39	1636.61333	857.73333
637.81	1579.11667	852.20667
638.23	1541.70667	825.34667
638.64	1533.42	765.83
639.06	1544.96667	804.49667
639.48	1524.88667	785.91667
639.9	1521.37	851.7
640.32	1532.16	845.18
640.74	1696.37667	871.53667
641.16	1713.95	953.9
641.57	1794.05333	1000.85333
641.99	1936.42667	1088.98667
642.41	1994.17	1095.52
642.83	1982.62	1148.74
643.25	2012.25	1155.02
643.66	1966.30667	1179.88667
644.08	2032.09	1195.95
644.5	1952.49333	1140.45333
644.92	1929.64	1070.15
645.33	1810.62667	995.57667
645.75	1675.78667	939.83667
646.17	1737.81	972.48
646.59	1690.35333	937.07333
647	1655.44333	879.57333
647.42	1718.98	906.94
647.84	1738.06333	958.42333
648.26	1782.00667	1029.47667
648.67	1938.42667	1138.44667
649.09	2054.68333	1166.57333
649.51	2088.83333	1197.71333
649.93	2319.34333	1337.82333
650.34	2222.41667	1295.88667
650.76	2143.82	1353.64
651.18	2246.26667	1341.57667
651.59	2252.54667	1347.10667
652.01	2298.25	1314.47
652.43	2179.47667	1292.86667
652.84	2069.25	1120.12
653.26	1844.02	1077.19
653.68	1817.15667	1034.49667
654.09	1792.3	1039.78

654.51	1889.46667	1168.58667
654.92	1929.14	1261.74
655.34	2036.1	1312.21
655.76	1973.33	1322.5
656.17	2011.75	1287.1
656.59	2177.72	1352.13
657.01	2173.70333	1340.58333
657.42	2242.25	1391.05
657.84	2313.56333	1448.04333
658.25	2231.45	1466.88
658.67	2187.76667	1368.19667
659.08	2162.65	1363.43
659.5	2194.04	1398.58
659.92	2091.85	1279.82
660.33	2093.6	1330.53
660.75	1976.1	1231.86
661.16	1798.57667	1095.76667
661.58	1732.54	992.07
661.99	1662.48	1002.61
662.41	1552.50333	919.75333
662.82	1624.57	974.49
663.24	1579.12333	910.21333
663.65	1648.41333	959.92333
664.07	1740.57333	1026.46333
664.48	1774.46667	1034.74667
664.9	1815.89667	1073.16667
665.31	1870.89	1199.72
665.73	1940.94667	1222.31667
666.14	1991.66	1254.96
666.56	2011.74667	1272.02667
666.97	1932.40667	1244.66667
667.39	1820.91667	1206.24667
667.8	1836.48333	1062.87333
668.22	1775.97667	1136.44667
668.63	1645.65333	1033.24333
669.04	1424.69667	857.47667
669.46	1346.36	746.75
669.87	1231.35667	658.86667
670.29	1201.22	721.39
670.7	1161.80333	704.56333
671.11	1095.76333	645.30333
671.53	1151.51333	631.75333
671.94	1103.80333	667.65333
672.36	1146.23	713.85
672.77	1237.38	718.38

673.18	1292.11333	742.97333
673.6	1300.65333	802.74333
674.01	1347.60667	809.01667
674.42	1285.33333	800.98333
674.84	1227.09	708.83
675.25	1191.17667	739.96667
675.66	1307.69333	815.04333
676.08	1280.57333	760.81333
676.49	1170.59667	757.04667
676.9	1108.32	628.48
677.32	941.84667	527.54667
677.73	792.19	429.11
678.14	696.03	389.44
678.56	667.9	307.84
678.97	680.70667	241.54667
679.38	591.32667	271.93667
679.79	521.52	228.5
680.21	546.12667	208.65667
680.62	468.53333	162.70333
681.03	538.84	230.75
681.44	499.92333	216.69333
681.86	553.90667	245.06667
682.27	515.24	241.8
682.68	592.57333	243.05333
683.09	528.04667	241.80667
683.51	513.23	220.21
683.92	468.54	226.73
684.33	494.9	216.94
684.74	441.67333	171.24333
685.15	397.97333	169.73333
685.57	396.22667	174.75667
685.98	358.30667	86.37667
686.39	327.17333	30.38333
686.8	272.18333	20.59333
687.21	252.09333	25.98883
687.62	200.36667	0
688.04	164.21	0
688.45	151.15667	0
688.86	122.28	0
689.27	103.45667	0
689.68	122.28	0
690.09	100.18	0
690.5	70.56333	0
690.91	116	0
691.33	101.69333	0

691.74	123.28333	0
692.15	415.05	0
692.56	461	0
692.97	466.27	0
693.38	442.93	0
693.79	505.45	0
694.2	497.92	45.08
694.61	788.68	120.67
695.02	1079.44	179.44
695.43	1288.11	388.11
695.84	1318.23	818.23
696.25	1124.64	224.64
696.66	885.85	120.45
697.07	741.98	90.23
697.48	707.32	0
697.89	667.4	0
698.3	680.21	0
698.71	580.78	0
699.12	528.8	0
699.53	541.61	0
699.94	467.03	0
700.35	525.78	0
700.76	525.03	0
701.17	479.08	0
701.58	465.52	0
701.99	433.13	0
702.4	485.86	0
702.81	494.9	0
703.22	468.54	0
703.63	513.73	0
704.04	447.45	98.23095
704.45	584.55	97.32095
704.85	759.3	120.028
705.26	1013.9	213.9
705.67	1144.98	444.98
706.08	1152.51	752.51
706.49	960.42	260.42
706.9	839.15	180.76
707.31	753.28	100.23
707.72	857.23	90.5898
708.12	677.95	80.394
708.53	652.34	76.3932
708.94	546.88	0
709.35	537.08	0
709.76	501.68	0

710.17	537.84	65.7
710.57	497.16	30.3
710.98	472.31	70.98
711.39	476.08	77.75
711.8	568.73	65.7
712.21	538.59	30.3
712.61	518.25	70.98
713.02	563.45	65.7
713.43	642.54	378.31
713.84	656.86	302.23
714.24	731.43	222.38
714.65	760.05	263.81
715.06	820.32	324.08
715.47	787.92	70.98
715.87	703.56	77.75
716.28	653.84	77.75
716.69	657.61	30.3
717.1	632.75	70.98
717.5	568.72	77.75
717.91	574	65.7
718.32	643.3	30.3
718.72	650.08	70.98
719.13	574.75	77.75
719.54	574.75	65.7
719.94	555.92	30.3
720.35	626.72	70.98
720.76	592.07	77.75
721.16	595.09	0
721.57	974.74	74.74
721.98	644.8	378.31
722.38	721.64	302.23
722.79	683.97	222.38
723.19	642.54	263.81
723.6	714.86	324.08
724.01	680.21	30.3
724.41	719.38	70.98
724.82	717.12	77.75
725.22	686.99	30.3
725.63	839.9	378.31
726.04	797.72	302.23
726.44	809.77	222.38
726.85	812.78	263.81
727.25	743.48	324.08
727.66	699.04	65.7
728.06	735.2	30.3

728.47	690	378.31
728.87	722.39	302.23
729.28	706.58	222.38
729.69	765.32	263.81
730.09	740.47	324.08
730.5	758.55	65.7
730.9	775.88	30.3
731.31	843.67	70.98
731.71	866.27	77.75
732.12	903.18	3.18
732.52	881.33	0
732.92	902.43	2.43
733.33	904.68	4.68
733.73	965.7	65.7
734.14	930.3	30.3
734.54	970.98	70.98
734.95	977.75	77.75
735.35	1014.67	114.67
735.76	1035	135
736.16	1148.75	248.75
736.56	1569.07	669.07
736.97	1984.88	1084.88
737.37	2200.32	400.32
737.78	2044.39	1144.39
738.18	1679.8	779.8
738.58	1463.62	563.62
738.99	1278.31	378.31
739.39	1202.23	302.23
739.79	1122.38	222.38
740.2	1163.81	263.81
740.6	1224.08	324.08
741.01	1231.61	331.61
741.41	1232.36	332.36
741.81	1199.97	299.97
742.21	1361.93	461.93
742.62	1490.74	590.74
743.02	1641.39	741.39
743.42	1657.2	757.2
743.83	1602.22	702.22
744.23	1540.45	640.45
744.63	1593.18	693.18
745.04	1863.6	963.6
745.44	2166.42	1266.42
745.84	2257.56	457.56
746.24	2180.73	1280.73

746.65	2020.29	1120.29
747.05	1853.81	953.81
747.45	1791.29	891.29
747.85	1768.69	868.69
748.26	2266.61	466.61
748.66	3413.84	1613.84
749.06	4269.57	2469.57
749.46	4982.92	3182.92
749.86	4695.92	2895.92
750.27	4320.03	2520.03
750.67	3798.77	1998.77
751.07	3066.59	1266.59
751.47	2592.78	792.78
751.87	2138.55	1238.55
752.28	1972.08	1072.08
752.68	1765.68	865.68
753.08	1539.69	639.69
753.48	1334.8	434.8
753.88	1267.01	367.01
754.28	1113.35	213.35
754.68	1107.32	207.32
755.08	997.34	97.34
755.49	906.19	6.19
755.89	995.83	95.83
756.29	1047.06	147.06
756.69	1035	135
757.09	1068.15	168.15
757.49	1075.68	175.68
757.89	1090.74	190.74
758.29	1056.84	156.84
758.69	995.08	95.08
759.09	1059.85	159.85
759.49	1049.31	149.31
759.89	1027.47	127.47
760.29	953.65	53.65
760.69	960.42	60.42
761.09	1113.34	213.34
761.5	1961.53	1061.53
761.9	3199.16	1399.16
762.3	3883.13	2083.13
762.7	3905.73	2105.73
763.1	3167.53	1367.53
763.49	2462.46	662.46
763.89	1972.83	1072.83
764.29	1647.41	747.41

764.69	1520.86	620.86
765.09	1369.46	469.46
765.49	1325.77	425.77
765.89	1108.07	208.07
766.29	971.73	71.73
766.69	815.04	0
767.09	702.81	0
767.49	741.22	0
767.89	669.66	0
768.29	685.48	0
768.69	760.06	0
769.09	725.41	0
769.48	793.95	0
769.88	806.76	0
770.28	1025.96	125.96
770.68	1440.26	540.26
771.08	1776.98	876.98
771.48	1846.28	946.28
771.88	1671.52	771.52
772.28	1401.85	501.85
772.67	1263.24	363.24
773.07	1212.02	312.02
773.47	1157.78	257.78
773.87	1128.41	228.41
774.27	1208.26	308.26
774.66	1743.08	843.08
775.06	4731.32	2931.32
775.46	11887.45	8587.45
775.86	18094.44	14794.44
776.26	20958.4	17658.4
776.65	17975.42	14675.42
777.05	12754.47	9454.47
777.45	9252.49	7452.49
777.85	7082.3	5282.3
778.24	5825.84	4025.84
778.64	5047.7	3247.7
779.04	4353.93	2553.93
779.44	3731.73	1931.73
779.83	3084.66	1284.66
780.23	2253.8	453.8
780.63	1424.44	524.44
781.02	853.46	0
781.42	612.42	0
781.82	425.6	0
782.21	422.59	0

782.61	314.11	0
783.01	248.58	0
783.4	177.78	0
783.8	248.58	0
784.2	210.16	0
784.59	193.59	0
784.99	176.27	0
785.39	143.87	0
785.78	140.86	0
786.18	120.53	0
786.57	216.19	0
786.97	153.67	0
787.37	155.93	0
787.76	182.29	0
788.16	209.41	0
788.55	164.96	0
788.95	122.03	0
789.35	155.93	0
789.74	144.63	0
790.14	117.51	0
790.53	181.53	0
790.93	152.16	0
791.32	92.66	65.7
791.72	143.12	30.3
792.11	182.29	70.98
792.51	335.96	77.75
792.9	752.52	90.765
793.3	1141.21	241.21
793.69	1339.33	839.33
794.09	1307.69	607.69
794.48	1076.43	476.43
794.88	836.89	393.09238
795.27	691.5	287.932
795.67	549.14	65.7
796.06	419.57	30.3
796.46	462.51	70.98
796.85	377.4	77.75
797.24	295.28	0
797.64	283.98	0
798.03	332.95	0
798.43	272.68	0
798.82	384.93	0
799.21	659.12	0
799.61	869.28	0
800	1037.27	137.27

800.4	1117.87	717.87
800.79	963.44	553.44
801.18	699.04	483.94982
801.58	570.23	290.349
801.97	457.99	180.39034
802.36	357.05	108.32309
802.76	336.71	65.7
803.15	373.62	30.3
803.54	274.19	70.98
803.94	183.8	77.75
804.33	195.85	0
804.72	142.37	0
805.12	112.24	0
805.51	117.51	0
805.9	112.24	0
806.29	48.21	0
806.69	157.44	0
807.08	140.86	0
807.47	135.59	0
807.86	231.25	0
808.26	528.04	0
808.65	919.75	19.75
809.04	1457.59	557.59
809.43	2451.16	651.16
809.83	3414.6	1614.6
810.22	3978.81	2178.81
810.61	3819.11	2019.11
811	3043.23	1243.23
811.39	2282.43	482.43
811.79	1843.26	943.26
812.18	1468.13	568.13
812.57	1292.62	392.62
812.96	1115.6	215.6
813.35	983.03	83.03
813.74	870.03	0
814.14	711.85	0
814.53	525.79	0
814.92	1052.33	152.33
815.31	338.98	0
815.7	213.18	0
816.09	267.41	0
816.48	298.3	0
816.87	466.28	0
817.26	564.96	0
817.66	572.49	0

818.05	552.15	0
818.44	455.73	0
818.83	439.92	0
819.22	552.9	0
819.61	725.41	0
820	1035.75	135.75
820.39	1167.57	267.57
820.78	1331.79	731.79
821.17	1235.38	635.38
821.56	1053.83	453.83
821.95	934.06	234.06
822.34	806	389.0984
822.73	753.27	480.82388
823.12	781.14	490.293
823.51	658.37	378.40921
823.9	613.16	389.2092
824.29	584.54	290.3249
824.68	829.36	304.342
825.07	844.42	235.38
825.46	766.84	153.83
825.85	570.98	234.06
826.24	443.68	0
826.63	351.78	0
827.02	261.39	0
827.4	235.78	0
827.79	253.85	0
828.18	180.03	0
828.57	191.33	0
828.96	134.08	0
829.35	72.31	0
829.74	51.23	0
830.13	24.86	0
830.52	75.32	0
830.9	0	0
831.29	58.01	0
831.68	31.64	0
832.07	30.89	0
832.46	28.62	0
832.85	33.9	0
833.23	44.45	0
833.62	55.75	0
834.01	40.67	0
834.4	70.05	0
834.79	74.58	0
835.17	35.4	0

835.56	61.01	0
835.95	106.21	0
836.34	102.45	0
836.72	58.75	0
837.11	51.23	0
837.5	72.31	0
837.89	94.16	0
838.27	172.5	0
838.66	553.65	0
839.05	873.8	0
839.43	1035.75	135.75
839.82	1178.13	278.13
840.21	1244.41	344.41
840.6	1537.44	637.44
840.98	1640.63	740.63
841.37	1685.83	785.83
841.76	1416.91	516.91
842.14	1610.5	710.5
842.53	2930.24	1130.24
842.91	4222.11	2422.11
843.3	4787.07	2987.07
843.69	4467.68	2667.68
844.07	3223.27	1423.27
844.46	2354.74	554.74
844.85	1925.38	1025.38
845.23	1627.83	727.83
845.62	1464.36	564.36
846	1250.44	350.44
846.39	1086.23	186.23
846.78	1020.68	120.68
847.16	852.71	0
847.55	647.07	0
847.93	682.47	0
848.32	419.57	0
848.7	316.37	0
849.09	260.63	0
849.47	181.54	0
849.86	244.82	0
850.24	392.45	0
850.63	443.68	0
851.01	440.67	0
851.4	372.11	0
851.78	285.49	0
852.17	189.83	0
852.55	206.4	0

852.94	149.15	0
853.32	164.96	0
853.71	155.93	0
854.09	139.35	0
854.48	0	0
854.86	137.1	0
855.24	182.29	0
855.63	145.38	0
856.01	152.91	0
856.4	188.32	0
856.78	161.95	0
857.16	140.11	0
857.55	149.9	0
857.93	251.59	0
858.32	247.08	0
858.7	150.65	0
859.08	128.06	0
859.47	143.87	0
859.85	119.01	0
860.23	186.06	0
860.62	209.41	0
861	268.92	0
861.38	345.76	0
861.77	345.75	0
862.15	326.92	0
862.53	359.32	0
862.91	296.79	0
863.3	320.9	0
863.68	369.11	0
864.06	334.46	0
864.45	385.68	0
864.83	340.48	65.7
865.21	434.64	30.3
865.59	610.16	70.98
865.98	855.73	77.75
866.36	1114.1	214.1
866.74	1376.24	776.24
867.12	1181.14	681.14
867.5	1035.75	535.75
867.89	885.86	510.302
868.27	840.65	480.23812
868.65	894.14	289.0329
869.03	781.9	200.398
869.41	791.7	160.39823
869.79	726.91	65.7

870.18	806.75	30.3
870.56	604.88	70.98
870.94	564.21	77.75
871.32	473.81	65.7
871.7	463.27	30.3
872.08	409.78	70.98
872.46	302.82	77.75
872.84	289.26	0
873.23	213.17	0
873.61	200.38	0
873.99	238.79	0
874.37	153.67	0
874.75	109.98	0
875.13	120.52	0
875.51	75.33	0
875.89	61.02	0
876.27	51.22	0
876.65	67.05	0
877.03	51.22	0
877.41	19.59	0
877.79	6.78	0
878.17	51.22	0
878.55	43.69	0
878.93	103.95	0
879.31	58.76	0
879.69	67.05	0
880.07	127.3	0
880.45	164.96	0
880.83	114.5	0
881.21	161.2	0
881.59	91.9	0
881.97	149.9	0
882.35	131.07	0
882.73	128.81	0
883.11	93.41	0
883.49	116.76	0
883.86	74.57	0
884.24	131.07	0
884.62	94.92	0
885	111.49	0
885.38	102.45	0
885.76	103.95	0
886.14	79.1	0
886.52	96.42	0
886.89	95.66	0

887.27	100.94	0
887.65	85.88	0
888.03	54.99	0
888.41	27.12	0
888.79	50.47	0
889.16	0	0
889.54	65.53	0
889.92	0	0
890.3	0	0
890.67	16.57	0
891.05	0	0
891.43	0	0
891.81	0	0
892.19	0	0
892.56	0	0
892.94	0	0
893.32	0	0
893.69	0	0
894.07	0	0
894.45	0	0
894.83	0	0
895.2	0	0
895.58	0	0
895.96	0	0
896.33	0	0
896.71	0	0
897.09	0	0
897.46	0	0
897.84	0	0
898.21	0	0
898.59	0	0
898.97	0	0
899.34	0	0
899.72	0	0

Data of Fig 3(b).

Wavelength (nm)	Intensity (a.u.)	
	Fixed ground electrode config.	Floating water ground electrode config.
304.01865	5.00001	0
304.05484	20.44445	0
304.091	81	18.66667
304.1272	0	0
304.16336	0	0
304.19952	0	33.66667
304.23569	26.55556	25.22222
304.27185	0	42.33333
304.30801	34.77778	30.77778
304.34418	63.55556	56.55556
304.38034	0	15.33333
304.4165	79.22222	117.55556
304.45264	10.66667	0
304.4888	58.22221	66.55555
304.52493	0	25.66667
304.56107	30.55555	40.22222
304.59723	0	21.55556
304.63336	52.88889	45.88889
304.66949	29.22222	0
304.70563	0	15.77778
304.74176	31.22223	76.88888
304.77789	44.44445	55.11112
304.814	69	14.33333
304.85013	16.44444	52.44444
304.88626	0	0.33333
304.92236	0	0
304.95847	77.44444	5.44444
304.9946	25.11111	50.11111
305.0307	118.88889	125.22223
305.0668	75.55556	77.22223
305.10291	0	0
305.13901	73.33334	72.66669
305.17511	26.33334	0
305.21121	91.22222	162.55557
305.24728	82.88889	86.22222
305.28339	77.33333	95.66667
305.31946	36.22222	2.22222
305.35556	56	16
305.39163	51.22222	84.88889
305.4277	0	0

305.46381	26.88889	22.55556
305.49988	12.22223	53.55556
305.53595	63	41.33333
305.57202	15.1111	57.1111
305.60806	109.22221	63.55556
305.64413	14.66667	42.66667
305.68021	18.77778	17.11111
305.71625	60.33334	96.99999
305.75232	77	146.00002
305.78836	9.55556	10.88889
305.8244	1	6.66666
305.86044	0	54.44445
305.89651	55.44445	52.11111
305.93256	24.11111	68.77778
305.96857	18.11111	7.77777
306.00461	2.55555	0
306.04065	50.00001	91.66666
306.07669	102	150.33335
306.1127	57.44444	21.11111
306.14874	0	3.33334
306.18475	61.44445	39.11111
306.22076	1	51.33333
306.25681	55.33334	95.33334
306.29282	175.33332	230.66666
306.32883	249.88888	330.55554
306.36484	197.8889	255.22221
306.40085	134.33333	111.66667
306.43683	114.88889	116.88889
306.47284	101.66667	81
306.50885	51.22222	109.88889
306.54483	118.77777	116.77777
306.58084	67.33333	80.66667
306.61682	78.66667	62
306.6528	150.44444	186.44446
306.68878	185.44444	213.77777
306.72476	286.55556	261.88888
306.76074	166.33333	214.33334
306.79672	106.55556	139.88889
306.8327	132.33333	129
306.86868	98.22222	163.88889
306.90463	183.77778	219.44444
306.94061	168.22222	229.22223
306.97656	169.66667	164.66667
307.01254	111.66667	115.33333
307.04849	51.66667	108.99999

307.08444	115.33334	179.33334
307.12039	187.11112	184.44444
307.15634	52.88889	48.55555
307.19229	112.55556	148.22223
307.22824	50.66666	134.33332
307.26419	16.66667	17.66667
307.30011	88.1111	145.11112
307.33606	73	80
307.37198	98.66667	141
307.40793	101	58
307.44385	158.44446	200.11111
307.47977	123.66667	151.66667
307.51569	19.55556	58.55556
307.55164	36.55556	51.55556
307.58752	31.00001	67
307.62344	89.77778	112.77778
307.65936	101.11111	177.77778
307.69528	172.33333	136.99999
307.73117	97.33332	207.33332
307.76709	91.66667	94.66667
307.80298	164.66668	211.66668
307.8389	84.55556	59.22222
307.87479	119.33332	234.66666
307.91068	73.66667	59.00001
307.94656	125.55555	170.88889
307.98245	182.33333	160.33333
308.01834	131.88889	165.22222
308.05423	69.55556	99.22222
308.09009	171.44443	225.11112
308.12598	217	244
308.16183	199.66667	207.33332
308.19772	170.44445	104.11111
308.23358	112.44445	139.44445
308.26947	146.11111	188.77778
308.30533	254.22222	258.55556
308.34119	155.66667	145.99999
308.37704	47.22222	145.55556
308.4129	185.55556	157.22222
308.44873	186.22221	256.22221
308.48459	263.1111	193.44445
308.52045	164.33333	137.66667
308.55627	104.11111	129.44445
308.59213	249.55557	224.22222
308.62796	123.88889	136.22222
308.66379	202.88889	162.22222

308.69965	306.99999	304.33333
308.73547	216.00001	224.33332
308.7713	108.77778	53.11111
308.80713	201.88889	194.22222
308.84296	342.66669	229.33334
308.87875	272.77779	261.44444
308.91458	412.66667	518
308.95041	601.11109	702.11116
308.98621	436.77778	500.44447
309.022	435.77778	458.11109
309.05783	352	345.33334
309.09363	306.11111	311.11111
309.12943	348.55556	383.55556
309.16522	308.99999	339.66668
309.20102	411.55556	388.55556
309.23682	497.99998	548.66667
309.27261	286.11111	223.77778
309.30838	184.88889	146.88889
309.34418	204.33333	144.66666
309.37994	255.22222	269.88889
309.41574	308.88888	300.22222
309.45151	342.8889	355.22221
309.48727	394.33331	426
309.52307	367.55556	402.55556
309.55884	377.44447	368.11109
309.5946	399.22223	375.22222
309.63034	357.11111	330.44445
309.66611	354.55558	349.2222
309.70187	178.55555	131.22222
309.73761	152.11112	112.77778
309.77338	312.22222	238.22222
309.80911	438.33335	448.99998
309.84488	410.8889	402.22221
309.88062	425.44444	435.77778
309.91635	364.55557	310.88888
309.95209	303.99999	275.66667
309.98782	214.33333	138.66666
310.02356	315.11111	293.11111
310.0593	302.00001	228
310.09503	287	281
310.13074	462.33331	417.66669
310.16647	588.33331	549.66669
310.20218	503.99999	400.33333
310.23792	484.66667	369.00001
310.27362	411.77777	313.77777

310.30933	489.88889	354.55557
310.34503	324.55556	145.88889
310.38074	454.11111	385.44443
310.41644	302	142.66667
310.45215	317.44444	230.44445
310.48785	374.88889	255.22222
310.52353	493.1111	415.44444
310.55923	624.33331	468
310.59491	607.22222	545.55554
310.63062	528.55556	491.55556
310.66629	383.33333	349.33333
310.70197	435.33334	303.33334
310.73764	374.77777	266.77778
310.77332	448.22223	309.88889
310.80899	312	216.66667
310.84467	389.55555	196.88889
310.88034	480.55555	395.8889
310.91599	552.55554	410.88888
310.95166	494.00001	369.00001
310.9873	532.33331	372.33334
311.02298	382.66666	278.66667
311.05862	488.11109	293.11112
311.09427	521.33334	304
311.12991	560.11109	376.11112
311.16556	625.33331	558.33331
311.2012	764.55552	621.55558
311.23685	566.33333	359.66668
311.27249	606.99999	359
311.30814	596.11109	440.11112
311.34375	535.11111	337.44446
311.37939	632.8889	359.22223
311.41501	692.33333	406.66668
311.45062	791.77776	509.77776
311.48627	679.2222	365.22223
311.52188	839.88893	491.22224
311.5575	779.77776	313.77777
311.59311	830.77776	534.44444
311.62872	731.66669	408
311.66431	684.66669	545.33331
311.69992	714.7778	639.44442
311.73553	726.77776	650.11113
311.77112	516.88889	357.22222
311.80673	449.11111	351.77777
311.84232	340.11111	179.11111
311.8779	446.88887	247.22221

311.91348	589.22222	376.88889
311.94907	509.77779	346.77778
311.98465	469.66666	268.33333
312.02023	402.33333	274.66666
312.05582	401.66667	201.66667
312.0914	503.22224	311.8889
312.12695	611.66669	393
312.16254	689.55556	577.55556
312.19809	690.88887	527.55556
312.23364	802.33333	619.00002
312.26923	635.22223	453.88889
312.30478	605.88887	406.55556
312.34033	734.22224	552.55555
312.37589	723.22224	451.22221
312.41144	648.66667	384.66667
312.44699	677.88889	489.55558
312.48251	757.11109	393.77778
312.51807	634.55554	374.55557
312.55359	595.55556	350.22221
312.58914	583.99999	347.00001
312.62466	852	573.33331
312.66019	802.77778	424.44443
312.69574	858.99998	458.33332
312.73126	775.77776	526.1111
312.76678	982.11111	679.44442
312.80228	948.55552	655.55558
312.8378	984.33337	633.66669
312.87332	854.77778	499.11109
312.90884	808.22222	432.8889
312.94434	909.11113	532.1111
312.97986	926.33331	607.33334
313.01535	1044.77778	697.11109
313.05084	972.11109	516.44443
313.08633	1011.11116	602.11109
313.12183	880.77778	470.11109
313.15732	862.11113	465.77779
313.19281	935.2222	489.55555
313.2283	973.77778	505.44446
313.26379	1010.99998	580.00001
313.29926	1198.22226	727.88889
313.33475	1343.22218	693.88887
313.37021	1528.11111	847.44442
313.4057	1766.77782	988.44444
313.44116	2159.55564	1108.88889
313.47662	2321.66658	1167.66671

313.51208	2117.66659	1134.99996
313.54755	1449.33333	774.66664
313.58301	998.77774	606.11111
313.61847	724.99998	454.33332
313.65393	711.88887	465.22221
313.68936	609.00002	362.99999
313.72482	480.44444	317.77777
313.76025	595.11109	358.11112
313.79572	592	290.66667
313.83115	571.66665	272.66666
313.86658	563.77777	328.77779
313.90201	773.11113	431.77776
313.93744	642.55554	439.55557
313.97287	744.55558	511.55558
314.0083	759.11111	553.7778
314.04373	811.11113	452.77779
314.07913	708.66665	461.00002
314.11456	629.22223	338.55556
314.14996	614.55554	290.55555
314.18539	730.55556	466.8889
314.22079	842.44445	566.77776
314.2562	705.55558	419.22223
314.2916	946.11111	677.44442
314.327	776.88887	455.22221
314.3624	876.55558	622.2222
314.3978	831.77776	465.44445
314.4332	854.44447	454.77778
314.46857	956.22222	583.22222
314.50397	965.11111	596.11111
314.53934	864.33333	537.00002
314.57474	929.11109	597.44447
314.61011	961.2222	535.2222
314.64548	947	691.33331
314.68085	1157.11113	761.44445
314.71622	1155.88889	791.55558
314.75159	1119.88887	614.22221
314.78696	992.7778	567.11111
314.82233	1178.77774	601.11111
314.85767	1056.22224	504.55556
314.89304	1023.22224	495.55556
314.92838	1297.33329	796.66667
314.96375	1336.11107	839.77776
314.99908	1371.55555	893.55555
315.03442	1300.5556	739.22222
315.06976	1555.22226	1012.55551

315.1051	1368.22222	661.88891
315.14044	1467.11111	778.11111
315.17578	1506.5556	881.22222
315.21112	1514.33333	878.66671
315.24643	1496.55551	832.88889
315.28177	1547.88893	857.55556
315.31708	1546.11111	855.44449
315.35242	1606.33337	828.66662
315.38773	1649.44449	944.11111
315.42303	1678.77774	933.44442
315.45834	1643.99996	893.66665
315.49365	1721.88885	931.22222
315.52896	1696.44444	854.77782
315.56427	1729.77774	976.11111
315.59958	1721.5556	866.55554
315.63486	1957.55551	1099.88889
315.67017	2283.99992	1229.66667
315.70544	2690.66658	1518.66671
315.74075	3443.44436	1811.77774
315.77603	4209.55539	2278.88889
315.81131	4963.66667	2699.99992
315.84659	4190.33333	2222.33333
315.88187	2391.55556	1162.22218
315.91714	877.11113	458.44444
315.95242	375.1111	226.77777
315.9877	243.77778	255.44443
316.02295	275.66667	173.33332
316.05823	174.55556	183.22221
316.09348	134	106.66666
316.12875	145.44444	220.44444
316.164	243.55556	234.8889
316.19925	89.8889	67.55556
316.2345	115.66667	177.33333
316.26974	164.99999	165.99999
316.30499	121.00001	136.33334
316.34024	129.66669	196
316.37546	63.88889	87.8889
316.41071	126.77778	192.44445
316.44595	132.22222	162.55555
316.48117	218.33334	245.66668
316.51639	96.55556	85.55556
316.55164	76.11111	108.77778
316.58685	126.33334	125.00001
316.62207	97.44445	164.11112
316.65729	58.22222	44.55556

316.6925	78.66667	99.99999
316.72772	102.55555	28.55556
316.76291	66.33332	61.66667
316.79813	122.11111	66.77778
316.83331	222.44445	299.11113
316.86853	159.11112	271.77777
316.90372	104.11111	193.77778
316.93893	149	133.33333
316.97412	89.55556	141.55555
317.00931	89.33333	35.66667
317.04449	60.88889	28.88889
317.07968	38.44445	0
317.11484	106.55555	118.22222
317.15002	49.55556	31.55556
317.18521	31.44443	58.77778
317.22037	49.22222	58.22222
317.25555	137.22223	140.22223
317.29071	53.11111	84.77778
317.32587	94.22223	126.22223
317.36102	182	199
317.39618	262.33332	269.66667
317.43134	195.66667	220.99999
317.46649	169.33332	191.66667
317.50165	128.44444	112.11111
317.5368	5.33333	0
317.57193	0	0
317.60709	141.33333	227.66665
317.64221	125.66667	126.00001
317.67737	31.22222	101.22223
317.71249	68.11111	68.11111
317.74762	168.55555	168.22222
317.78275	127.22222	95.55556
317.81787	99.33334	115.33334
317.853	95.55555	37.88889
317.88812	114.66667	55
317.92322	137.33332	130.33332
317.95834	118.88889	152.55557
317.99347	136.22223	89.22222
318.02856	99.11111	108.44445
318.06366	178.55557	225.22226
318.09875	97.11111	170.44444
318.13388	156.77777	182.44444
318.16898	99.77778	92.44444
318.20407	116.77778	147.11112
318.23914	118.33334	167.33333

318.27423	140.33333	165.66668
318.30933	118.22222	123.22222
318.34439	138.33333	122.66667
318.37949	22.55556	0
318.41455	114.11111	124.77778
318.44965	26.55555	31.22222
318.48471	122.77778	42.11111
318.51978	141.88889	118.88889
318.55484	99.33333	92
318.5899	217.55553	265.22222
318.62497	90.77778	73.77778
318.66003	57.77778	114.11111
318.69507	132	70
318.73013	61.66667	79.99999
318.76517	105.11108	137.44446
318.80023	144.55553	212.55553
318.83527	23.88889	15.22222
318.8703	17.44445	38.77778
318.90533	69.88889	43.88889
318.94037	47.55555	40.55555
318.9754	29.88889	0
319.01044	102.77777	125.77777
319.04547	129.66667	123.33334
319.08047	68.22222	75.88889
319.11551	112	85
319.15054	89.44444	150.77777
319.18555	158.77777	145.44445
319.22055	92.77778	148.11112
319.25555	123.55554	103.55554
319.29056	132.77777	184.11111
319.32556	48.33333	43.33333
319.36057	26.66667	8.66667
319.39557	85.55555	51.22222
319.43057	143.22223	163.88889
319.46558	185.77778	189.11112
319.50055	99.66667	145.33333
319.53555	94.88889	118.55555
319.57053	46.22223	32.55555
319.6055	62.66667	61.33334
319.64047	37.77778	0
319.67545	28.44445	13.44445
319.71042	70.66667	140.66666
319.74539	78.66667	50.33333
319.78036	165.22222	159.88888
319.81534	122.55556	75.55556

319.85028	50.44444	51.11111
319.88525	107	138
319.9202	50.11112	92.77778
319.95517	86.11111	108.11111
319.99011	116.88889	119.55555
320.02505	167.66669	212.66669
320.06	41.44445	87.77778
320.09494	154.44445	172.44445
320.12988	73	26.66667
320.16483	101.66666	107.66667
320.19977	73.44445	82.77778
320.23468	65.77777	47.44444
320.26962	31.11111	16.11111
320.30453	80.55555	36.55555
320.33945	63.44445	65.44445
320.37439	88.11111	66.77778
320.4093	58.33333	80.33333
320.44421	84.88889	42.55556
320.47913	0.77778	39.11111
320.51404	49.77778	16.44444
320.54892	84.88889	106.88889
320.58383	95	57
320.61874	110.33333	73.66667
320.65363	86.66666	61.33333
320.68854	24.55556	13.88889
320.72342	140.77778	64.44444
320.7583	141.11111	172.44446
320.79318	32	10.66667
320.82806	12.77778	48.11111
320.86295	52.44444	82.11111
320.89783	43	60
320.93271	112.00001	90.00001
320.96756	173.77777	220.77777
321.00244	143.22224	96.22224
321.03729	182.88891	145.55553
321.07217	50.22222	65.88889
321.10703	58.88889	60.88889
321.14188	82.77778	72.77778
321.17673	125.44444	60.44445
321.21158	127.66666	100.33333
321.24643	94.11111	149.44444
321.28128	152.33333	187.33333
321.31613	172.11111	155.44445
321.35095	104.88889	75.55555
321.3858	161.33332	116.33332

321.42062	123.11111	94.77777
321.45547	83	71.33334
321.4903	66	25.99999
321.52512	55.44445	64.44445
321.55994	0	0
321.59476	62.55556	61.22222
321.62958	112.22222	69.55556
321.6644	76.88889	127.55555
321.69922	105.44444	121.77778
321.73401	52.1111	91.11112
321.76883	90.66667	87.00001
321.80362	85.44444	81.77778
321.83841	75.55555	83.88888
321.87323	71.77778	111.11111
321.90802	177	171.33334
321.94281	74.88889	115.88889
321.9776	113.66666	63.33333
322.01239	41.22222	52.22222
322.04715	102.66667	49.00001
322.08194	30.1111	70.44445
322.11673	107	110
322.15149	169.33333	192.99999
322.18628	86.33333	41.66667
322.22104	123.11112	129.11112
322.2558	125.77779	125.1111
322.29056	60.22222	63.22222
322.32532	146.66667	83
322.36008	70.66667	84.33333
322.39484	93.88889	37.22222
322.4296	71.55555	54.22222
322.46432	215.99998	242.66667
322.49908	88.00001	90.00001
322.53381	32	5.33333
322.56857	114.11112	126.44443
322.6033	37.22222	30.88889
322.63803	71.66667	140.33333
322.67276	68.88889	99.88889
322.70749	81.22222	99.55556
322.74222	85.66667	58.33334
322.77695	144.33333	188.66665
322.81168	126.22222	67.22222
322.84637	118.11111	62.44445
322.8811	103.55555	102.55555
322.9158	71.00001	42.66666
322.95053	68.88889	74.22222

322.98523	14.66666	36.66666
323.01993	81.55555	75.55555
323.05463	86.88889	125.55555
323.08932	83.77778	27.11111
323.12402	14.55556	57.88889
323.15872	92.77777	76.11111
323.19342	94.1111	97.44445
323.22809	0	0
323.26279	49.33334	54.33334
323.29745	45.33333	70.33333
323.33212	201.11109	226.44444
323.36682	136.55557	83.8889
323.40149	80.22223	99.88889
323.43616	67.88889	6.22222
323.47083	236.77777	255.77777
323.50549	206.44442	236.77779
323.54013	81.11111	96.11111
323.5748	130.55555	80.88889
323.60947	78.77778	139.44444
323.6441	148.33332	130.00001
323.67874	150	148.33333
323.71341	71.88889	17.22222
323.74805	120.11111	101.44445
323.78268	176.11112	130.77777
323.81732	118.77778	113.11111
323.85196	86	73.66667
323.8866	174.88889	159.22223
323.9212	109.88888	43.55555
323.95584	200.66666	225
323.99048	142.77778	146.44444
324.02509	102.22222	84.88889
324.05969	115.88888	27.22222
324.09433	147.44444	99.11112
324.12894	222.11111	140.11111
324.16354	120.11112	109.11112
324.19815	163.44443	80.1111
324.23276	186.33333	143.66666
324.26736	204.66668	86.33333
324.30194	149.77779	121.44445
324.33655	163.55557	107.22222
324.37112	176.66666	211.33333
324.40573	234.33332	175.99999
324.44031	215.77779	144.1111
324.47488	106.00001	87.00001
324.50946	143	67.66666

324.54404	174.11111	150.11111
324.57861	256.11111	176.11111
324.61319	173.44445	26.77778
324.64777	226.44444	161.11112
324.68234	238.44445	133.44445
324.71689	251.22221	164.22221
324.75146	176.77778	59.11111
324.78601	132.88889	100.88889
324.82056	126.11111	50.11111
324.85513	250.77779	138.44444
324.88968	128.77777	64.44444
324.92422	135	71.66667
324.95874	243.33333	110.66667
324.99329	248.11111	197.11111
325.02783	240.33333	99.33333
325.06238	195.33332	152
325.09689	196.77777	164.77778
325.13144	145.22223	88.55556
325.16595	259.44445	137.77778
325.20047	179	120.66667
325.23499	264.22222	164.22222
325.2695	213.66668	150.99999
325.30402	280.11112	197.44443
325.33853	264	218
325.37305	263.11112	141.44444
325.40756	241.33334	145.66666
325.44205	212.11111	156.11111
325.47656	201.44445	136.11111
325.51105	227.88889	90.55555
325.54553	181.88889	141.22222
325.58005	261.55556	174.22222
325.61453	280.77779	253.1111
325.64902	231.88889	105.88889
325.6835	201.22224	146.8889
325.71799	277.22222	156.55557
325.75244	230.11111	101.77778
325.78693	246.66667	94
325.82141	232.33334	128.66666
325.85587	322.44444	153.11112
325.89032	338.55555	199.22222
325.9248	263.22222	178.55556
325.95926	187.44444	46.77778
325.99371	187.44445	93.11111
326.02817	264.77778	138.11112
326.06262	212.22222	54.88889

326.09708	360.11109	250.44447
326.1315	336.1111	195.44444
326.16595	413.33333	207.33333
326.20038	338.77779	148.44444
326.23483	261.55556	102.22223
326.26926	371.44443	215.44446
326.30368	286.11112	134.44444
326.3381	396.55557	152.88889
326.37253	422.77777	276.77777
326.40695	458.88887	278.55555
326.44138	444.66667	299.33332
326.4758	611.77778	355.77778
326.51022	479.44446	241.44446
326.54462	453.88887	181.8889
326.57904	545.22222	201.22222
326.61343	613.66665	275.66666
326.64783	840.44441	447.44447
326.68225	654.66665	286.66666
326.71664	676.44442	368.77777
326.75104	498.22221	258.22223
326.78543	393.66669	239.33334
326.81979	403.88889	219.55557
326.85419	199.22223	84.88889
326.88858	235.33332	122.99999
326.92294	227.66666	157.33333
326.95734	276.44444	141.44445
326.9917	247.77777	128.77778
327.02606	436.33333	310.66667
327.06042	291.00001	117.33333
327.09479	199.44444	49.44444
327.12915	221.44444	78.44445
327.16351	299.44442	157.11111
327.19788	227.11112	183.77778
327.23224	284.77777	99.77778
327.26657	385.55555	174.22222
327.30093	364.55556	174.22222
327.33527	291.11111	197.44446
327.3696	302.22222	116.55555
327.40396	227.99999	179.33333
327.43829	371	233.33334
327.47263	403.77777	242.44446
327.50696	393.11111	205.11111
327.54126	350.66666	224
327.57559	311.8889	108.88889
327.60992	410.44445	280.44444

327.64423	276.33333	114.66667
327.67856	237.00001	121.33334
327.71286	415	205.33334
327.74716	381.22222	254.22222
327.78146	404.8889	167.22223
327.81577	352.66667	251.66667
327.85007	478.55553	261.88888
327.88437	319.44444	107.77777
327.91867	431.99998	259.66667
327.95297	384.22222	215.88889
327.98724	350.44443	206.77778
328.02155	459.55555	251.55555
328.05582	537.2222	265.88889
328.09012	586.22222	319.22222
328.12439	441.11111	194.44445
328.15866	430.44447	183.77778
328.19293	508.55553	237.55556
328.2272	466.1111	288.77779
328.26147	493.1111	254.77777
328.29572	758.2222	360.2222
328.32999	843.44444	416.77779
328.36423	930.55555	492.22221
328.3985	807.22224	404.55556
328.43274	694.55554	436.88891
328.46698	512.33333	245.66668
328.50125	293.22222	138.88889
328.53549	285.22223	187.55556
328.56973	279.33334	145.66667
328.60397	323.33334	147.33334
328.63818	269.55555	198.55555
328.67242	155.33333	79
328.70667	259.55557	110.22222
328.74088	186.33334	96
328.77509	138.77778	50.77779
328.80933	315	187.66666
328.84354	307.22221	162.22221
328.87775	288.8889	162.55556
328.91196	341.1111	214.77779
328.94617	216.55555	138.22222
328.98038	151.11111	88.11111
329.01456	176.00001	99.33333
329.04877	237.33334	179.33333
329.08298	463.33332	261.66667
329.11716	354.2222	159.22223
329.15134	311.44446	225.77778

329.18555	229.88888	116.55556
329.21973	154	69.33333
329.25391	155.8889	63.22221
329.28809	270.88888	129.88889
329.32227	361.33334	185.33333
329.35641	486.77778	242.11112
329.39059	302.66668	187.00001
329.42477	139.99999	62.99999
329.45892	247.66667	206.66667
329.4931	168.33333	94
329.52725	230.44443	117.77777
329.5614	318.77778	115.11111
329.59555	437	256.66666
329.6297	307.22223	149.88889
329.66385	317.77777	188.44445
329.698	261.99998	170.99998
329.73215	313.22222	159.55556
329.76627	327.55555	175.55555
329.80042	418.66667	268.66667
329.83453	520.44444	240.77775
329.86868	359.88889	186.55556
329.9028	242.11112	132.11111
329.93692	321.11111	252.44446
329.97104	182.66667	98
330.00516	424.55555	241.88889
330.03928	430.77777	177.44444
330.07339	490.11113	243.1111
330.10748	434.22224	204.55556
330.1416	301.66667	176
330.17569	315.55555	206.22222
330.20981	474.11106	336.11112
330.2439	352.33332	142.66667
330.27798	460.77777	223.44444
330.31207	585.99999	227.66667
330.34616	480.77776	317.77776
330.38025	357.8889	226.8889
330.41434	228.44444	183.77778
330.44843	329.8889	182.88888
330.48248	572.33331	315
330.51657	682.44443	364.7778
330.55063	612.77778	303.44445
330.58469	567.88889	378.22223
330.61877	368.88888	111.22222
330.65283	331.66666	137.33333
330.68689	527	338.33334

330.72095	595.44445	259.11111
330.755	848.55557	443.55557
330.78903	846.55554	395.55557
330.82309	584.22223	215.55556
330.85715	594.33335	293.00001
330.89117	496.11111	228.11111
330.9252	596.77776	268.77779
330.95926	681.55558	351.55555
330.99329	594.66668	209
331.02731	515.7778	310.11111
331.06134	249.22221	122.55556
331.09537	174.11112	202.44444
331.12936	319.33333	191.33333
331.16339	534.11113	280.1111
331.19742	730	325
331.23141	530.77778	252.77778
331.26544	338.44444	163.11111
331.29944	227.22223	113.22223
331.33344	244.66667	108.66667
331.36743	432.55556	238.88888
331.40143	753.7778	401.77777
331.43542	752.11109	453.44443
331.46942	455.88891	310.22222
331.50342	266.55556	135.55556
331.53738	337.55554	194.22222
331.57138	552.00002	391.33333
331.60535	834.2222	434.88889
331.63934	773.88891	357.88889
331.67331	555.66666	267.33333
331.70728	252.66668	99.00001
331.74124	334.77776	214.77779
331.77521	527.33332	295.33334
331.80917	749.00002	324
331.84314	943.55552	523.22221
331.87708	704.44444	343.77779
331.91104	447.44445	242.44445
331.94498	379.22223	168.88889
331.97894	653.55551	386.55557
332.01288	1005.6667	565.33333
332.04681	888	471.33334
332.08075	647.22221	326.22222
332.11469	470.8889	304.22221
332.14862	406.33333	154.00001
332.18256	700.44442	454.11111
332.21649	1088.00001	565.66666

332.2504	1179.55556	654.88894
332.28433	808.11107	454.44445
332.31824	439.77778	342.44443
332.35217	723.66665	407.66665
332.38608	810.66665	431.00002
332.41998	1065	489
332.45389	1021.00002	473.66668
332.48779	740.44442	363.44444
332.5217	477.77776	241.77776
332.5556	787.2222	387.55557
332.58948	1086.22222	589.55556
332.62338	1220.44444	561.44444
332.65726	1040.55551	583.55558
332.69116	658.33335	339.33332
332.72504	777.44446	437.44446
332.75891	900.88889	470.55558
332.79279	1342.44449	748.44442
332.82666	1383.44449	694.44442
332.86053	924.66667	482.66667
332.89441	667.88891	319.22222
332.92825	810.88889	489.88889
332.96213	1339.77782	636.44445
332.99597	1607.99996	806.00002
333.02985	1325.88893	632.88887
333.06369	745.88891	467.22222
333.09753	878.99999	467.99999
333.13138	1279.55552	649.22223
333.16522	1637	752.66669
333.19907	1538.11115	769.77778
333.23291	1003.11115	528.11109
333.26675	937.11115	595.44446
333.30057	1210.00004	550.00001
333.33441	1725.00004	790.99998
333.36823	1835.33341	1064.66666
333.40207	1351.88889	745.55558
333.43588	1007.22224	583.8889
333.4697	1431.11106	843.11106
333.50351	1755.00004	925.33329
333.53732	1868.22226	962.2222
333.57114	1481.99996	691.99999
333.60492	1172.55551	553.88889
333.63873	1442.55559	715.55553
333.67255	2135.55547	1119.88885
333.70633	2217.11103	1094.11115
333.74011	1827.55564	948.55552

333.77393	1627.88889	886.88889
333.80771	1777.77782	960.11113
333.84149	2139.66667	1065.66667
333.87527	2099.55547	1067.22222
333.90906	1442.00004	747.33335
333.94281	1250.11115	692.44446
333.97659	1768	983
334.01038	2047.99992	994.33329
334.04413	1998.99992	1029.66667
334.07791	1458.44445	706.44445
334.11166	1524.44449	733.7778
334.14542	2036.88897	1086.22222
334.17917	2296	1173
334.21292	1912.55555	918.22224
334.24667	1562.22218	754.55555
334.28043	1875.33333	989.66665
334.31418	2432.44436	1248.77773
334.3479	2405.22222	1242.5556
334.38165	1942.33333	991.66665
334.41537	1770.11107	950.77782
334.4491	2411.44444	1272.77781
334.48285	2529.11119	1252.77782
334.51657	2266.11111	1125.7778
334.55029	2030.33325	1023.33338
334.58401	2372.55556	1253.88894
334.61771	2781.7777	1440.77782
334.65143	2568.44444	1339.11107
334.68515	2285.66675	1221
334.71884	2431.11103	1251.4444
334.75256	2809.22214	1431.22226
334.78625	2859.55548	1491.5556
334.81995	2494.55564	1335.8889
334.85367	2656.2223	1368.88893
334.88736	3086.66659	1542.99996
334.92105	3061.55548	1491.5556
334.95471	2761.55548	1410.22222
334.9884	2758.44453	1361.11115
335.02209	3134.11119	1581.11107
335.05576	3316.33341	1653.33329
335.08945	3120.22214	1584.88889
335.12311	3031.66659	1584.99996
335.15677	3360	1715.33337
335.19046	3480.66659	1828.33333
335.22412	3288.44436	1699.77774
335.25778	3336.44437	1845.77786

335.29144	3542.11119	1836.11107
335.32507	3687.66659	1910.99996
335.35873	3465.77778	1791.44453
335.3924	3589.44436	1860.11111
335.42603	3856.11111	1969.11111
335.45966	3886.88897	2089.88897
335.49332	3695.11119	1884.11107
335.52695	4096.33317	2176.66667
335.56058	4232.22206	2156.88893
335.59421	4034.11111	2023.77774
335.62784	4246.55556	2258.55556
335.66147	4538.11127	2360.44452
335.69507	4432.55539	2304.22214
335.7287	4301.33317	2128.66667
335.76233	4803.33349	2466.66674
335.79593	4905.22239	2544.55564
335.82953	4639	2400.66675
335.86316	4950.88906	2508.22231
335.89676	5022.88905	2612.22231
335.93036	4839.55556	2492.22231
335.96396	5368.44461	2725.44436
335.99753	5368.22238	2739.22214
336.03113	5184.88889	2708.22214
336.06473	5524.33317	2842.66666
336.0983	5515.66666	2861.66666
336.1319	5586.6665	2855.66675
336.16547	5867.88906	2968.55556
336.19904	5787.11095	3012.1112
336.23264	6031.33317	3133.33341
336.2662	5911.11111	2980.77786
336.29977	6109.6665	3256.33325
336.33331	6370.55571	3281.55547
336.36688	6279.00016	3302.33341
336.40045	6350.77777	3178.77777
336.43399	6529.88889	3389.55564
336.46756	6823.66667	3573.33317
336.5011	6708.66683	3414.66659
336.53464	6927.77794	3578.44444
336.56821	7061.33333	3636.00008
336.60175	7337.11128	3824.11128
336.63528	7340.88873	3817.22222
336.66879	7458.33317	3784.33341
336.70233	7708.77778	3986.77778
336.73587	7766.22206	4010.88905
336.76941	7911.66667	4015.99992

336.80292	8260.55556	4270.88905
336.83643	8848.88921	4618.88872
336.86996	9905.33333	4969.99983
336.90347	11134.55556	5686.55556
336.93698	12793.66667	6441.00016
336.97049	15311.88889	7827.88889
337.004	18568.55621	9355.22222
337.03751	20206.00065	10171.33366
337.07098	16948.55491	8456.55588
337.10449	10122.88921	4993.55572
337.13797	4005.7777	1942.11107
337.17148	1374.77778	773.11109
337.20496	748.44447	409.11112
337.23843	331.55554	163.22222
337.27191	283	72.33333
337.30539	183.99999	135.33333
337.33887	205.11111	116.44444
337.37234	218.33332	221.66667
337.40582	153.00001	42.66667
337.43927	223.55556	154.88889
337.47275	221.99999	176
337.5062	295.44446	242.11108
337.53967	187.55557	161.55557
337.57312	244.99999	163.33333
337.60657	174.33334	116.33334
337.64001	124.1111	188.77779
337.67346	146.77778	47.11112
337.70691	124.88889	115.88889
337.74036	138.44445	78.11111
337.77377	52.22222	102.55556
337.80722	200.88891	139.22222
337.84064	127.77778	110.11111
337.87408	70.22223	88.22223
337.9075	90.33333	109.66667
337.94092	99.99999	94.99999
337.97433	105	73.33333
338.00775	144.44444	47.77778
338.04117	72.55556	92.22222
338.07458	93.77777	46.44445
338.10797	79.44444	65.11111
338.14139	85	71.66667
338.17477	31.88889	74.22222
338.20819	88.33334	54.66667
338.24158	189.22223	235.2222
338.27496	112.22223	97.22223

338.30835	79.55557	86.88888
338.34174	125.1111	47.44445
338.37512	65.55556	59.22222
338.40851	82.22222	20.55555
338.44186	45.11111	31.77778
338.47525	122.99999	61.00001
338.50861	179.33334	161.66665
338.54199	89.77778	60.11111
338.57535	31.88889	0
338.6087	48.44445	59.11112
338.64206	116	113.33334
338.67542	108.44444	30.11111
338.70877	112.66668	115.99999
338.74213	140.55557	46.88888
338.77545	145.77778	128.11111
338.80881	87.88889	59.88889
338.84216	85.33333	80.00002
338.87549	18.22222	21.55556
338.90881	55.55556	62.88889
338.94214	106.44444	108.11112
338.97546	128.77778	87.11111
339.00879	141	82.66666
339.04211	101.55555	84.22222
339.07544	5.22222	0
339.10876	105.44443	45.77778
339.14206	150.44443	146.44443
339.17538	81.66667	71.66667
339.20868	98.55555	80.55555
339.242	161.22222	131.55557
339.2753	191.33335	160.66667
339.30859	108.11111	107.44444
339.34189	107.55556	86.22222
339.37518	108.77778	97.44445
339.40848	73.22222	88.22222
339.44174	119.44445	143.11112
339.47504	81.22222	26.55555
339.5083	75.44444	57.11112
339.5416	196.7778	155.11111
339.57486	63.11111	71.11111
339.60812	146	78
339.64139	113.55555	111.22224
339.67465	46.66666	0
339.70792	22.88889	54.22222
339.74118	134.33334	96.99999
339.77444	122.77778	103.44444

339.80771	109.77778	177.11109
339.84094	54.99999	50.66667
339.87418	84.44445	52.11111
339.90744	90.77777	138.44446
339.94067	154	97.33334
339.97391	28.88889	52.88889
340.00714	61.88889	0.88889
340.04037	65.66667	69.33333
340.07361	217.88887	167.8889
340.10684	31.77778	42.77778
340.14005	145.33333	132
340.17328	141.11112	151.77778
340.20648	68.22222	43.88889
340.23972	73.66666	76.66666
340.27292	157.33333	127.66666
340.30612	85.11111	57.44444
340.33932	175.22224	119.88887
340.37253	136.66666	112.33333
340.40573	12.66667	0
340.4389	182.33333	170.00001
340.47211	91.88889	0
340.50531	82	29
340.53848	63.77778	8.77778
340.57166	102.33333	85.66668
340.60486	78.33333	61.66667
340.63803	150.88888	107.55556
340.6712	146.11111	80.77778
340.70438	79.44444	50.77778
340.73755	62.44445	15.44445
340.77069	95.33334	50.66667
340.80386	105.88889	114.88889
340.83701	69.33334	80.33334
340.87018	65.88889	35.22222
340.90332	94.66667	119
340.93646	122.00001	76.33333
340.96964	76.00001	86.00001
341.00278	124.22222	108.22222
341.03592	175.8889	121.55556
341.06903	178.88888	144.22222
341.10217	67.66667	0
341.13531	48.22223	46.55556
341.16843	35.88889	55.22222
341.20157	122.77778	61.44445
341.23468	128.77777	41.77777
341.26779	143.44443	43.1111

341.30093	132.77778	143.77778
341.33405	182.1111	83.44444
341.36716	146.11112	69.44444
341.40024	79.33333	18.33333
341.43335	53.00001	30.66666
341.46646	82.00001	46.66667
341.49954	171.55555	92.55555
341.53265	177.33334	79.33334
341.56573	238.55557	240.22222
341.59882	194.33334	137.33333
341.63193	151.99999	62.99999
341.66501	95.33334	16.33333
341.69809	36.66666	35.66666
341.73114	96.44445	82.44445
341.76422	156.66666	83
341.7973	96.44444	92.77778
341.83035	102.22222	94.88889
341.86343	102.77778	59.77778
341.89648	114.66667	123
341.92957	129.33333	69.99999
341.96262	121.11111	65.44445
341.99567	199.77778	83.77778
342.02872	115.66668	70.99999
342.06177	114.88889	33.55556
342.09479	49.88889	54.55556
342.12784	67.88888	20.88889
342.16089	152.11111	122.11111
342.19391	84.11111	35.77778
342.22693	15.22222	0
342.25998	73.66668	53.00001
342.293	166.44445	65.11111
342.32602	175.00001	187.33332
342.35904	87	83
342.39206	101.44445	83.11111
342.42505	24.88889	42.55555
342.45807	59.33333	15.33333
342.49109	23.44445	34.44445
342.52408	164.66667	106.33333
342.55707	152.33334	128.66666
342.59009	123.66668	67.33333
342.62308	201	154.66666
342.65607	84.22222	83.88888
342.68906	120.88889	133.22223
342.72205	160.44446	87.77779
342.755	86.88889	53.88889

342.78799	150.44444	71.11111
342.82098	195.77775	196.11112
342.85394	177.11113	97.77779
342.88693	116.66667	97
342.91989	66.66665	29.33332
342.95285	79.22221	136.22221
342.98581	109.11111	52.11111
343.01877	159.77778	139.77778
343.05173	94.22222	21.22222
343.08469	158.77778	159.77778
343.11761	149.44444	72.11112
343.15057	63.55555	99.22222
343.1835	110.77778	73.77778
343.21646	252.33332	117.33332
343.24939	253.55558	225.8889
343.28232	165.77778	75.77778
343.31525	80.00001	41.00001
343.34818	73.77779	52.11112
343.3811	98.11111	29.77778
343.41403	209.55556	151.88888
343.44693	154.55556	109.22223
343.47986	163.00001	78.66667
343.51276	196.66667	96
343.54568	180.33334	147.66667
343.57858	126.88889	73.55555
343.61148	80.77778	55.44445
343.64438	113.11111	43.11111
343.67728	83.77778	79.11111
343.71017	149.11112	82.77777
343.74307	163.22222	109.55556
343.77594	319.00002	201.33333
343.80884	240.22223	183.22222
343.84171	201.44445	138.44445
343.8746	85.66667	51.33333
343.90747	179.11112	87.44444
343.94034	157.66667	148.33334
343.97321	80.88889	0
344.00607	186.22222	105.55555
344.03894	249.44447	73.77778
344.07181	215.88889	127.55556
344.10464	172.33333	31.00001
344.13751	190.77779	135.1111
344.17035	185.22221	79.22221
344.20322	155.44445	69.77778
344.23605	193.33334	74

344.26889	223.44445	193.11113
344.30173	141.88889	94.55555
344.33456	207.77778	160.11111
344.3674	149.88889	36.22222
344.40024	286.55556	246.88888
344.43304	180.33333	85.00001
344.46588	203.33333	105.33334
344.49869	257.44445	184.77778
344.53152	216.77777	204.44446
344.56433	139.33333	6.33333
344.59714	86.11112	37.77778
344.62994	195.77778	139.44445
344.66275	172.33334	85.66667
344.69556	204.55556	73.55555
344.72836	80.55556	79.88889
344.76114	98.66666	78
344.79395	117.11111	92.11111
344.82672	187	109.33334
344.85953	184.55556	100.88889
344.8923	210.22223	171.55556
344.92508	310.55557	231.88888
344.95786	112.55556	48.55555
344.99063	118.33333	11
345.02341	121.11111	40.44444
345.05618	153.22223	118.55556
345.08893	184.22223	71.88889
345.1217	184.33334	80.33333
345.15445	199.44445	142.77778
345.18723	240.88888	154.22225
345.21997	293.77775	33.77778
345.25272	227.66667	157.66667
345.28546	195.55556	15.55556
345.31821	172.77777	119.44444
345.35095	150.55556	108.22222
345.38367	71.11111	36.44445
345.41641	128.66667	25.00001
345.44916	147.77778	95.77778
345.48187	298.11113	140.44445
345.51459	298.22221	121.88888
345.54733	435.11113	258.44445
345.58005	178.22222	99.55556
345.61276	144.66666	66
345.64548	155.88889	111.22222
345.67816	195.55556	48.88889
345.71088	312.88887	153.8889

345.74359	194.77778	70.44445
345.77628	140.77778	60.44444
345.80899	317.00001	149.66667
345.84167	343.77776	176.77779
345.87436	294.66668	93.66667
345.90704	193.22223	99.8889
345.93973	260.77779	109.11111
345.97241	200.99999	172.66665
346.0051	233.11111	144.11111
346.03778	306.22223	216.55555
346.07043	355.33333	230.66666
346.10312	301.66666	121.33333
346.13577	250.33333	129.66668
346.16846	206.66667	176
346.20111	351.88889	202.55556
346.23376	332.55553	222.88891
346.26642	239.1111	146.1111
346.29907	265.55555	197.55555
346.33173	309.77779	215.77779
346.36435	382.66669	227
346.397	125.11112	118.77778
346.42963	246.33333	144.33333
346.46228	338.66666	233.33334
346.4949	386.44442	175.77777
346.52753	317	159.66666
346.56015	335.55556	212.55555
346.59277	313	69.33334
346.6254	366.99998	249.99998
346.65802	222.33334	107.33333
346.69064	209.55555	143.22222
346.72324	187.66666	55.66667
346.75586	282.66666	109.00001
346.78845	468.1111	232.1111
346.82104	466.22222	169.22223
346.85364	435.66667	197.66667
346.88626	259.66665	162
346.91885	262.44446	107.11111
346.95142	258.55556	168.55556
346.98401	368.66667	148.66667
347.0166	293.33332	202.33332
347.04916	223.66668	60.33333
347.08176	467.66668	204.66666
347.11432	514.44445	233.77778
347.14688	494.44446	262.11112
347.17947	351.33333	160.00001

347.21204	155.22223	75.88889
347.2446	247.11111	152.77778
347.27713	422.77776	275.44445
347.30969	408.99998	243.66667
347.34225	444.99999	215
347.37479	336.11112	199.11111
347.40735	414.88887	270.55555
347.43988	572.88889	320.2222
347.47241	551.33335	333.00001
347.50497	309	182.33333
347.53751	344	162
347.57004	477.22221	233.8889
347.60254	402.55555	223.88889
347.63507	293.55555	124.88889
347.6676	269.22221	194.8889
347.7001	387.66669	149
347.73264	536.33333	230.33333
347.76514	596	197
347.79764	635.55553	280.88888
347.83014	487.55553	236.88888
347.86267	451.55557	292.8889
347.89514	426.11112	224.11111
347.92764	418.88892	297.22223
347.96014	403.33331	193
347.99265	496.00001	226.99999
348.02512	653.1111	260.77778
348.05762	779.77781	417.11112
348.09009	665.11111	341.77777
348.12256	443.33333	237.33333
348.15503	424.99999	155.33333
348.1875	367.55556	236.55556
348.21997	517.88887	206.55555
348.25244	564.77779	353.77778
348.28491	637.88889	325.88889
348.31735	589.88891	304.88888
348.34982	542.66669	239
348.38226	630.55556	339.88889
348.4147	526.8889	298.55556
348.44717	602.44446	318.77778
348.47961	661.22219	337.55556
348.51205	511.55554	181.22222
348.54449	453.88889	196.22223
348.5769	482.33333	199.66668
348.60934	545.88889	281.55555
348.64178	694.77778	272.77778

348.67419	991.5556	496.22222
348.7066	911.5556	434.22222
348.73904	593.7778	245.44444
348.77145	533.22223	267.88889
348.80386	473.55557	260.22222
348.83627	441.00001	226.99999
348.86868	709.22224	331.8889
348.90109	692.11111	303.44444
348.93347	1019.00002	541.33333
348.96588	934.55559	514.88891
348.99826	681.44446	296.11112
349.03067	588.77778	337.44445
349.06305	591.2222	342.22223
349.09543	755.88891	442.22222
349.12781	758.99999	330.66668
349.16019	835.44442	480.44445
349.19257	648.00001	245.33333
349.22495	896.11107	458.44445
349.25729	934.77778	458.44447
349.28967	821.44447	378.77778
349.32202	878.33335	413.33332
349.3544	868.55552	431.2222
349.38675	689.88889	308.22222
349.4191	655.77776	381.11111
349.45145	760.33333	391.00002
349.4838	1038.44449	462.11111
349.51614	1266.00004	670.99998
349.54846	1175.55556	622.22224
349.58081	805.33333	329
349.61316	708.66665	323.00002
349.64548	901.5556	516.88892
349.6778	840.11113	468.77777
349.71014	1011.55558	433.22222
349.74246	1153.88885	525.88891
349.77478	1206.22218	595.88887
349.8071	1168.55554	579.88888
349.83939	1314.4444	589.11112
349.8717	1383	715.33331
349.90402	1631.44445	795.44445
349.93631	1523.66663	792
349.9686	1154.88885	590.55554
350.00092	871.55553	433.88891
350.0332	892.2222	394.55555
350.06549	1078.77778	531.44443
350.09778	1136.66664	678.00001

350.13007	789.33335	451.99998
350.16235	612.44446	292.11111
350.19461	668.66667	488.99998
350.2269	665.33331	357.33334
350.25916	780.44444	414.11113
350.29144	933.33333	467.66665
350.3237	1093.88893	550.22221
350.35596	982.00002	474.99999
350.38821	754.66666	340.00001
350.42047	865.55556	429.88887
350.45273	1015.5556	529.88891
350.48499	855.88891	391.88888
350.51721	728.00004	496.66667
350.54947	772.11109	374.44443
350.5817	1074.22222	538.55557
350.61395	1425.11115	718.11109
350.64618	1132.66667	584.66667
350.67841	701.88889	324.88889
350.71063	660.88891	288.22223
350.74286	881.66662	438.66668
350.77509	1095.55556	672.88887
350.80731	1060.44441	521.77778
350.83951	1158.33334	590.33331
350.87173	1198.77782	521.44444
350.90393	1177.00004	535.66667
350.93613	1046.88889	441.2222
350.96835	966.11109	391.11111
351.00055	1005.99996	513.00002
351.03275	780.66665	386.66668
351.06494	1156.88889	637.88889
351.09714	1450.77782	712.77776
351.1293	1541.88893	725.55556
351.1615	1123.22222	524.88891
351.19366	905.66671	448.00003
351.22586	1054.33332	467.66665
351.25803	1237.22218	602.88887
351.29019	1036.44448	561.7778
351.32236	1190.22222	631.55553
351.35452	1495.11107	699.1111
351.38669	1691.22226	854.2222
351.41885	1504.11116	816.77778
351.45099	1301.22218	637.88887
351.48315	1110.22222	576.88888
351.51532	1151.11115	557.44443
351.54745	1423.00004	715.66667

351.57959	1612.11111	844.44449
351.61172	1646.66667	813.66667
351.64386	1605.00004	810.33335
351.67599	1486.77778	768.77778
351.70813	1452.77778	716.11109
351.74026	1219.44445	682.11113
351.77237	1354.33329	713.99998
351.8045	1660.22222	813.55554
351.83661	1952.99999	1112.33336
351.86874	1816.22222	948.55554
351.90085	1672.4444	865.11115
351.93295	1557.66671	830.33333
351.96506	1509.11116	768.11116
351.99716	1365.11115	696.44446
352.02927	1635.88893	808.88887
352.06137	2057.55564	1038.22226
352.09344	2080.99992	1027.00004
352.12555	1792.66662	879
352.15762	1769.22222	820.88891
352.1897	1907.7777	983.77782
352.2218	1723.22222	864.55553
352.25388	1906.11111	1055.11111
352.28595	2040.3333	1033.66667
352.31799	2283.66658	1183.99995
352.35007	2050.88897	1052.55559
352.38214	1925.88893	917.88887
352.41422	1921	1013.66669
352.44626	1849.66667	925.33329
352.4783	2050.88889	1026.22226
352.51038	2283.55548	1267.88885
352.54242	2433.33341	1252.33329
352.57446	2388.00008	1258.99996
352.60651	2208.22222	1056.55559
352.63855	2063.6667	1059.33333
352.67056	2091.33333	945.00002
352.70261	2179.88881	1120.22218
352.73465	2620.2223	1342.22218
352.76666	2914.55564	1519.22226
352.79868	2660.99992	1409.33329
352.83072	2291.77778	1129.4444
352.86273	2216.7777	1050.11107
352.89474	2291.33342	1250.3333
352.92676	2507.22231	1243.22224
352.95874	2765.44445	1533.77782
352.99075	3131.22214	1567.55551

353.02277	2770.99992	1280.99998
353.05475	2463	1272
353.08676	2461.00008	1289.99996
353.11874	2620	1299.33337
353.15073	2686.44436	1354.77774
353.18271	3199.2223	1703.55556
353.21469	3249.55556	1681.55556
353.24667	3014.33341	1430.66667
353.27866	2703.22222	1345.22222
353.31064	2780.88881	1402.55555
353.34259	3011.77778	1622.11115
353.37457	3462.88897	1807.88897
353.40652	4160.66667	2172.33342
353.43848	4275.88905	2185.2223
353.47043	4394.22206	2324.8888
353.50238	4989.11095	2629.44444
353.53433	5726.11111	2936.11111
353.56628	5808.22222	3005.88897
353.59824	4994.22222	2575.88897
353.63019	3488.77778	1649.4444
353.66211	1891.4444	808.77778
353.69406	1078.77776	483.77779
353.72598	1135.66662	666
353.7579	1240.66667	650.33329
353.78983	1502.55555	738.88887
353.82175	1569.66667	798.66667
353.85367	1190.66663	615
353.88559	1069.11108	621.44446
353.91751	1073.22218	616.22224
353.9494	1293.22226	577.55555
353.98132	1503.44448	796.11111
354.01321	1764.44445	895.11107
354.0451	1308.22222	650.55554
354.07703	1170.55552	662.55558
354.10892	1257.44441	709.11116
354.14081	1567.22218	901.22218
354.1727	1826.77778	896.11109
354.20456	1774.88893	875.88887
354.23645	1500.77777	789.77777
354.26834	1165.55552	572.2222
354.3002	1390.77778	751.77778
354.33206	1550.22222	694.55554
354.36395	1932.66675	1062
354.39581	1822.33333	945.99996
354.42767	1413.88885	725.88891

354.45953	1302.77778	692.11109
354.49139	1601	801.33331
354.52322	1923.77778	950.11115
354.55508	1929.00004	1001.33336
354.58691	1727.88889	894.88889
354.61877	1526.99996	785.99996
354.6506	1655	845.33337
354.68243	1894.88893	1042.88887
354.71426	2130.55552	1109.2222
354.74609	2002.22222	965.22222
354.77792	1798.88889	873.22226
354.80975	1721.55551	994.88889
354.84158	1944.11115	969.44447
354.87338	2200.77786	1133.77774
354.90521	2104.11111	1075.11111
354.93701	1824.1111	924.77779
354.96884	1728.77778	839.77778
355.00064	2152.44436	1152.11111
355.03244	2290.99999	1132.66662
355.06424	2234.33333	1209.33333
355.09604	1954.88889	978.55557
355.12781	2090.00008	1116.66671
355.15961	2196.99992	1136.66667
355.19138	2375.88881	1278.88893
355.22318	2491.88897	1321.88884
355.25494	2189	1148.33337
355.28671	2086.66667	1071.66667
355.31851	2578.00008	1422.33333
355.35028	2639.33325	1424.33337
355.38205	2514.22214	1420.22226
355.41379	2220	1138.66663
355.44556	2400.88889	1287.22226
355.47733	2681.55563	1298.88889
355.50906	2800	1482.33338
355.54083	2535.22214	1257.88889
355.57257	2540.88897	1326.5556
355.60431	2836.33325	1413
355.63605	3011.66675	1647.33337
355.66779	2764	1411.66663
355.69952	2654	1523
355.73126	2924.22231	1565.55556
355.76297	3223.22214	1729.55563
355.79471	2961.7777	1602.77782
355.82642	2806	1465.33337
355.85815	3076.66658	1548.99995

355.88986	3385.33341	1738.33329
355.92157	3169	1662
355.95328	2818.55548	1417.22222
355.98499	3187.44453	1739.77778
356.01669	3552.22214	1836.88889
356.0484	3347.66675	1721
356.08008	2966.33341	1512.33329
356.11179	3359.55547	1831.5556
356.14346	3729.22214	2012.88889
356.17514	3562.8888	1806.55555
356.20685	3236.99992	1686.3333
356.23853	3699.66675	1962.33337
356.2702	3850.11119	2026.11119
356.30188	3564.33333	1952.33333
356.33353	3563.22222	1905.88885
356.3652	3983.77769	2146.77782
356.39688	3939.77786	2024.44448
356.42853	3618.55547	1816.5556
356.46021	4110.22222	2294.55547
356.49185	4199.33333	2239.00008
356.5235	3922.33333	2072.00008
356.55515	3960.33317	2064.66667
356.58679	4395.44444	2218.44444
356.61844	4329.00009	2168.99997
356.65005	4392.88872	2380.22222
356.6817	4386.33333	2288.00008
356.71335	4419.77761	2260.44436
356.74496	4447.99984	2348.33333
356.77658	4567.88889	2438.55564
356.80823	4590.44428	2450.77778
356.83984	4588.11095	2383.44444
356.87146	4794.77794	2577.44445
356.90308	4805.33317	2514.99992
356.93466	4965.11127	2547.77778
356.96628	4849.77794	2468.44445
356.99789	5289.3335	2758.33325
357.02948	4918.55539	2564.88889
357.06107	5238.3335	2711.66675
357.09268	5307.99983	2895.66658
357.12427	5210.00001	2711.66676
357.15585	5454.33317	2821.33342
357.18744	5498.22222	2849.55547
357.21902	5586.55539	2924.55564
357.25058	5653.88905	2866.2223
357.28217	5734.88873	2973.22222

357.31375	5934.88873	3080.22222
357.34531	5964.66683	3040.66659
357.37686	6336.88873	3283.22222
357.40845	7165.3335	3660.33325
357.44	7784.66683	4032.66683
357.47156	8736.11111	4512.77761
357.50311	10219.55588	5419.55539
357.53464	12347.11144	6334.44445
357.56619	13876.66699	7113.6665
357.59775	12916.55556	6533.55556
357.62927	9416.77745	4699.77794
357.66083	5021.77794	2488.7777
357.69235	1845.55563	969.22226
357.72388	725.11113	415.77779
357.7554	349.44446	149.11111
357.78693	196.33333	155.33333
357.81845	339.66667	235.99998
357.84998	165.44444	116.77777
357.88147	142.66667	48.66667
357.91299	135.55555	66.88889
357.94449	87.33333	71
357.97598	193.44444	156.77778
358.00751	81.00001	40.66668
358.039	122.66667	74.66666
358.0705	138.44446	39.44446
358.10199	140.33333	145.00001
358.13348	89.55556	82.55556
358.16495	89.99999	81.33334
358.19644	154.44447	135.77778
358.22791	163.66666	85.33333
358.2594	98.77778	55.44445
358.29086	123.11111	79.77778
358.32233	103.22223	31.22222
358.35379	24.77778	0
358.38525	67.22222	36.22221
358.41672	78.88889	56.88889
358.44818	108.55556	123.55556
358.47961	79.77777	57.44444
358.51108	83	55
358.54251	181.44445	130.77776
358.57397	31.00001	0
358.60541	39.33334	7.33333
358.63684	80.11111	57.44445
358.66827	112.44446	86.77777
358.69971	121.66667	92.33334

358.73114	171.88888	171.22222
358.76254	110.33334	76.33334
358.79398	98.11111	124.77777
358.82538	105.33333	82.66667
358.85681	79.44445	51.44445
358.88821	3.88889	14.88889
358.91962	56.44444	154.11112
358.95102	58.11112	22.44444
358.98242	118.11111	61.77778
359.01382	133.33334	67.33333
359.04523	119.88889	110.55556
359.0766	138.55556	122.55556
359.108	52.44444	18.77778
359.13937	88.22223	3.22223
359.17078	182.44446	214.11108
359.20215	96.11111	65.11111
359.23352	174.99999	186.33334
359.26489	86.11111	74.77778
359.29626	102.66667	19.66667
359.32764	83.77778	99.44444
359.35898	63.11111	0.44445
359.39035	115.88889	36.22222
359.42169	149.66666	108
359.45306	157.66668	44.33333
359.48441	76.33334	32.66667
359.51575	136.11111	68.44444
359.54709	72.55556	92.22221
359.57843	60.22223	17.55555
359.60977	68.33333	110.33333
359.64111	166.88889	90.88889
359.67242	56.33333	48.66667
359.70377	149.11111	53.11111
359.73508	65.66667	45
359.76639	132.44444	125.77778
359.79773	92.11112	126.77779
359.82904	62.11111	0
359.86035	79.55556	46.88889
359.89166	140.33333	119.33333
359.92294	156.88889	114.88889
359.95425	133.44444	100.11111
359.98557	135.11111	76.11111
360.01685	22.77778	2.77777
360.04813	51.11111	42.77778
360.07944	82.55554	52.22223
360.11072	212.44445	198.11111

360.142	139	119.33334
360.17328	116.11111	81.77778
360.20456	163.77778	26.77778
360.23581	102.33333	85
360.26709	73.22222	0
360.29834	38.33334	28.33334
360.32962	193.88891	130.55553
360.36087	80.22222	33.55556
360.39212	90.11111	45.77778
360.42337	115.55556	78.55556
360.45462	142.11111	69.44445
360.48587	95.55556	121.88889
360.51712	103.77778	112.11111
360.54837	69	46
360.57959	51.11111	45.77778
360.61084	51.55555	37.88889
360.64206	146.77779	118.44444
360.67328	123.1111	82.77779
360.7045	185.2222	159.55557
360.73572	141.55556	109.55556
360.76694	71	27.33333
360.79816	147.55555	117.55556
360.82938	76.11111	59.77777
360.86057	172.77778	140.44444
360.89178	183.55555	128.55555
360.92297	55.22222	54.55556
360.95416	130.22222	103.55557
360.98538	88.44445	52.44445
361.01657	185.33334	109.66667
361.04776	57.33334	4.33334
361.07892	175.33334	119.33334
361.11011	87.33332	50.66667
361.1413	186.55556	133.55555
361.17245	121.99999	77.66667
361.20364	211.33334	116.33333
361.2348	78.33334	58.33334
361.26596	126.66667	15.33333
361.29712	82.77777	106.44444
361.32828	101.33334	51.66667
361.35944	120.55555	109.22222
361.39059	179.88889	68.88889
361.42175	159.55556	151.55556
361.45288	277.22221	161.55556
361.48404	109.11111	35.11111
361.51517	237.66667	192.99999

361.5463	119.88889	70.22223
361.57745	72.66667	0
361.60858	140.55556	121.88889
361.63968	150.77778	76.11111
361.67081	204.55556	203.88887
361.70193	105.44445	78.77778
361.73306	29	6.33334
361.76416	212.88889	117.88889
361.79529	432.33333	372.33333
361.82639	278.99999	187.3333
361.85748	200.11111	107.44444
361.88858	210.66666	87.33333
361.91968	136.11111	88.11111
361.95078	119.55557	76.88888
361.98187	41.11111	38.77777
362.01294	174.77778	107.44445
362.04404	171.11112	124.11112
362.0751	171.11111	83.44444
362.1062	174.33333	107.66667
362.13727	188.44445	93.11111
362.16833	166.22222	84.22222
362.1994	156.33335	61.33333
362.23047	159.22222	70.8889
362.26154	167.11111	7.44444
362.29257	187.44444	122.44444
362.32364	89.77777	65.77777
362.35468	286.00001	146.33334
362.38574	329.11111	160.77777
362.41678	87.00001	23.33333
362.44781	177	22.33333
362.47885	201.44443	95.77778
362.50989	162.88889	62.88889
362.54092	159.33333	103.99999
362.57196	219.66668	124.66666
362.60297	159.22222	87.22222
362.634	229.22222	132.22222
362.66501	277.44443	205.44443
362.69604	194.22222	80.55556
362.72705	250.11111	201.44444
362.75806	294.44445	129.44445
362.78906	155.88889	97.55555
362.82007	240.22223	118.22223
362.85104	173.88888	156.55556
362.88205	288.66669	192.66669
362.91306	164.8889	102.22223

362.94403	223.33333	114.00001
362.97501	226.33334	98.66667
363.00601	313.22221	151.88888
363.03699	270.99998	166.00001
363.06796	70.55556	27.55556
363.09894	85.11111	34.44445
363.12991	160.77777	82.11112
363.16086	197.99999	162.33333
363.19183	221.66666	83
363.22278	231.99999	188.33333
363.25375	246.44443	105.44445
363.2847	297.55554	180.55557
363.31564	254.77778	110.77778
363.34659	380.99998	282.33332
363.37753	307.22223	75.88889
363.40848	265.8889	93.22222
363.43942	154.77778	112.77778
363.47034	98.66667	62.33334
363.50128	171.22223	40.22222
363.5322	206.66667	105.33333
363.56314	293.88889	124.55556
363.59406	308.11111	199.11111
363.62497	250.33333	162.66668
363.65588	123.11111	25.11111
363.6868	256.99999	111.99999
363.71768	293.55555	180.88889
363.7486	262.22222	83.22222
363.77951	361.33335	212.33332
363.81039	302.66668	135.99999
363.84128	348	215
363.87219	334.1111	223.1111
363.90308	236	143.33333
363.93396	332.22225	276.55556
363.96484	313.55556	248.8889
363.9957	339.66666	195.66666
364.02658	284.00001	128.66667
364.05746	313.44445	114.77779
364.08832	376.11112	121.11111
364.1192	436.66668	172.33333
364.15005	462.44442	234.44442
364.18091	312.00001	101.33334
364.21176	293.33334	199.66667
364.24261	258.99999	65.66666
364.27347	236.11111	179.11111
364.30432	236.55554	161.22223

364.33514	224.00001	99
364.366	271.11112	138.77778
364.39682	418.88887	262.88887
364.42764	362.66667	204.00001
364.4585	251.77778	144.77778
364.48932	364.44444	164.11112
364.52014	221.44443	141.1111
364.55093	198.55556	122.22221
364.58176	183.44446	105.77778
364.61258	275.55558	179.22223
364.64337	322.66667	175.33332
364.67419	311.66667	167.33335
364.70499	216.33333	107
364.73578	187.66667	94.66667
364.7666	289.8889	162.55556
364.79739	306.66666	130.66667
364.82816	307.55555	180.55555
364.85895	256.77778	62.11111
364.88974	217	142.66667
364.9205	317.22223	168.22222
364.95129	288.88889	158.88889
364.98206	303.88888	211.22222
365.01285	247.55555	96.88889
365.04361	343.22224	255.55555
365.07437	240.66666	99
365.10513	350.66668	176.00001
365.13586	383.88888	178.88888
365.16663	355.11113	173.1111
365.19739	421.11113	262.44444
365.22812	213	82
365.25888	295.55556	197.22221
365.28961	240.99999	113.33333
365.32034	246.66666	95.33334
365.35107	220.22222	99.22222
365.38181	344.88889	169.55555
365.41254	487	285.66669
365.44327	385	157
365.47397	504.33333	204.66666
365.5047	353.55557	169.8889
365.5354	249.99999	154.33333
365.56613	300.88889	187.55556
365.59683	272.33334	173.99999
365.62753	282.66666	163.66667
365.65823	369.1111	162.44444
365.68893	249.11111	129.77778

365.7196	421.8889	261.8889
365.75031	373.77776	183.1111
365.78101	351.77779	122.77777
365.81168	461.22223	196.22222
365.84235	458.8889	184.22222
365.87305	390.55557	174.22223
365.90372	381.55555	209.8889
365.93439	410.33332	183.66667
365.96506	290.11111	98.11111
365.9957	317.77779	245.77779
366.02637	279.33333	89.66667
366.05704	344.44444	174.44444
366.08768	561.44446	363.77777
366.11835	572.88887	297.55556
366.14899	693.22222	351.22222
366.17963	517.33332	250.66667
366.21027	367.88891	160.55556
366.24091	382.44445	150.44444
366.27155	376.77777	169.11111
366.30215	406.77779	214.44444
366.33279	446.66664	193.66667
366.36343	528.66667	326.66667
366.39404	479.00002	234.33333
366.42465	417.8889	221.55556
366.45526	564	260
366.48587	441	223.66666
366.51648	436	232.33334
366.54709	418.44445	185.44445
366.5777	538.77776	318.44445
366.60831	637.55554	378.55554
366.63889	527.33333	229.99999
366.66949	514.11112	310.77778
366.70007	494.7778	288.11111
366.73065	558.66664	267.66667
366.76123	688.11115	363.44446
366.79181	606.55557	324.55555
366.82239	592.33332	245.33334
366.85297	559.11113	288.44444
366.88351	598.99998	281.33334
366.91409	630.88891	319.88891
366.94464	532.77778	287.44445
366.97522	781.44442	398.11111
367.00577	977.55555	498.88887
367.03632	1009.77774	513.11111
367.06686	967.77776	461.1111

367.09741	791.99998	400.33332
367.12796	648.22222	328.55557
367.15848	465.44444	267.11112
367.18903	311	186.66667
367.21954	352.11111	188.77777
367.25009	265.33334	158.66666
367.28061	247.22223	93.55556
367.31113	279.55556	124.88888
367.34164	368.11111	224.77777
367.37216	466.55558	173.88889
367.40268	463.88889	215.22222
367.4332	496.44442	243.44445
367.46368	354.55554	156.22222
367.4942	294.55555	106.88888
367.52469	222.22222	101.22223
367.55518	291.77777	180.44444
367.58566	310.66666	207.66666
367.61615	291.44446	136.44444
367.64664	493	299
367.67712	505.99998	292.00001
367.70761	572.88889	354.55558
367.7381	401.22223	196.88889
367.76855	392.11111	254.11111
367.79901	382.8889	188.22223
367.8295	441.77778	245.11112
367.85995	484.7778	338.44443
367.89041	290.22223	137.55556
367.92087	377.44447	200.77778
367.95132	557.22221	277.55558
367.98178	541.66665	297.99999
368.01221	460.44443	246.11111
368.04266	409.33333	186.00001
368.07309	320.22221	154.55555
368.10352	282.11111	82.11111
368.13397	299.44445	290.1111
368.1644	375.99999	202
368.19482	461.88888	211.55555
368.22525	636.33331	407
368.25565	571.55556	289.22221
368.28607	702.33335	432.66667
368.31647	577.11113	338.77779
368.34689	456.88891	286.22222
368.37729	279.33333	151
368.40768	347.33332	188.66667
368.43811	393.11111	242.11111

368.46851	456.44444	199.77779
368.49887	496.00002	284.66668
368.52927	597.33333	334.66666
368.55966	773.66669	394.66669
368.59003	685.66669	262
368.62042	537.22222	225.55556
368.65079	453	193.66667
368.68115	458.66665	241.33333
368.71155	397.88889	207.88889
368.74191	502.88887	281.8889
368.77228	465.66668	251.66667
368.80261	628.2222	344.55557
368.83298	557.33334	263.33334
368.86334	634.33331	285.66667
368.89368	598.88891	273.88888
368.92401	605.44446	332.11108
368.95438	525.99998	278.33333
368.98471	476.88888	271.55556
369.01505	369.33334	145.33333
369.04538	473.77778	258.11112
369.07571	660.88889	358.88889
369.10602	754.33335	310.33334
369.13635	685.77778	297.77778
369.16666	652.55553	330.22222
369.19699	647.55553	282.22222
369.22729	582.00001	365.33334
369.2576	602.33335	271.00001
369.2879	617.77778	338.44443
369.31821	739.77776	399.11113
369.34851	745.55558	414.22223
369.37881	937.55551	429.55557
369.40909	793.55552	416.22221
369.43939	662.33333	316.66668
369.46967	630.00002	361.33333
369.49994	670.22224	364.8889
369.53024	565.22221	280.8889
369.56052	564	281.66666
369.59079	671.55556	375.8889
369.62106	1033.22226	565.2222
369.65131	1017.88887	447.8889
369.68158	912.55555	405.55555
369.71182	797.66667	458.66667
369.7421	748.44442	376.11111
369.77234	686.44442	415.44445
369.80258	682.22222	455.55553

369.83282	614.88891	339.55556
369.86307	913.88887	482.88887
369.89331	913.44444	451.77779
369.92355	1022.66663	534.66669
369.9538	875.22224	422.88888
369.98401	964.66665	492.99999
370.01425	835.5556	435.22222
370.04446	809.55556	457.22225
370.07468	788.55553	359.55556
370.10489	843.88891	413.88891
370.1351	1053.88889	544.2222
370.16531	1225.5556	665.88891
370.19553	937.66669	432
370.22571	1019.33337	584
370.25592	762.11111	417.44445
370.2861	757.77776	349.77776
370.31631	881.33335	468.66667
370.3465	1150.77782	603.77776
370.37668	1150.77782	555.44444
370.40686	1188.33333	557.00002
370.43704	1231.99996	587.00002
370.46722	1087.44447	520.77778
370.49738	860.77776	410.1111
370.52756	759.22224	356.8889
370.55771	749.33331	411.33334
370.58789	921.5556	451.22222
370.61804	1212.11115	601.11109
370.64819	1355.44444	701.11113
370.67834	1286.77774	544.11111
370.7085	1349.22218	684.88887
370.73865	1332.66667	697.00004
370.76877	1149.22226	557.2222
370.79892	1376.00004	712.33335
370.82904	1698.77778	911.11109
370.85919	1976.33338	957.66669
370.88931	2110.77786	1003.44449
370.91943	2068.66659	1055.33333
370.94955	1580.99996	727.00002
370.97968	1255.66671	653.33333
371.0098	858.88891	459.55557
371.03992	682	339.66666
371.07001	604.11111	335.11111
371.10013	626.44444	420.11112
371.13022	576.2222	253.88889
371.16031	472.55555	295.55556

371.1904	422.99998	249.33333
371.22049	187.99999	108.33334
371.25058	198.33333	73.66667
371.28067	396.77777	183.11111
371.31076	531.22222	233.22222
371.34082	770.66665	364.00002
371.37091	769.11111	347.44446
371.40097	538.88888	277.22222
371.43106	332.00001	162.33334
371.46112	247.1111	113.44444
371.49118	299.66666	167.33332
371.52124	447.22222	198.22222
371.55127	583.44445	282.77778
371.58133	657.11109	404.44446
371.61139	530.44442	372.11111
371.64142	355	145
371.67148	430.66667	196.33334
371.70151	526.44444	230.44444
371.73154	716.66664	429.00001
371.76157	644.55554	355.88888
371.7916	687.33335	298.33332
371.82162	653.22224	303.55556
371.85165	425.44442	241.77777
371.88165	238.11112	155.11111
371.91168	314.44444	197.11111
371.94168	472.55556	187.55556
371.97168	789.88889	397.2222
372.00168	964.22226	539.88889
372.03171	741.00001	318.00001
372.06168	499.1111	241.11112
372.09167	459.5556	384.22223
372.12167	394.33332	248.66666
372.15167	629.66667	400.66667
372.18164	828.22218	531.88887
372.21164	721.88891	347.88888
372.24161	671.11113	340.44444
372.27158	468.33331	252
372.30154	562.33331	284
372.33151	866.44447	503.11109
372.36148	872.11109	439.44443
372.39145	832.55553	375.22222
372.42139	638.22224	261.55556
372.45135	551.11113	257.77779
372.48129	379.33334	213
372.51123	464.00001	288

372.54117	657.55558	399.88889
372.57114	683.66669	325.66666
372.60107	949.88887	576.8889
372.63098	938.22218	475.55555
372.66092	846.1111	449.44442
372.69086	712.44444	388.77776
372.72076	671.99997	324.99999
372.7507	749.33331	412.66666
372.78061	802.11111	453.77779
372.81052	660.77776	400.1111
372.84042	555	285.66666
372.87033	651.33335	437.33335
372.90024	918.22226	510.88889
372.93015	1011.66667	448.66667
372.96002	1179.66663	566.33334
372.98993	928.44442	460.77777
373.01981	581.88887	272.55555
373.04971	573.33333	345.33333
373.07959	763.00002	459.33333
373.10947	883.88887	516.88887
373.13934	813.33336	373.33333
373.16922	819.77778	422.11109
373.19907	805.00002	417.66666
373.22894	995.66665	452.99999
373.25879	1212	660
373.28867	1106.88893	526.22224
373.31851	972.33333	472.66668
373.34836	850.55558	412.22223
373.3782	758.55555	387.22221
373.40805	844.55556	474.22224
373.4379	1092.88893	567.88887
373.46774	1069.44449	472.11111
373.49759	1157.33337	621.66669
373.5274	1108.77778	546.11109
373.55725	1121.11115	655.44447
373.58707	1005.55551	506.88889
373.61688	1098.88893	656.55555
373.6467	978.88889	500.88889
373.67651	867.22222	450.55556
373.70633	744.11113	366.44445
373.73615	1003.11109	520.11112
373.76593	1339.77778	740.44447
373.79575	1316.33337	658
373.82553	1327.66671	579.33333
373.85535	1346.1111	772.77773

373.88513	1149.22226	586.2222
373.91492	1126.11113	552.77779
373.9447	1106.11115	578.44447
373.97449	959.66667	527.66667
374.00424	1000.99998	461.66667
374.03403	1269.77782	644.77779
374.06381	1482.44448	704.7778
374.09357	1408.8889	759.8889
374.12332	1399.33337	679.66669
374.15308	1275.11116	622.11112
374.18286	1344.77778	614.77778
374.21259	1202.22218	648.88887
374.24234	1167.77774	659.11111
374.27209	1220.66663	632
374.30185	1405.11107	683.77776
374.33157	1348.88893	663.88887
374.36133	1635.22226	776.88889
374.39105	1784.77782	884.77782
374.42078	1608.33329	803.99998
374.4505	1426.77773	850.44448
374.48022	1180.33333	558.66668
374.50995	1379.99996	729.66665
374.53967	1440.88885	715.22222
374.56937	1389.88885	771.88891
374.59909	1647.11107	780.44445
374.62878	1838.77778	967.44447
374.65851	1854.88885	966.55554
374.6882	1848.88897	983.88884
374.7179	1421.88893	673.22224
374.74759	1358.22222	749.55554
374.77728	1410.22226	709.88889
374.80695	1421.22218	591.55556
374.83664	1672.55547	915.22222
374.86633	1775.66671	858.33333
374.896	2011.33333	1061.33333
374.92566	2013.99992	1005.66667
374.95532	1757.33337	836.33331
374.98502	1600.77782	858.77776
375.01468	1536.33329	738.99998
375.04431	1876.44454	1180.11104
375.07397	1606.5556	836.88891
375.10364	1780.99996	882.00002
375.13327	1911.11115	969.77778
375.16293	2144.44452	1080.11115
375.19257	2130.55547	1048.88885

375.2222	2115.22226	1059.88889
375.25183	2098	1064
375.28146	2268.66659	1275.99996
375.3111	2554.11103	1416.11116
375.34073	3144.77786	1604.77774
375.37033	3603.11111	1870.11111
375.39996	3980.11127	2080.77777
375.42957	3686.22214	1836.55552
375.4592	2780.55564	1357.22226
375.4888	1997.44445	913.11107
375.5184	954.44448	512.44442
375.548	454.77776	245.44444
375.57761	318	192.66667
375.60718	403.88889	261.88889
375.63678	675.99999	370.99999
375.66635	887.88888	513.88888
375.69595	666.33334	373.99999
375.72552	415.55555	324.88889
375.7551	98.1111	30.77777
375.78467	122.44445	0
375.81424	325.55558	213.22223
375.84381	418.8889	245.88888
375.87338	593.99998	306.33335
375.90292	743.77776	340.44444
375.9325	586.8889	284.22223
375.96204	493.55558	305.22223
375.99161	161.77777	120.77778
376.02115	129.1111	113.77779
376.05069	203.66667	127.33333
376.08023	418.33333	286.99999
376.10977	597.7778	378.44442
376.13928	768.66665	387.33333
376.16882	649	363.66667
376.19833	477.55555	226.55556
376.22787	267.77777	214.44446
376.25739	252.55557	102.88889
376.2869	230.44444	137.11111
376.31641	676.55558	401.55558
376.34592	774.55555	506.55555
376.37543	847.55556	382.22221
376.40494	517.22223	298.22221
376.43442	297.44446	168.11111
376.46393	259.88889	229.88889
376.49341	278.55557	183.88889
376.52289	476.55554	285.22222

376.5524	583.55556	242.88888
376.58188	799.22222	412.55557
376.61136	809.33337	417
376.64081	501	268.33334
376.67029	290.22222	153.22222
376.69977	272.44443	199.44443
376.72922	434.7778	264.77777
376.75867	525.44444	277.11112
376.78815	776.99998	391.66667
376.8176	919.44448	481.11111
376.84705	693.33331	380
376.8765	446.77777	253.11111
376.90594	333.11111	187.77778
376.93536	399.2222	212.55554
376.96481	669.66669	363.66669
376.99423	834.11113	462.1111
377.02368	904.66668	442.99999
377.0531	766.77778	377.77778
377.08252	497.33331	322.33334
377.11194	276.44445	165.44445
377.14136	419.33332	215.99999
377.17078	673.44446	402.44446
377.20016	783.22222	403.55557
377.22958	898.77778	446.44445
377.25897	821.22222	437.88888
377.28839	447.33333	276.33333
377.31778	325.44444	207.44444
377.34717	526.11111	262.44445
377.37656	834	489
377.40594	815.33333	408.99999
377.43533	937.22218	479.88887
377.46469	709.44442	316.11111
377.49408	439.66666	209
377.52344	526.11111	355.44443
377.55283	682.33335	395.00001
377.58218	834.88889	473.88889
377.61154	981.55558	444.22223
377.6409	900	536.33334
377.67026	705	353.66665
377.69962	600.11113	348.77776
377.72894	594.33334	311.66667
377.7583	802.11111	418.44445
377.78763	1176.77777	780.77777
377.81699	1015.55554	559.88891
377.84631	869.22222	460.22222

377.87564	652.22224	378.88887
377.90497	655.99998	360.66667
377.9343	815.11109	442.77778
377.96362	1004.22225	493.55556
377.99292	1167.88893	595.22224
378.02225	845.55559	414.55553
378.05154	750.22222	423.55556
378.08087	665.11111	334.44445
378.11017	932.66669	500
378.13947	1012.33331	450.33334
378.16876	1202.77774	589.11111
378.19806	907.77776	419.44445
378.22733	842.44442	405.11111
378.25662	835.88891	439.55553
378.28592	1086.66667	598.66667
378.31519	1061.44445	514.1111
378.34445	1111.99996	507.00002
378.37372	941.44448	491.44442
378.40298	894.11113	430.11111
378.43225	941.33333	409.66666
378.46152	1057.99996	584.66665
378.49078	1277.11111	637.44443
378.52005	1103.11109	537.44445
378.54929	984.66665	489.99999
378.57855	827.33331	424.33331
378.60779	1050.88893	584.55556
378.63702	1225.55553	625.22222
378.66626	1182.22218	613.88887
378.6955	1102.11115	540.44447
378.72473	1108.33329	607.99998
378.75394	1089.44446	528.77778
378.78317	1462.33329	903.33329
378.81241	1310.44448	752.44442
378.84161	1348.44444	713.77781
378.87082	1188.22218	729.88893
378.90002	1149.11115	566.77778
378.92923	1547	932.33337
378.95844	1315.00001	630.00001
378.98764	1248.88893	619.8889
379.01685	1100.33329	595.99998
379.04602	1337	755.33331
379.07523	1468.22218	779.22224
379.1044	1386.00004	730.99998
379.13358	1326.77782	658.77776
379.16278	1360.55555	688.22224

379.19196	1495.33329	748.99998
379.22113	1510.77782	776.44444
379.25027	1426.66667	667.66667
379.27945	1422.55555	773.22218
379.30862	1534.22222	855.88885
379.33777	1678.22222	873.88891
379.36691	1489.44449	848.44442
379.39609	1524.44444	849.11113
379.42523	1560.66671	807.66671
379.45438	1521.11111	775.7778
379.48352	1498.11115	762.44446
379.51263	1685.33329	910.33335
379.54178	1730.88885	888.22222
379.57092	1702.44449	939.44442
379.60004	1622.77778	799.77778
379.62915	1738.11115	909.11115
379.65829	1654.88885	825.55553
379.68741	1741.66663	1001.33338
379.71652	1691.77782	864.11113
379.74561	1812.22226	983.88889
379.77472	1839.33325	939.33337
379.80383	1727.55552	895.8889
379.83292	1749.33333	826.33333
379.86203	1767.88893	905.22218
379.89111	1804.77774	968.44442
379.9202	1890.00004	943.99998
379.94928	1858.11111	971.7778
379.97836	1917.44437	985.77774
380.00745	1941.33337	971.33331
380.03653	1990.33329	1075.66667
380.06561	2066.66671	1085.66665
380.09467	2059.33333	1021.99996
380.12372	2098.33325	1120.33338
380.1528	2230.11119	1138.44444
380.18185	2247.11111	1152.11111
380.21091	2583.55556	1356.55556
380.23996	2665.66675	1460
380.26901	3135.77769	1661.77782
380.29803	3616.00008	1883.33333
380.32709	4133.00016	2079.33341
380.35611	4433.44428	2273.11103
380.38516	4179.44444	2108.11107
380.41418	3171.66675	1467.66663
380.44321	2114.99992	988.00004
380.47223	1064.88884	423.88891

380.50125	447.11112	224.11111
380.53027	148.55555	60.22222
380.5593	125.88889	62.55555
380.58829	103.77779	79.1111
380.61731	62.33334	68.66666
380.6463	153.77778	113.44445
380.67529	96.55554	98.55554
380.70428	208.11112	104.44443
380.73328	87.88889	78.88889
380.76227	101.77778	82.77778
380.79126	153.55555	153.22221
380.82025	167.77778	104.44443
380.84921	259.55555	170.22222
380.8782	76.44444	50.11111
380.90717	211.66669	161.66669
380.93613	46.88891	4.88891
380.96509	90.11111	130.44445
380.99405	142.11111	55.44444
381.02301	169.33334	102.33334
381.05197	173.77777	69.11111
381.08093	227.8889	146.8889
381.10986	201.66668	135.66668
381.13882	164.22223	139.55556
381.16776	231.55555	159.88889
381.19669	82.22222	74.88889
381.22562	188	91.33333
381.25455	72.11111	14.44444
381.28348	177.88891	61.22222
381.31241	45.33333	51
381.34131	126.66667	107
381.37024	170.66669	121
381.39914	166.11112	115.44443
381.42807	55.77779	44.1111
381.45697	40.77778	5.44444
381.48587	37.66668	6.33333
381.51477	78.44444	30.1111
381.54367	87.11111	82.77778
381.57254	104.66667	97
381.60144	80.66668	81.66668
381.63031	208.88891	147.22222
381.65921	77.22222	60.55556
381.68808	117.66666	24.33333
381.71695	175.2222	127.88889
381.74582	181	72.33334
381.77469	154.55555	100.88889

381.80356	73.88888	16.88888
381.83243	100.44443	82.11112
381.86127	72.88891	48.55556
381.89014	76.44444	36.77778
381.91898	101.88888	89.55556
381.94781	127.88889	151.2222
381.97665	137.88889	12.88889
382.00549	115.11111	87.11111
382.03433	164.77778	61.44445
382.06317	231.2222	159.55554
382.09201	123.8889	50.55555
382.12082	13.88889	50.22222
382.14963	83.77778	50.77778
382.17847	17.77778	0
382.20728	81	0
382.23608	36.55556	65.55555
382.26489	152.44445	79.44444
382.2937	155.99999	119.99999
382.32251	175.44444	124.11112
382.35129	129.11111	119.77778
382.3801	159.11111	88.11111
382.40887	99.66668	17.00001
382.43765	168.22223	67.55556
382.46646	159.77778	121.11111
382.49524	89.88889	106.88889
382.52402	180	201
382.55276	160.55556	80.88888
382.58154	138.66667	120
382.61032	59	0
382.63907	161.77778	55.77778
382.66785	169.77777	134.77777
382.69659	50.77778	1.1111
382.72534	39.66667	0
382.75409	106.1111	138.1111
382.78284	124.11113	57.77779
382.81158	253.77778	260.11112
382.8403	124	55.33333
382.86905	171.99999	101.66668
382.89777	188.44444	107.11111
382.92651	183.33334	86.33333
382.95523	205.99999	152.66666
382.98395	102.55556	42.55555
383.01266	196.66665	184.66665
383.04138	134.11111	116.44444
383.0701	171.55555	83.22222

383.09879	116.33333	90.33333
383.1275	183.11112	102.44443
383.15619	228.11112	173.44445
383.18491	234.11112	166.77778
383.21359	136.22222	93.55556
383.24228	241.88889	176.88889
383.27097	142.22223	92.88889
383.29965	144	60.66667
383.32834	153	143.33334
383.35699	114.77777	12.11111
383.38568	168.22222	134.22222
383.41434	190	131
383.44299	197.33333	108.33333
383.47168	198.77779	101.77779
383.50034	62.88889	33.22223
383.52896	207.11111	117.77777
383.55762	142.55557	74.55557
383.58627	173.99999	117.33334
383.61493	126	77
383.64355	136.88889	41.55556
383.67218	265.11112	199.77775
383.70084	162.77781	173.44444
383.72946	110.22222	96.55556
383.75809	126.77778	40.77778
383.78671	206	164
383.81531	222.22224	217.88887
383.84393	158.55555	136.88889
383.87256	152.44444	59.11111
383.90115	170.88889	85.88889
383.92975	213.33332	112.99999
383.95837	245.44444	169.11111
383.98697	147.44444	50.44444
384.01556	148.33334	73.99999
384.04416	83.44445	0
384.07272	113.11111	86.44444
384.10132	190.33333	127.66667
384.12991	169.00001	151.33332
384.15848	181.33332	30.00001
384.18704	148.77778	108.77778
384.21561	164.22222	70.88889
384.24417	180.1111	143.44445
384.27274	230.11111	165.11111
384.3013	323.66667	152
384.32986	217.66667	106.33334
384.35843	248.11109	252.11109

384.38696	252.99998	176.00001
384.4155	153.11111	90.11111
384.44406	228.88889	151.88889
384.4726	157.66666	121.66667
384.50113	182.44445	72.1111
384.52966	172.55555	188.88889
384.5582	202.11111	35.77778
384.5867	160.88888	32.22222
384.61523	224.33333	149.99998
384.64374	276.88889	157.22223
384.67227	205.77778	130.77778
384.70078	236.22223	105.55556
384.72928	278.66669	172.66669
384.75778	200.77778	139.77778
384.78629	270	155
384.81479	393.88885	332.22223
384.84326	241.22221	117.22221
384.87177	164.22221	49.22223
384.90024	178.66667	94.66667
384.92871	314.88891	224.88888
384.95721	234.22222	49.8889
384.98569	255.22222	169.88889
385.01416	296.22223	185.8889
385.0426	262.66665	177.33334
385.07108	304.99998	179.00001
385.09955	203.44444	136.77777
385.12799	243.11111	121.44444
385.15646	274.66669	180.33331
385.18491	322.66667	180.00001
385.21335	373.66666	221.66667
385.24179	304.88889	157.22222
385.27023	261.11112	172.11112
385.29868	209.77779	108.44445
385.32709	277.11111	87.77778
385.35553	248	79.33333
385.38394	580.88889	469.2222
385.41238	396.44445	172.44445
385.4408	340.00001	147.33333
385.46921	475.33333	264.66668
385.49762	347.66667	174
385.52603	468.55555	271.22224
385.55444	263.77778	127.44444
385.58282	322	180.33333
385.61124	254.00001	129
385.63962	322	134

385.66803	353.44445	149.44445
385.69641	369.55556	146.55555
385.72479	287.11111	191.11111
385.75317	122.00001	39.99999
385.78156	223.33334	164
385.80991	179.44444	43.11111
385.83829	146.44445	97.77779
385.86664	190	55
385.89502	154.55555	121.22222
385.92337	112.11111	59.11111
385.95172	175.22222	102.55556
385.98007	238.66669	138
386.00842	279.22223	137.88889
386.03677	242.00001	80.33334
386.06512	369.88889	234.22223
386.09344	242.44443	127.77777
386.1218	133.55556	112.22222
386.15012	135.22223	100.88889
386.17844	136.99999	67.66666
386.20676	161.44444	28.44444
386.23508	165.33334	95.33334
386.2634	224.22223	170.88889
386.29172	154.66666	112.66666
386.32004	234.8889	119.22223
386.34833	235.66667	182.66667
386.37662	264.33333	110.66666
386.40494	351.66665	273.66665
386.43323	349.44442	188.1111
386.46152	208.77777	184.44444
386.48981	183.55555	100.22222
386.5181	109.88888	29.22222
386.54636	168.77778	60.77778
386.57465	174.22222	82.88889
386.60291	229.44444	53.77778
386.6312	264.1111	173.44445
386.65945	272.77776	141.44445
386.68771	251.11111	86.44445
386.71597	260.33333	148.66666
386.74423	218.33334	164.33333
386.77249	223.77777	100.77777
386.80075	238.22222	133.22221
386.82898	204.55556	109.88889
386.85724	190.44443	94.44445
386.88547	192.1111	98.44444
386.9137	274.44445	170.44444

386.94193	138.66667	100
386.97015	363.55557	251.55557
386.99838	438.55554	225.22223
387.02661	430.44444	313.1111
387.05481	288.8889	134.88888
387.08304	157.22222	139.55555
387.11124	228.77778	103.77778
387.13943	293.22221	214.22223
387.16766	205.55556	88.22222
387.19586	242.77776	183.77776
387.22406	203.1111	79.77777
387.25223	253.44445	117.77778
387.28043	408.55555	203.55556
387.30862	431.77778	183.77778
387.33679	332.88887	121.55556
387.36496	293.88889	164.22222
387.39316	284.44445	115.11111
387.42133	205.77778	180.11111
387.44949	194.22223	55.22223
387.47766	311.22223	189.22222
387.5058	314.7778	181.11111
387.53397	165.11111	113.44445
387.56213	189.55555	148.55556
387.59027	284	122.66667
387.61841	456.11111	265.11111
387.64655	350.1111	207.77779
387.67471	326.66666	117.66667
387.70282	218.77778	117.44445
387.73096	329.88888	198.55557
387.75909	298.22221	182.22221
387.78723	305.44444	142.1111
387.81534	285.22223	156.88889
387.84344	244.99999	175.66667
387.87158	364.00001	217.33332
387.89969	312	126.33331
387.9278	337.66667	128.66667
387.9559	327.22223	82.55556
387.98401	234.55555	114.88889
388.01208	323.11111	164.77777
388.04019	295.22223	114.55555
388.06827	306.77778	185.77778
388.09637	191.33334	104.99999
388.12445	375.2222	127.55554
388.15253	421.44444	215.77778
388.1806	469.44444	247.44444

388.20868	518.8889	294.22221
388.23672	546.55557	247.55557
388.2648	338.00001	183.66667
388.29285	301.11111	129.11111
388.32092	384.11109	189.44444
388.34897	385.55555	164.22223
388.37701	327.44444	192.77778
388.40506	371.55557	197.22222
388.43311	388.11113	174.77779
388.46115	511.22225	326.88888
388.4892	571.11111	288.77777
388.51721	518.33333	307.99999
388.54526	521.88888	333.88888
388.57327	380.55558	192.88889
388.60129	327.11112	138.77778
388.6293	310.33334	115.33333
388.65732	314.7778	212.44443
388.68533	411.33333	294.99999
388.71335	451	188.66667
388.74136	508.8889	251.55556
388.76935	512.11113	257.1111
388.79733	429.88888	221.88889
388.82535	365.77777	163.11111
388.85333	467.7778	264.11111
388.88132	334	142.66667
388.9093	426.2222	179.88889
388.93729	420.11111	173.44444
388.96524	474	270.66667
388.99323	576.22222	341.22222
389.02118	581	247.66669
389.04916	509.55557	235.88889
389.07712	372.22221	149.55555
389.10507	517.44443	281.11111
389.13303	451.55558	254.88889
389.16098	465.00001	277
389.18893	510.1111	192.44444
389.21686	591.77778	297.44444
389.24481	840.66669	394.66666
389.27274	931.00002	449.33333
389.30069	834.00002	414.33333
389.32861	767.66667	394.00001
389.35654	641.66667	327.66667
389.38446	463.77778	215.44444
389.41238	407.77778	208.44444
389.44028	250.33331	193.33331

389.4682	213.22223	90.22222
389.49609	319.11113	241.44444
389.52402	314	150.66667
389.55191	420.22221	280.8889
389.5798	279.77778	97.44444
389.6077	141.44444	99.77779
389.63559	156.88889	84.55555
389.66348	179.44443	142.11112
389.69135	212.22223	131.55554
389.71924	281.22221	198.55556
389.7471	284.88889	150.88889
389.77499	303.33333	197.66668
389.80286	256.88889	104.55556
389.83072	243.66667	149.66667
389.85858	113.88889	80.88889
389.88644	178.77777	145.77777
389.91428	100.00001	58.33332
389.94214	108.88889	47.55556
389.97	156.88889	36.22222
389.99783	261.22222	102.88888
390.02567	439.44442	268.77779
390.0535	411.88891	209.22222
390.08133	420.55556	198.88888
390.10916	248.55554	190.55554
390.13699	139.44446	68.77777
390.16483	120.44445	68.44445
390.19263	168.44445	84.77778
390.22046	283.55555	210.22221
390.24826	275.33331	139.33334
390.27606	221.55555	141.55555
390.30386	285.88889	167.22222
390.33167	168.66666	41.33333
390.35947	217.88889	86.22223
390.38727	175	100.33333
390.41504	200.33333	107.66667
390.44284	412.44446	215.44446
390.47061	289.88889	147.55555
390.49838	346.8889	214.55556
390.52618	255.88888	119.88889
390.55396	271.99999	151.33333
390.5817	148.11111	70.77777
390.60947	197.55556	130.55556
390.63724	174.77778	85.77778
390.66498	326.22224	257.22224
390.69275	347.11109	159.44443

390.72049	290.77778	101.77778
390.74823	378.00001	176.99999
390.77597	351.77776	159.77779
390.80371	290.33334	118.33333
390.83145	227.77778	150.77778
390.85919	187.77778	49.44445
390.88693	219.22221	203.22221
390.91464	289.8889	142.22223
390.94235	277.00001	138.66667
390.97009	317.22222	103.88889
390.9978	285.44442	172.44442
391.02551	226.11111	125.77777
391.05322	184.22222	81.8889
391.08093	231.33334	130
391.10861	402.44445	211.1111
391.13632	426.99999	238.33334
391.164	461.33332	206.66666
391.19171	436.77776	204.1111
391.21939	272.00001	193.99999
391.24707	379.88892	274.55554
391.27475	257.55556	126.88889
391.30243	318.66667	203
391.33008	453.22222	255.55557
391.35776	438	195.66669
391.38541	477.22222	263.88889
391.41309	324.22223	128.88889
391.44073	359.22224	193.55556
391.46838	296.44445	121.77779
391.49603	338.77778	245.11111
391.52368	367.44445	165.77778
391.55133	423.77777	306.77777
391.57895	418.88889	275.2222
391.6066	261.22221	156.88889
391.63422	202.22222	52.55556
391.66187	283.55557	215.55557
391.68948	224.11111	56.11111
391.7171	257.11111	155.77777
391.74472	391.22222	251.22222
391.77234	490.33331	303.99999
391.79996	351.44444	137.77777
391.82755	351.11112	169.44445
391.85516	322.55555	240.22223
391.88275	276.66667	194.33334
391.91034	414.8889	224.22221
391.93793	316.55556	176.55556

391.96552	322	183.33333
391.9931	309.33333	178.66668
392.02069	343.44444	230.11112
392.04828	353.55555	272.55555
392.07584	366.99999	200.66666
392.10342	438.88891	212.22222
392.13098	449.33333	171.66668
392.15854	443.22223	193.88889
392.1861	364.33334	149.33333
392.21365	447.88888	263.55556
392.24121	355.33333	245.66665
392.26877	478.33333	313.66668
392.2963	386.22222	188.88889
392.32385	399.88889	234.55555
392.35138	325.88888	118.8889
392.37891	312.1111	200.77777
392.40643	411.88891	222.55556
392.43396	424	276
392.46149	619.55558	365.2222
392.48901	565.11112	280.77778
392.51654	484.44445	186.44445
392.54404	431.66667	211.00001
392.57156	326.88889	128.88889
392.59906	395.22222	264.22222
392.62656	405.33333	171.66667
392.65405	419.22221	208.55558
392.68155	463.44443	224.77777
392.70905	412.22222	213.88889
392.73654	491.55555	289.22221
392.76401	476.77778	317.44446
392.7915	582.11109	208.11112
392.81897	476.44445	157.44445
392.84644	552.77777	286.77778
392.8739	477.00002	250.33333
392.90137	476.33332	271.66666
392.92883	461.33333	311.33333
392.9563	496.8889	235.55555
392.98376	491.55558	293.88889
393.0112	463.77778	226.77778
393.03864	605.11111	293.77778
393.0661	551.66667	248.33334
393.09354	527.33335	277.33332
393.12097	676.44442	292.11111
393.14841	627.11109	367.11112
393.17584	572.44443	247.77778

393.20325	457.1111	211.77777
393.23068	497.88889	215.22223
393.25809	468.66668	349.00001
393.28552	475.55557	236.22222
393.31293	472.99999	245.99999
393.34033	599.88891	323.88888
393.36774	582.55555	292.22223
393.39514	638.77778	302.77778
393.42255	863.00004	515.33335
393.44992	658.00002	312.99999
393.47733	640.99998	366.00001
393.5047	529.88887	279.8889
393.53207	402.00001	244.33334
393.55945	537.00003	294.66665
393.58682	383.55555	203.55555
393.6142	539.77776	257.11112
393.64157	562.11111	351.11111
393.66895	640.7778	292.11111
393.69629	722.55553	383.22222
393.72366	711.55556	287.55556
393.75101	812.22218	442.55555
393.77835	723.55555	404.55555
393.80569	604.33333	246.33333
393.83304	756.77778	448.44447
393.86038	613.66665	333.00002
393.88773	585.55557	287.22223
393.91504	743.88891	381.22222
393.94238	775.44443	400.77779
393.9697	972.22226	553.55551
393.99704	1053.77774	605.11111
394.02435	1051.66664	485.66666
394.05167	1185.88889	610.88889
394.07898	1113.22222	573.88891
394.10626	1023.77782	476.44445
394.13358	1236.55555	625.55555
394.16086	427.11112	1981.44462
394.18817	917.11107	567.44445
394.21545	691.55551	330.88889
394.24274	577.88889	319.55556
394.27002	428.1111	145.77778
394.2973	435.99998	324.99998
394.32458	259.22221	79.22223
394.35187	217.33334	220.66665
394.37912	61.22222	0
394.4064	33.77778	76.77778

394.43365	35	8
394.46091	158.22223	88.88889
394.48819	143.44445	87.44444
394.51544	245	159
394.54266	280.00001	105.66667
394.56992	412.33333	205.66666
394.59717	143.22222	23.55556
394.62439	206.77777	103.11111
394.65164	80.55556	51.88889
394.67886	102.88889	139.22222
394.70609	153	79.33333
394.73331	175.44446	157.11111
394.76053	159.44445	105.44445
394.78775	211.22222	171.22222
394.81494	322.99999	197
394.84216	325.77777	220.11108
394.86935	295.44444	105.11112
394.89658	191	135
394.92377	145.55555	46.22222
394.95096	87.55556	90.22222
394.97815	153.66666	162.33334
395.00534	263.33333	43.33333
395.0325	171	89.66666
395.05969	271.99998	177.00001
395.08688	335.22222	137.88888
395.11404	249.22222	85.88889
395.1412	223.77777	99.11111
395.16837	105.44445	97.1111
395.19553	157.11111	103.77777
395.22269	45.77778	20.77778
395.24985	92.88889	48.55556
395.27698	229.55558	147.8889
395.30414	303.33335	197.00001
395.33127	365.66667	224.33333
395.35843	384.77779	186.11111
395.38556	305.8889	215.22221
395.41269	272.8889	156.8889
395.43982	237.55556	215.55555
395.46695	86.44444	66.44444
395.49405	118.77776	115.77776
395.52118	207.1111	115.1111
395.54828	244.88889	166.22222
395.57541	289.33334	95.66667
395.60251	466.1111	299.1111
395.62961	396.55552	196.8889

395.65671	283.55556	153.55555
395.68381	177.44445	79.11111
395.71088	234.55557	187.55557
395.73798	145.55555	64.88888
395.76508	124.33333	62.33333
395.79214	315.22223	160.88889
395.81921	299.22221	216.55556
395.84628	416.55557	246.88889
395.87335	284.1111	123.44444
395.90042	277.55557	95.22222
395.92749	140.66666	98
395.95456	109.44444	79.44444
395.9816	120.88889	121.55556
396.00867	274.33333	196
396.03571	326.88888	165.22222
396.06274	449.66667	242.66667
396.08978	451.55556	258.88889
396.11682	326.88889	231.22222
396.14386	303.66667	141.99999
396.17087	225.66668	154.66668
396.19791	107.77778	61.77778
396.22495	188.11111	96.11111
396.25195	238.1111	152.77779
396.27896	372.66665	183.66665
396.30597	407.44445	221.77778
396.33298	322.22223	153.88889
396.35999	267.22222	165.22222
396.38699	263.11111	160.77777
396.41397	194.88889	126.55555
396.44098	216.66667	68
396.46796	321.66669	169.66666
396.49493	432.11111	194.77777
396.52194	368.22222	184.88889
396.54892	498.88887	219.22221
396.57587	372.77779	283.44445
396.60284	192.77778	70.1111
396.62982	125.11111	40.44444
396.65677	190.11111	80.1111
396.68375	320.22224	207.55555
396.71069	469.11111	295.44446
396.73764	465.66666	224
396.76459	311.22222	136.55555
396.79153	373.22223	184.55555
396.81848	292	174.66666
396.84543	328.33333	223

396.87234	228.66666	116
396.89929	433.66669	263
396.92621	425.8889	204.55556
396.95313	402.44443	291.11111
396.98004	421.44447	233.77778
397.00696	369.66666	282.66666
397.03387	325.88891	118.88888
397.06079	214.22222	177.55556
397.08771	339.88888	204.55555
397.11459	466.33335	280.00001
397.14148	405.66668	153.66666
397.1684	552.77776	314.44445
397.19528	517.88888	293.55555
397.22217	362.55556	222.55556
397.24905	401.33332	245.00001
397.27594	355.11111	194.11111
397.3028	437.1111	251.11112
397.32968	658.77778	410.77778
397.35654	460.55558	232.88889
397.38339	386.66667	208.66667
397.41028	431.44443	298.7778
397.43713	355.88887	280.22221
397.46399	324.77777	115.77779
397.49081	402.22222	217.22222
397.51767	520.66667	344.33332
397.54453	500.99998	298.00001
397.57135	442.8889	257.55556
397.59818	375.88889	247.88889
397.62503	369.66666	161
397.65186	447.77778	297.11112
397.67868	524	331.33331
397.70547	368.77778	188.44444
397.7323	468.55556	226.22223
397.75912	412.7778	225.44445
397.78592	440.44447	305.44447
397.81274	410.33333	266
397.83954	510.77777	205.11111
397.86633	563.99998	348.33335
397.89313	508.11109	217.77778
397.91992	510.00001	281.99999
397.94672	486.55556	168.88888
397.97348	558.7778	342.44443
398.00027	434.22224	221.22221
398.02704	574.55557	306.88889
398.0538	473.22222	211.88889

398.0806	438.66669	258.66666
398.10736	508.1111	297.77779
398.13412	516.55554	241.55555
398.16086	461.77779	251.77778
398.18762	488.66669	255.33331
398.21439	676.22222	394.22222
398.24112	624.77779	320.44444
398.26785	579.22222	250.22222
398.29462	523.88891	293.22222
398.32135	480.11111	192.77777
398.34808	539.55555	228.55555
398.37479	513.44443	251.77778
398.40152	436.55556	204.22223
398.42825	398.22222	186.22222
398.45496	600.11109	355.11112
398.48169	515.33333	228.33333
398.50839	513.7778	273.7778
398.5351	550.55558	273.55554
398.5618	648.55554	374.88888
398.5885	704.66666	435.33332
398.61517	663.66669	357.33334
398.64188	753.7778	443.7778
398.66858	552.33332	221.66667
398.69525	519.33333	282.33333
398.72192	566.55558	274.55555
398.7486	587.33335	334.33332
398.77527	555.55556	310.88888
398.80194	585.44442	257.44444
398.82861	678.88893	290.22224
398.85529	721.44445	502.11113
398.88193	630	307
398.9086	676.44442	364.44446
398.93524	596.66667	343.66667
398.96188	676.2222	264.22223
398.98853	670.33335	335.00001
399.01517	600.88888	251.55556
399.04181	622.33333	325.33333
399.06845	607.66665	286.66668
399.09506	649.88889	308.88889
399.1217	788.44446	313.44443
399.14832	660.44446	381.44446
399.17493	871.77773	513.44448
399.20154	817.44447	494.44447
399.22815	871.44442	361.44445
399.25476	822.22222	367.88888

399.28137	814.77782	493.77776
399.30795	692.88889	307.22222
399.33456	738.55558	435.88889
399.36115	705.88891	408.55554
399.38773	755.55558	336.55554
399.41431	767.66669	426.33334
399.44089	888.11113	444.44444
399.46747	807.33333	357.33333
399.49405	1058	569.66669
399.52063	1179.1111	681.77779
399.54718	1149.99996	634.00002
399.57376	1307.77774	710.11111
399.60031	1376.44441	686.44447
399.62686	1456.88889	730.88889
399.65341	1410.44449	631.7778
399.67996	1272.66662	598
399.70651	1095	454.66668
399.73303	836.44445	356.77778
399.75958	740.11113	354.44444
399.7861	463.33335	219.66667
399.81265	407.44448	222.1111
399.83917	302.33333	171.33333
399.86569	171.33333	93
399.89221	198.88889	176.22223
399.91873	181.66667	118.33333
399.94522	71.22222	91.22222
399.97174	61.11111	139.44444
399.99823	133.88889	95.55556
400.02475	22.22221	24.8889
400.05124	106.11111	56.11111
400.07773	81	108.33333
400.10422	171.77778	112.44445
400.13071	295.55557	238.55557
400.15717	160.33334	36.66667
400.18365	125.77778	100.1111
400.21011	159.22225	102.22225
400.2366	98.66667	0
400.26306	60.77778	161.11111
400.28952	47	43
400.31598	104.33333	97
400.34244	59.88891	59.22222
400.3689	150.44445	86.77778
400.39532	158.11111	96.77777
400.42178	165.88889	97.55556
400.44821	200.88889	124.55557

400.47467	115.77778	52.77778
400.5011	119.33333	97.33333
400.52753	59.55555	68.22223
400.55396	9.77778	29.11111
400.58035	0	0
400.60678	179.44444	117.11111
400.63321	88.66668	43.99999
400.65961	198.22222	160.22222
400.686	197	126
400.7124	202.55556	34.55555
400.7388	133.22222	99.22222
400.7652	200.44444	101.77779
400.7916	119.11111	82.11111
400.81799	122.11111	101.44445
400.84436	116.88889	87.88889
400.87076	20.66666	53.33334
400.89713	145.33333	161.66667
400.92349	178.66666	139.33333
400.94986	173.77777	127.11111
400.97623	281.33332	125.00001
401.00259	177.66668	79.66667
401.02896	176.22223	51.8889
401.0553	215.33334	174.66666
401.08167	94.55556	52.55555
401.108	112.33333	87.66667
401.13434	93.11111	30.44445
401.16071	86.22222	65.22222
401.18701	153.66668	100.99999
401.21335	149	134.66667
401.23969	220.33333	126.66667
401.26602	220.77777	173.11111
401.29233	280.55557	188.2222
401.31863	166.88889	87.88889
401.34497	61.88889	57.22222
401.37128	90.88888	103.55556
401.39758	55.77777	163.44446
401.42389	98.55555	58.55555
401.45016	150.22223	42.88889
401.47647	260.00001	190.66667
401.50278	353.11112	209.77778
401.52905	283	167.33333
401.55533	139.11111	36.11111
401.5816	81.33333	20.33333
401.60788	169.11112	149.77778
401.63416	85.66667	27.66667

401.66043	0	0
401.68671	125.99999	119.99999
401.71295	254.88889	149.88889
401.73923	239.33334	96
401.76547	295.55555	107.22223
401.79172	348.22222	225.55553
401.81796	261.22221	135.55555
401.84421	124.99999	55.66666
401.87045	95.77777	36.77777
401.8967	45.33334	61.66667
401.92291	112	11.33333
401.94916	95.33334	90.66667
401.97537	261.77778	134.77778
402.00159	353.44443	239.11111
402.0278	337.77779	171.44444
402.05402	193	107
402.08023	178.77778	82.44444
402.10645	155.77777	91.77777
402.13263	120.44443	58.44443
402.15884	83.44444	64.11111
402.18503	133.66667	100.99999
402.21121	263.99999	161.99999
402.2374	327.77779	234.1111
402.26358	303.88889	173.55556
402.28976	217.66666	65.66667
402.31595	138.33333	83
402.3421	153.33331	101.33331
402.36829	128.11111	79.44445
402.39444	90.55555	56.55556
402.42059	274.44447	216.44447
402.44675	258.2222	178.22223
402.4729	334.00001	273.66666
402.49905	319.22221	190.8889
402.52521	250.33334	135
402.55136	193.55555	101.8889
402.57748	139.33333	45.33333
402.60364	144.88889	106.22222
402.62976	246.44443	262.44443
402.65588	290.8889	167.22221
402.68201	248.77778	155.44445
402.70813	501.22221	204.22222
402.73425	323.66667	214.33332
402.76035	217.44445	69.11111
402.78647	386.1111	304.44442
402.81256	199.22222	128.55555

402.83865	134.33333	176.33333
402.86478	256.99999	202.66668
402.89087	307.55555	210.22221
402.91693	332.11111	205.44444
402.94302	370.77777	253.77777
402.96912	370	253.66669
402.99518	206.11112	123.11112
403.02127	149.44445	82.44445
403.04733	215.33335	129.33332
403.07339	305.88891	182.55556
403.09946	388.22222	279.22222
403.12552	314.22223	145.88889
403.15158	343.33336	205.33333
403.17764	337.88892	205.55554
403.20367	338.55554	247.22223
403.22974	334.44444	178.44444
403.25577	257.77778	193.44444
403.2818	247.44443	129.44443
403.30783	379.33332	227.33333
403.33386	403.11111	209.77778
403.35989	365.44444	239.77779
403.38593	322.00003	183.66668
403.41193	243.22222	90.55555
403.43796	238.66667	113
403.46396	199.55556	97.88889
403.48996	351.11111	167.44444
403.51596	325.66667	210.66667
403.54196	306.44445	160.44445
403.56796	262.77778	124.77778
403.59396	209.1111	59.44444
403.61993	306.55555	154.55555
403.64594	401.33333	242.66666
403.67191	327.77778	243.44443
403.69788	373.1111	133.11111
403.72385	776.22225	629.88888
403.74982	288.1111	132.44444
403.77579	413.33334	216
403.80176	449.11111	274.7778
403.82773	331.77778	194.11111
403.85367	371.99999	177
403.87961	341.22223	207.55554
403.90558	434.22222	226.88889
403.93152	344.77777	171.44444
403.95746	302.00001	136.66668
403.9834	421.11111	308.7778

404.00931	388.99999	150
404.03525	483.66667	264.66667
404.06116	439.22222	226.55556
404.0871	413.55555	226.55555
404.11301	450.44443	267.77777
404.13892	446.66668	187.33333
404.16483	356.33334	233.33333
404.19073	391.55555	217.88889
404.21664	351	192.66667
404.24252	393.22221	277.55556
404.26843	264.11112	165.77778
404.29431	436.55557	317.22222
404.32022	442.77777	219.77777
404.3461	433.66668	298.66666
404.37198	441.55556	254.55555
404.39786	474.00002	278.33333
404.42371	384.88889	216.22222
404.44958	500.00002	306.33333
404.47546	480.88891	164.88889
404.50131	539.77778	333.77778
404.52716	437.66668	219.66667
404.55301	446.33334	275.66666
404.57886	505.33335	265.33332
404.60471	397.11111	251.11111
404.63055	418.33332	168.33333
404.6564	459.77777	256.77777
404.68222	408.33334	217.66667
404.70807	398.66668	238.33333
404.73389	350.55556	171.55556
404.7597	467.22221	234.55556
404.78552	514.55556	311.8889
404.81134	468.77779	249.11112
404.83716	523.44444	319.77777
404.86298	372.55555	196.55555
404.88876	532.88891	311.88888
404.91458	533.66668	331.33333
404.94037	519.22221	260.55556
404.96616	445.11111	193.44444
404.99194	511.88888	266.55555
405.01773	471.44445	249.1111
405.04352	424.66668	180
405.06931	505.66667	289.99999
405.09506	479.22221	229.88888
405.12085	649.3333	447.66668
405.14661	514	269.66667

405.17236	563.77776	323.44444
405.19812	521.11109	232.77778
405.22388	596.55557	387.88889
405.24963	493.66666	194
405.27539	610.88887	272.8889
405.30112	590.33331	285.33334
405.32687	637.66669	417.33331
405.3526	497	191
405.37833	661.66667	439.99998
405.40405	550.88889	267.55556
405.42978	540.33334	290.66667
405.45551	679	374.66669
405.48123	575.44446	317.11111
405.50693	557.1111	270.77778
405.53265	584.55558	374.22223
405.55835	740.44445	379.1111
405.58405	723.44444	434.1111
405.60974	735.11113	359.1111
405.63544	786.55558	341.88889
405.66113	944.66669	446
405.68683	912.55556	479.8889
405.71249	986.7778	473.11111
405.73819	1164.22222	590.88891
405.76385	1190.88885	553.88888
405.78952	1155.11107	672.44444
405.81522	850.33335	433.33332
405.84085	718.55556	377.8889
405.86652	414.00001	126.66667
405.89218	215.22223	120.88889
405.91785	206.11111	140.44445
405.94348	220.33333	234.66665
405.96912	120.33333	39.66667
405.99478	110.33333	105
406.02042	106	93.66667
406.04605	120.88888	95.55556
406.07169	58.55555	6.88889
406.09729	131	128
406.12292	73.88889	34.55555
406.14853	131.11111	92.44444
406.17416	79	5
406.19977	75.55556	0
406.22537	55	0
406.25098	112.88889	77.88889
406.27658	163.55555	94.22223
406.30219	139	68.66667

406.32776	88.55555	41.88889
406.35336	82.66667	104.66667
406.37894	124.33333	51.66667
406.40451	52.66667	55.33333
406.43011	53.77778	94.77778
406.45569	59.88889	44.88889
406.48123	107.77778	94.44443
406.50681	110	88.66667
406.53238	78.88889	19.55556
406.55792	69.22222	103.88889
406.5835	82.55555	26.22222
406.60904	70.44444	61.44444
406.63458	126.88889	7.55556
406.66013	66.44445	53.44445
406.68567	168.55555	58.88889
406.71121	126.22223	110.22223
406.73672	354.66671	357.99996
406.76227	86.88889	7.55556
406.78778	137.22222	75.22222
406.81329	65.33333	35.33333
406.83884	103.77778	76.44444
406.86435	147.22222	151.55556
406.88983	91.66667	37
406.91534	119	26.66667
406.94086	81.55555	39.55555
406.96634	84	54.33334
406.99185	165.77779	147.1111
407.01733	155.44444	88.77778
407.04282	134.88888	112.55556
407.0683	91.66667	70.33333
407.09378	131	42.33333
407.11926	50.22222	81.88889
407.14471	92.33334	19.66667
407.1702	181.66666	174.33334
407.19565	180.33334	143.99999
407.22113	57.11111	54.11111
407.24658	176.33333	96
407.27203	104.33333	52.66666
407.29749	126	61.33333
407.32291	109.77777	71.11112
407.34836	162.77778	81.77778
407.37381	82.33333	26
407.39923	90.66667	0
407.42465	104.33333	81
407.45007	105.44445	27.1111

407.47549	83.11111	85.77778
407.50092	78.33333	5
407.52634	72.88888	44.88889
407.55176	173.44445	211.44445
407.57715	129.33333	47.66667
407.60257	179.33334	135
407.62796	165.11111	168.11111
407.65335	82.66666	55.66667
407.67874	136.11111	67.11111
407.70413	50	6.33334
407.72952	156.33334	85.33333
407.75488	53.22222	0
407.78027	122	104.66667
407.80563	162	90.66667
407.83102	35.22222	35.55556
407.85638	100.44445	16.44444
407.88174	98.88889	33.55556
407.9071	153.55556	67.88889
407.93243	104	51.33333
407.95779	136.66667	16
407.98315	70.77778	48.77778
408.00848	84	38.33334
408.03381	129.44445	117.44445
408.05914	92.88889	47.22222
408.08447	143.1111	147.1111
408.1098	173.22222	110.88889
408.13513	69.55555	67.55556
408.16046	104.22222	0
408.18576	183.33333	117.33333
408.21109	157.55557	69.8889
408.23639	152.44444	115.44444
408.26169	91.11111	34.11111
408.28699	146.66667	91
408.31229	184.66667	115.66667
408.33759	190.33334	143.66667
408.36285	84.77777	95.44445
408.38815	243.2222	144.22223
408.41342	113.11111	26.77777
408.43872	149.44444	85.77778
408.46399	112.55555	53.55556
408.48926	186.33333	181.99999
408.51453	156	91.66667
408.53976	79.77778	37.11111
408.56503	211.1111	133.77778
408.5903	142	72.33333

408.61554	247.44444	169.44444
408.64078	161.44444	77.44444
408.66602	149.55556	70.88889
408.69125	177.66667	105.00001
408.71649	115.55555	31.88889
408.74173	153.11111	79.11111
408.76697	37.77778	39.11111
408.79218	174.11112	142.77778
408.81741	165.33333	90
408.84262	184.33332	51.99999
408.86783	219.11111	88.11111
408.89304	106.88889	83.55556
408.91824	218.11111	82.77778
408.94345	143.77778	147.44444
408.96863	153.22223	91.22223
408.99384	181.66667	129.66667
409.01901	210.44444	108.77778
409.04422	233.77779	214.77779
409.0694	299.22223	128.88889
409.09457	189.77778	111.77778
409.11975	179.44444	90.77777
409.1449	226.33334	44.66667
409.17007	270.55554	166.88889
409.19525	260	83
409.2204	230.44445	117.11111
409.24554	133.66667	70
409.27069	143.22223	60.55556
409.29584	248.77779	235.77779
409.32098	186.77777	126.44444
409.34613	153.77778	113.77778
409.37128	161.33333	93.66667
409.39639	128	70.66667
409.42154	88.33334	0
409.44666	123	90.66667
409.47177	146.22222	87.55555
409.49689	66.77778	68.44444
409.522	31.8889	0
409.54712	0	3.33333
409.5722	20.66667	5.66667
409.59732	65.33333	67.33333
409.62241	35.33333	16
409.64749	154.77779	187.44445
409.67261	63.44445	10.11112
409.69769	35.22222	2.22222
409.72278	113.22222	84.55556

409.74783	38.66667	69.33333
409.77292	121.88889	93.22222
409.79797	88.77777	94.44445
409.82306	156.77778	58.11111
409.84811	117.33334	89.33334
409.87317	170.88888	90.55556
409.89822	82	83
409.92328	176.33333	105.33334
409.94833	121.77778	80.44445
409.97336	58.33333	31.33333
409.99841	93.55556	106.55556
410.02344	114.88889	110.55556
410.04849	48.33333	67.66667
410.07352	188.22221	113.88888
410.09854	150.88889	63.55556
410.12357	91.22223	32.22222
410.14856	28.22222	88.22222
410.17358	148.33334	101
410.19858	86	21.33333
410.2236	221.22221	150.22221
410.2486	56.22222	45.55556
410.27359	167.55555	94.55556
410.29858	54.66667	77
410.32358	93.33334	74.66666
410.34857	95.88889	28.55556
410.37354	42.55555	20.22222
410.39853	63.77778	59.44444
410.42349	106.77779	31.11111
410.44846	84	64
410.47345	82.33333	59
410.49838	79.66667	77
410.52335	74.1111	15.11111
410.54831	36.88889	65.22223
410.57327	188.22222	147.55556
410.59821	74.55555	91.22222
410.62317	86.66666	25.66667
410.6481	156.77778	96.77778
410.67303	165.11112	129.77778
410.69797	76.33334	79.66667
410.7229	149	96
410.7478	50.44444	15.11111
410.77274	135.22222	94.55556
410.79764	156.66666	119.66666
410.82257	110	95.33333
410.84747	75.55556	154.55556

410.87238	148.88889	67.22222
410.89728	134.44444	101.77778
410.92218	88.11111	28.44445
410.94708	139.66666	89
410.97195	203.11111	118.44444
410.99686	270.88888	181.55557
411.02173	124.11111	64.77778
411.0466	139.66667	125.66667
411.07147	46.11111	0
411.09634	127.55556	74.22223
411.12122	117.44445	110.44445
411.14609	121.88888	49.88889
411.17093	99.77778	67.44445
411.1958	105.77778	130.11111
411.22064	114.55555	36.22222
411.24548	107.77778	106.44444
411.27032	206.22222	139.55557
411.29517	132.55554	121.55554
411.32001	111.11111	86.77778
411.34485	160.22222	73.88889
411.36966	214.8889	93.22221
411.3945	185.33332	95.33334
411.41931	233.77778	97.44444
411.44412	244.33333	188.66666
411.46893	91.44444	63.11111
411.49374	135.33333	70.66666
411.51855	78.44445	48.11111
411.54337	151.77778	75.11111
411.56815	129.55556	69.22222
411.59296	157.55555	160.55555
411.61774	100.33333	50.66667
411.64252	114.22222	66.55555
411.6673	69.11111	19.11111
411.69208	110.33333	127.33333
411.71686	247.77778	187.11109
411.74164	158.88889	129.22222
411.76639	217.55555	78.22223
411.79117	199.22222	139.88889
411.81592	221.77778	72.44445
411.84067	112.77778	106.44445
411.86542	30.22223	0
411.89017	150.33333	123
411.91492	208.33333	131.99999
411.93967	166.11112	98.77778
411.96439	97.77778	21.44444

411.98914	221.11111	138.77777
412.01385	164.33334	100.33333
412.03857	101.66667	0
412.06329	172.11111	80.44444
412.08801	175.77777	84.44445
412.11273	182.44445	105.1111
412.13742	233.66667	147.33333
412.16214	232.33333	116.33333
412.18683	222.55556	172.22222
412.21155	159.11112	55.11111
412.23624	186.11111	107.77778
412.26093	104.44444	56.44444
412.28561	210.55557	109.22222
412.31027	246.55556	119.88889
412.33496	90.22222	89.88889
412.35962	129.77778	109.11111
412.38431	173.33333	126.66668
412.40897	228.33335	136.66667
412.43362	168.66667	83
412.45828	218	100.33333
412.48294	276	87.66667
412.5076	239.88888	122.55555
412.53223	82.44445	47.44444
412.55688	135.55556	81.22222
412.58151	152.22222	91.55556
412.60617	92.55556	74.88889
412.63107	35.22222	11.22222
412.66296	0	0
412.69482	30.44446	11.11111
412.72668	0	0
412.75858	0	0
412.79044	0	0
412.8223	207.44444	131.44444
412.85413	111.44448	46.1111
412.88599	16.77778	36.77778
412.91785	34.77779	15.11111
412.94968	154	72.66666
412.98154	18.99998	0
413.01337	46.00001	0
413.0452	63.99999	10.99999
413.07703	122.66669	133
413.10886	45.22222	40.55556
413.14069	24.55556	0
413.17252	135.66667	77.33333
413.20432	54.33334	0

413.23615	132.44448	35.1111
413.26794	73.44444	0
413.29977	0	0
413.33157	92.33332	20.00001
413.36337	39.22222	0
413.39517	162.33333	72.66667
413.42697	77.22223	36.88889
413.45877	52.8889	0
413.49057	145.55558	21.8889
413.52234	87.44446	0
413.55414	118.11111	81.11111
413.58591	98.33331	23.66666
413.61768	95.77777	62.44446
413.64948	100.77776	0
413.68124	36.00002	34.33333
413.71301	105.88891	13.22222
413.74478	59.1111	0
413.77652	105.44445	25.77776
413.80829	91.44443	0
413.84006	217.22223	65.88889
413.8718	163.22224	44.22224
413.90353	179.99999	28.99999
413.9353	189.88889	42.55556
413.96704	242.22223	75.55554
413.99878	178.55558	90.22221
