# Supplementary material for: Nitrogen fixation efficiency enhancement using plasma jet: Impact of grounded electrode
Source: PLoS One. 2026 Sep 1;21(9):e0346219. doi: 10.1371/journal.pone.0346219 (PMC13533402; doi:10.1371/journal.pone.0346219)
Supplement: Supplementary file 3 [file pone.0346219.s003.pdf]

Data of Fig 4(a).

Gas composition	Rotational temperature, T_r (K)	Error (±)	Vibrational temperature, T_v (K)	Error (±)	Excitation temperature, T_e (K)	Error (±)	Electron density, n_e (cm^{-3})	Error (±)
N2	1112.23395	22.3171	3742.8187	44.59545	9231.7386	508.8935	1.08975E16	2.8261E14
Air	1799.15393	15.61867	4474.0372	40.63496	10097.43485	514.52231	1.16238E16	2.15087E14
Air:O2::5:3	1849.85019	16.94655	4800.06002	65.0246	10959.96449	499.07207	1.36787E16	1.66751E14
Air:O2::1:3	1901.61656	63.00499	5466.63443	231.7447	11382.52185	434.80067	1.06617E16	1.36711E14
O2	1092.4658	318.0614	3596.22124	7.17E+03	12213.21314	585.33386	8.45757E15	2.3127E14

Data of Fig 4(b).

Gas flow rate (L/min)	Rotational temperature, T_r (K)	Error (±)	Vibrational temperature, T_v (K)	Error (±)	Excitation temperature, T_e (K)	Error (±)	Electron density, n_e (cm^{-3})	Error (±)
2	1901.40914	32.15495	4601.83053	89.41886	10332.75906	384.22363	1.18093E16	5.13052E14
3	1938.92206	15.29056	4736.75547	44.15661	10959.96449	698.3423	1.36787E16	1.50465E14
4	1849.85019	16.94655	4800.06002	65.0246	11327.79153	599.07207	1.14154E16	1.66751E14
5	1822.77943	26.72164	5034.59584	84.37695	12023.89992	643.33282	1.07821E16	1.77942E14
6	1800.56437	32.6243	4825.93837	94.48488	12527.20962	563.64802	8.89483E15	2.77516E14

Data of Fig 4(c).

Electrode configuration	Rotational temperature, T_r (K)	Error (±)	Vibrational temperature, T_v (K)	Error (±)	Excitation temperature, T_e (K)	Error (±)	Electron density, n_e (cm^{-3})	Error (±)
Floating water ground electrode	1752.20885	45.84407	4753.38099	133.6951	10381.59741	447.59472	1.11298E16	1.84566E14
Fixed ground electrode	1938.92206	15.29056	4736.75547	44.15661	10959.96449	499.07207	1.36787E16	1.66751E14
