# Supplementary material for: Nitrogen fixation efficiency enhancement using plasma jet: Impact of grounded electrode
Source: PLoS One. 2026 Sep 1;21(9):e0346219. doi: 10.1371/journal.pone.0346219 (PMC13533402; doi:10.1371/journal.pone.0346219)
Supplement: Supplementary file 4 [file pone.0346219.s004.pdf]

Data of fig 5(a).

Gas composition	NO_2^- Concentration (mg/L)	Error (±)	NO_3^- Concentration (mg/L)	Error (±)	H_2O_2 Concentration (mg/L)	Error (±)	O_3 Concentration (mg/L)	Error (±)	pH	Error (±)
N_2	17.292	0.4323	75.498	1.73645	26.361	0.65903	1.6	0.0272	3.6	0.144
Air	39.969	1.79861	210.123	8.40492	85.558	1.96783	0.1	0.0023	3	0.069
Air:O_2::5:3	73.702	1.10553	275.219	8.25657	123.434	4.93736	0.1	0.004	2.9	0.0667
Air:O_2::1:3	42.079	1.47277	194.789	3.31141	51.107	1.53321	0.4	0.012	3	0.051
O_2	0.106	0.00212	0.306	0.00765	1	0.017	0.1	0.0025	7.3	0.1825

Data of fig 5(b).

Gas flow rate (L/min)	NO_2^- Concentration (mg/L)	Error (±)	NO_3^- Concentration (mg/L)	Error (±)	H_2O_2 Concentration (mg/L)	Error (±)	O_3 Concentration (mg/L)	Error (±)	pH	Error (±)
2	73.379	1.83448	270.861	8.12583	149.597	2.54315	0.1	0.0023	2.8	0.0644
3	81.889	3.68501	298.009	5.06615	182.827	4.20502	0.1	0.004	2.9	0.0493
4	73.702	1.10553	275.219	6.88048	123.434	4.93736	0.1	0.003	2.9	0.0725
5	62.347	2.80562	242.544	5.57851	64.73	1.9419	0.1	0.004	2.8	0.084
6	54.475	0.81713	227.081	9.08324	60.996	1.03693	0.3	0.009	2.8	0.07
