# Supplementary material for: Nitrogen fixation efficiency enhancement using plasma jet: Impact of grounded electrode
Source: PLoS One. 2026 Sep 1;21(9):e0346219. doi: 10.1371/journal.pone.0346219 (PMC13533402; doi:10.1371/journal.pone.0346219)
Supplement: Supplementary file 5 [file pone.0346219.s005.pdf]

Data of fig 6.

Treatment Time (min)	Floating water ground electrode									
	NO_3^- Concentration (mg/L)	Error (±)	NO_2^- Concentration (mg/L)	Error (±)	H_2O_2 Concentration (mg/L)	Error (±)	O_3 Concentration (mg/L)	Error (±)	pH	Error (±)
10	111.635	2.08157	4.311	0.14661	1.831	0.0613	0.35	0.02944	2.9	0.08
20	158.891	5.08501	53.732	1.52489	7.887	0.2141	0.4	0.02357	2.6	0.06
30	232.47	9.19179	0.661	0.03027	17.815	0.64579	0.18	0.01247	2.4	0.05
40	334.092	14.30885	0.141	0.00374	21.208	0.82236	2.3	0.08165	2.2	0.04
50	434.53	14.50686	0.258	0.00432	21.532	0.83236	2.6	0.08165	2.1	0.04

Treatment Time (min)	Fixed ground electrode									
	NO_3^- Concentration (mg/L)	Error (±)	NO_2^- Concentration (mg/L)	Error (±)	H_2O_2 Concentration (mg/L)	Error (±)	O_3 Concentration (mg/L)	Error (±)	pH	Error (±)
10	188.759	4.45047	55.927	1.60328	59.105	2.17695	0.05	0.00471	3	0.09
20	298.009	8.9956	81.889	1.58789	182.827	6.12566	0.1	0.00816	2.9	0.08
30	326.662	10.9974	62.479	2.04127	125.744	4.09752	0.3	0.00816	2.6	0.06
40	364.581	6.08816	65.704	2.53927	97.843	2.04764	0.2	0.00471	2.8	0.06
50	418.852	7.70105	99.143	1.19708	105.889	2.44505	0.7	0.02357	2.6	0.06
