# Supplementary material for: Nitrogen fixation efficiency enhancement using plasma jet: Impact of grounded electrode
Source: PLoS One. 2026 Sep 1;21(9):e0346219. doi: 10.1371/journal.pone.0346219 (PMC13533402; doi:10.1371/journal.pone.0346219)
Supplement: Supplementary file 6 [file pone.0346219.s006.pdf]

Data of fig 7.

Time (μS)	Fixed ground electrode		Floating ground water electrode	
	Voltage (kV)	Current (mA)	Voltage (kV)	Current (mA)
0	0.06	22.4	-0.44	22
0.1	0	10.8	-0.5	3.6
0.2	-0.02	12.4	-0.5	19.6
0.3	0.04	2.8	-0.46	-18
0.4	0.04	6.4	-0.48	-3.2
0.5	-0.04	-4.4	-0.32	-73.2
0.6	-0.02	3.2	-0.34	-73.6
0.7	-0.1	-20.4	-0.18	-147.2
0.8	-0.1	-4.4	-0.18	-131.2
0.9	0.04	-22.4	-0.26	-202.4
1	0	-24.4	-0.26	-191.2
1.1	0.08	-69.2	-0.18	-225.2
1.2	0.06	-64.4	-0.2	-210
1.3	0.22	-135.2	-0.06	-248.8
1.4	0.2	-128.8	-0.08	-240.4
1.5	0.3	-199.2	0	-266
1.6	0.3	-176.4	-0.06	-249.6
1.7	0.24	-230.4	0	-283.2
1.8	0.24	-216.4	-0.02	-254.4
1.9	0.34	-254.8	0.06	-284.4
2	0.32	-239.2	0.02	-276.4
2.1	0.44	-264.4	0.12	-254.4
2.2	0.4	-262.8	0.1	-255.2
2.3	0.46	-280.4	0.18	-286.8
2.4	0.42	-259.2	0.18	-280.4
2.5	0.5	-287.6	0.14	-257.2
2.6	0.46	-286.4	0.14	-272.4
2.7	0.54	-260.8	0.22	-264.4
2.8	0.52	-281.6	0.2	-263.2
2.9	0.6	-272.4	0.3	-255.2
3	0.56	-266.4	0.28	-272.4
3.1	0.62	-289.2	0.34	-249.2
3.2	0.6	-282.4	0.34	-263.2
3.3	0.66	-265.2	0.3	-240.8
3.4	0.62	-281.2	0.3	-259.6
3.5	0.7	-256.8	0.42	-248.4
3.6	0.66	-272.8	0.38	-248.4
3.7	0.76	-263.6	0.46	-240.4
3.8	0.7	-279.6	0.42	-256
3.9	0.78	-258.4	0.5	-226.4
4	0.74	-279.2	0.46	-242.4
4.1	0.82	-249.2	0.54	-231.2
4.2	0.78	-258	0.5	-232.4
4.3	0.86	-263.2	0.58	-242.4

4.4	0.8	-263.2	0.54	-248.4
4.5	0.9	-240.4	0.6	-226
4.6	0.86	-261.2	0.56	-246.4
4.7	0.94	-238.8	0.64	-222.4
4.8	0.9	-246.4	0.6	-236.4
4.9	0.98	-236	0.68	-223.2
5	0.94	-248.4	0.64	-245.2
5.1	1.02	-237.2	0.72	-214.4
5.2	0.96	-237.2	0.68	-237.2
5.3	1.04	-246.4	0.76	-207.2
5.4	0.98	-238.4	0.72	-210.8
5.5	1.08	-230.4	0.78	-220.4
5.6	1.02	-246.4	0.74	-204.8
5.7	1.1	-223.2	0.82	-227.6
5.8	1.06	-237.2	0.76	-227.2
5.9	1.14	-212.4	0.84	-204.4
6	1.1	-212.4	0.8	-211.2
6.1	1.18	-236.4	0.88	-201.6
6.2	1.12	-219.2	0.84	-196.4
6.3	1.18	-221.2	0.92	-218
6.4	1.14	-230.4	0.86	-202
6.5	1.22	-212.4	0.94	-197.6
6.6	1.16	-220.8	0.88	-201.6
6.7	1.26	-212.4	0.96	-195.6
6.8	1.2	-203.2	0.92	-203.2
6.9	1.28	-232.4	0.98	-189.2
7	1.24	-222.4	0.94	-204.4
7.1	1.32	-212.4	1.02	-182.4
7.2	1.26	-206.8	0.96	-200.4
7.3	1.34	-229.2	1.04	-189.6
7.4	1.28	-221.2	1	-204.4
7.5	1.38	-200.4	1.06	-182.4
7.6	1.32	-215.2	1.02	-189.2
7.7	1.4	-205.2	1.08	-199.2
7.8	1.36	-220.4	1.04	-195.2
7.9	1.44	-190.8	1.1	-172.8
8	1.38	-211.2	1.06	-196
8.1	1.46	-187.2	1.14	-172.8
8.2	1.4	-203.2	1.08	-169.6
8.3	1.48	-194.4	1.16	-197.2
8.4	1.5	-206.8	1.1	-179.2
8.5	1.42	-198.4	1.18	-188.8
8.6	1.46	-208.4	1.12	-188.4
8.7	1.54	-197.6	1.2	-171.2
8.8	1.48	-200.4	1.14	-186
8.9	1.56	-192.4	1.22	-164.4
9	1.5	-207.2	1.16	-179.2
9.1	1.58	-183.2	1.24	-169.6

9.2	1.54	-200.4	1.18	-167.2
9.3	1.6	-182.4	1.26	-176.4
9.4	1.64	-193.2	1.2	-177.2
9.5	1.54	-182.8	1.28	-159.6
9.6	1.62	-283.6	1.22	-169.2
9.7	0.22	-104.8	1.3	-160
9.8	0.22	-168.4	1.22	-154
9.9	-0.54	-216.4	1.3	-176.8
10	-0.36	-175.2	1.24	-154
10.1	-0.56	-36.8	1.32	-164.4
10.2	-0.44	-77.2	1.26	-165.2
10.3	-0.36	139.2	1.34	-156.4
10.4	-0.36	137.6	1.28	-152.4
10.5	-0.28	306.8	1.36	-175.2
10.6	-0.32	307.6	1.28	-152.4
10.7	-0.44	449.2	1.36	-176.4
10.8	-0.42	440.8	1.32	-160.8
10.9	-0.5	521.6	1.38	-160.8
11	-0.46	519.2	1.32	-166.4
11.1	-0.52	551.6	1.4	-142.8
11.2	-0.52	550.8	1.34	-143.2
11.3	-0.48	560.8	1.42	-167.2
11.4	-0.52	553.6	1.36	-166.4
11.5	-0.48	537.2	1.44	-146.4
11.6	-0.48	535.2	1.44	-55.2
11.7	-0.52	505.6	0.82	-264.8
11.8	-0.52	513.6	0.92	-125.2
11.9	-0.48	481.6	0.38	-158.8
12	-0.52	498.8	0.4	-150.4
12.1	-0.46	452.8	-0.08	-124
12.2	-0.46	466.8	-0.08	-119.2
12.3	-0.5	412.8	-0.36	-61.6
12.4	-0.5	427.6	-0.32	-70
12.5	-0.44	369.6	-0.48	41.6
12.6	-0.48	377.6	-0.44	43.2
12.7	-0.44	343.6	-0.52	158
12.8	-0.46	346.4	-0.52	164.8
12.9	-0.42	313.6	-0.58	270.8
13	-0.46	306.8	-0.58	276.8
13.1	-0.4	282.8	-0.66	363.2
13.2	-0.44	280.8	-0.64	369.6
13.3	-0.38	241.6	-0.68	451.2
13.4	-0.38	247.6	-0.64	448.8
13.5	-0.42	209.6	-0.7	499.6
13.6	-0.4	215.6	-0.66	491.6
13.7	-0.36	177.6	-0.72	522.8
13.8	-0.4	190.8	-0.7	514.8
13.9	-0.34	152.4	-0.74	522.8

14	-0.38	159.6	-0.72	522
14.1	-0.32	135.6	-0.78	489.6
14.2	-0.36	151.2	-0.76	513.6
14.3	-0.3	111.6	-0.8	467.2
14.4	-0.34	120.4	-0.76	475.6
14.5	-0.28	94.8	-0.82	443.2
14.6	-0.32	105.2	-0.78	452.8
14.7	-0.28	87.6	-0.84	417.6
14.8	-0.3	88.8	-0.8	425.6
14.9	-0.26	71.2	-0.84	388.8
15	-0.28	64.8	-0.8	402.8
15.1	-0.24	47.6	-0.84	365.6
15.2	-0.28	48.8	-0.8	374.4
15.3	-0.22	39.6	-0.84	348.8
15.4	-0.22	45.6	-0.86	350
15.5	-0.26	16.4	-0.8	325.6
15.6	-0.24	32.8	-0.8	326.4
15.7	-0.2	13.6	-0.86	299.6
15.8	-0.22	16.8	-0.84	310
15.9	-0.18	7.2	-0.76	272
16	-0.2	-0.4	-0.82	270
16.1	-0.16	7.6	-0.78	252.8
16.2	-0.18	0	-0.78	260.8
16.3	-0.14	-9.2	-0.84	222
16.4	-0.18	-7.2	-0.84	230
16.5	-0.14	-16.4	-0.78	211.6
16.6	-0.16	-1.6	-0.82	217.6
16.7	-0.12	-31.6	-0.74	188.8
16.8	-0.12	-22.8	-0.8	196.8
16.9	-0.16	-20.8	-0.74	171.2
17	-0.12	-14.4	-0.74	179.6
17.1	-0.16	-36.4	-0.8	155.6
17.2	-0.14	-32.4	-0.74	163.6
17.3	-0.08	-22.4	-0.8	145.6
17.4	-0.14	-32.4	-0.72	146.8
17.5	-0.08	-39.2	-0.78	128.8
17.6	-0.12	-39.2	-0.76	135.6
17.7	-0.08	-29.6	-0.7	105.6
17.8	-0.12	-45.2	-0.7	114.8
17.9	-0.06	-29.2	-0.76	96.8
18	-0.1	-39.2	-0.68	98
18.1	-0.06	-38.4	-0.74	89.6
18.2	-0.08	-47.2	-0.68	94.4
18.3	-0.04	-24.4	-0.72	82.8
18.4	-0.08	-46.4	-0.68	68.4
18.5	-0.04	-19.2	-0.72	90.8
18.6	-0.08	-23.2	-0.7	78.4
18.7	-0.04	-39.2	-0.66	59.6

18.8	-0.04	-36.4	-0.68	67.2
18.9	-0.06	-23.2	-0.64	43.6
19	-0.04	-38.4	-0.68	51.6
19.1	-0.08	-25.6	-0.62	41.6
19.2	-0.04	-37.2	-0.66	47.6
19.3	-0.08	-21.2	-0.62	26.8
19.4	-0.06	-44.4	-0.62	39.6
19.5	-0.02	-13.2	-0.66	16.8
19.6	-0.06	-30.4	-0.6	23.2
19.7	0	-22.4	-0.64	32.8
19.8	-0.02	-38.4	-0.62	31.6
19.9	-0.06	-13.2	-0.58	9.6
20	-0.04	-19.2	-0.6	23.6
20.1	-0.08	-11.2	-0.56	1.2
20.2	-0.08	-28.4	-0.6	8.8
20.3	-0.02	-6.4	-0.54	-0.4
20.4	-0.06	-4.8	-0.58	-6.8
20.5	0	-28.4	-0.54	7.6
20.6	-0.04	-14.4	-0.58	4.8
20.7	0	-7.2	-0.52	0.4
20.8	-0.02	-21.2	-0.56	-14.4
20.9	-0.06	2.8	-0.5	11.2
21	-0.06	-4.4	-0.54	-9.6
21.1	-0.02	2.8	-0.5	-8.4
21.2	-0.06	-13.2	-0.48	-2.4
21.3	-0.02	8.8	-0.54	-25.2
21.4	-0.04	-12.4	-0.52	-18.4
21.5	0	9.6	-0.46	-9.2
21.6	-0.04	8.8	-0.52	-25.2
21.7	0	-6	-0.46	-8.8
21.8	-0.04	-4.4	-0.48	-8.8
21.9	0	9.6	-0.42	-31.6
22	-0.04	-4.8	-0.46	-39.6
22.1	0	11.2	-0.42	-18.4
22.2	-0.04	1.6	-0.44	-39.2
22.3	0	1.2	-0.48	-17.2
22.4	-0.04	-6.4	-0.44	-34
22.5	0	17.6	-0.48	-7.2
22.6	-0.02	-3.2	-0.46	-31.2
22.7	0.02	18.8	-0.36	-8.4
22.8	-0.04	1.6	-0.4	-29.2
22.9	0.02	11.6	-0.28	1.2
23	-0.04	0.8	-0.32	-6.4
23.1	0.02	0.8	-0.22	20
23.2	-0.02	-6.4	-0.24	15.6
23.3	0.02	9.2	-0.14	58.8
23.4	-0.02	-0.4	-0.16	50.8
23.5	0.02	1.6	-0.08	113.6

23.6	0.02	-6.4	-0.1	105.6
23.7	-0.02	7.6	-0.02	161.6
23.8	0.02	-7.2	-0.06	161.6
23.9	-0.02	7.2	0	240.8
24	0.02	-2.4	0	226.8
24.1	-0.02	0.8	-0.06	299.6
24.2	0.02	-6.4	-0.02	291.6
24.3	-0.02	9.6	-0.1	339.6
24.4	-0.02	-5.2	-0.06	332.8
24.5	0.04	16.8	-0.16	370.8
24.6	0	-3.2	-0.12	353.6
24.7	0.06	19.2	-0.22	363.6
24.8	0.06	2.8	-0.18	371.2
24.9	0	12.4	-0.28	339.6
25	0.04	-2.4	-0.26	348.8
25.1	-0.02	19.6	-0.34	295.6
25.2	-0.02	6.8	-0.3	313.6
25.3	0.02	21.2	-0.4	243.6
25.4	0.02	4.8	-0.36	249.2
25.5	-0.04	27.2	-0.42	162.8
25.6	-0.02	32	-0.4	170.8
25.7	-0.08	22.4	-0.44	95.6
25.8	-0.06	22	-0.4	104.8
25.9	-0.02	30.4	-0.46	26.8
26	-0.06	28.8	-0.44	27.6
26.1	0	53.2	-0.38	-22.8
26.2	-0.04	49.6	-0.42	-27.2
26.3	0.04	83.6	-0.36	-67.2
26.4	0	75.6	-0.38	-67.2
26.5	0.06	137.6	-0.32	-114.4
26.6	0.04	130.8	-0.34	-102.8
26.7	0.1	178.8	-0.26	-126.4
26.8	0.06	177.6	-0.3	-120.4
26.9	0.1	243.2	-0.22	-109.6
27	0.12	241.6	-0.26	-112.4
27.1	0.06	297.2	-0.18	-88.4
27.2	0.1	288.8	-0.2	-95.2
27.3	0.04	343.6	-0.14	-55.2
27.4	0.08	345.6	-0.18	-56.4
27.5	0.02	370.8	-0.14	-14.4
27.6	0.06	362.8	-0.12	-17.2
27.7	-0.02	395.2	-0.18	25.2
27.8	0.02	386.8	-0.12	20.4
27.9	-0.06	369.2	-0.18	55.2
28	-0.02	372.8	-0.14	47.6
28.1	-0.1	338.8	-0.2	95.6
28.2	-0.08	340.8	-0.16	80.8
28.3	-0.14	290.8	-0.22	107.2

28.4	-0.12	292.8	-0.2	90.8
28.5	-0.18	230.4	-0.26	123.2
28.6	-0.14	233.6	-0.22	113.6
28.7	-0.2	156.4	-0.3	90.8
28.8	-0.18	161.6	-0.26	99.6
28.9	-0.16	83.6	-0.34	81.2
29	-0.18	88.8	-0.3	88.8
29.1	-0.14	26.8	-0.36	41.6
29.2	-0.14	39.6	-0.32	55.2
29.3	-0.18	-31.2	-0.38	7.6
29.4	-0.16	-31.6	-0.34	22.8
29.5	-0.1	-57.2	-0.38	-23.2
29.6	-0.12	-58.4	-0.38	-22.4
29.7	-0.04	-88	-0.32	-46.8
29.8	-0.08	-73.2	-0.36	-40.8
29.9	-0.02	-97.2	-0.32	-86.4
30	-0.04	-93.6	-0.32	-78.4
30.1	0.02	-69.6	-0.36	-94.4
30.2	-0.02	-69.6	-0.34	-77.2
30.3	0.04	-44.4	-0.28	-99.2
30.4	0	-52.8	-0.3	-101.2
30.5	0.06	-6	-0.22	-91.2
30.6	0.02	-3.6	-0.26	-100.4
30.7	0.08	28.8	-0.2	-94.4
30.8	0.04	18.4	-0.2	-102.4
30.9	0.08	69.6	-0.24	-64.4
31	0.02	67.6	-0.18	-77.6
31.1	0.08	82	-0.24	-46.4
31.2	0.02	78.8	-0.22	-53.2
31.3	0.06	109.6	-0.16	-13.2
31.4	0.04	95.2	-0.16	-23.2
31.5	-0.02	117.6	-0.22	1.6
31.6	-0.02	117.6	-0.16	-0.4
31.7	0.02	96.8	-0.2	25.6
31.8	0.02	104.8	-0.16	23.6
31.9	-0.04	86.8	-0.22	33.6
32	0	89.2	-0.18	30.8
32.1	-0.06	63.6	-0.24	40.4
32.2	-0.02	70.8	-0.2	31.6
32.3	-0.08	24.8	-0.26	55.6
32.4	-0.08	36.4	-0.22	47.2
32.5	-0.04	-8.8	-0.26	23.6
32.6	-0.04	-2.8	-0.22	32.4
32.7	-0.08	-31.2	-0.28	14.8
32.8	-0.08	-34.4	-0.24	25.2
32.9	-0.02	-49.2	-0.28	-0.4
33	-0.06	-48.4	-0.24	-7.2
33.1	-0.02	-89.6	-0.3	-16.8

33.2	-0.04	-82	-0.3	-23.2
33.3	0	-92.4	-0.24	-32.4
33.4	-0.04	-80.4	-0.3	-39.2
33.5	0.02	-89.2	-0.24	-48.4
33.6	-0.02	-95.6	-0.3	-50.4
33.7	0.04	-64.4	-0.24	-79.6
33.8	0	-71.6	-0.28	-63.2
33.9	0.06	-62.4	-0.22	-74.4
34	0.02	-60	-0.26	-71.2
34.1	0.06	-28.4	-0.2	-80.8
34.2	0.02	-41.2	-0.24	-80.4
34.3	0.06	-3.2	-0.2	-72.4
34.4	0.02	-9.2	-0.18	-72
34.5	0.08	14.4	-0.22	-57.2
34.6	0.04	6	-0.22	-63.2
34.7	0.08	37.2	-0.16	-48.4
34.8	0.02	28.8	-0.16	-41.6
34.9	0.06	46.8	-0.2	-39.6
35	0.04	38.8	-0.16	-38.8
35.1	0	62	-0.2	-4.8
35.2	0	54.8	-0.16	-14.4
35.3	0.04	55.2	-0.2	-4.4
35.4	0.04	58.8	-0.16	-8.4
35.5	-0.02	36.4	-0.2	9.6
35.6	0.02	51.2	-0.16	-8.4
35.7	-0.02	28.8	-0.2	24.8
35.8	-0.02	36	-0.16	8.8
35.9	0.02	19.6	-0.22	18.4
36	-0.04	21.2	-0.18	2.8
36.1	0.02	3.2	-0.24	26.8
36.2	-0.04	10.8	-0.2	24.8
36.3	0.02	-19.2	-0.24	1.6
36.4	0.02	-17.2	-0.2	16.8
36.5	-0.04	-25.6	-0.24	-0.4
36.6	-0.02	-25.6	-0.22	1.6
36.7	0.02	-42	-0.26	-3.2
36.8	-0.02	-48	-0.22	0.8
36.9	0.02	-25.6	-0.26	-23.2
37	-0.02	-57.2	-0.22	-12.4
37.1	0.04	-43.2	-0.26	-31.2
37.2	0	-56	-0.22	-22.4
37.3	0.04	-34	-0.26	-25.6
37.4	0	-42.8	-0.26	-45.2
37.5	0.06	-34	-0.22	-21.2
37.6	0.02	-35.2	-0.26	-44.4
37.7	0.06	-25.6	-0.2	-37.2
37.8	0.02	-34	-0.24	-38.4
37.9	0.08	-4.4	-0.2	-38.4

38	0.02	-21.2	-0.2	-46.4
38.1	0.08	-2	-0.24	-29.2
38.2	0.04	-9.2	-0.18	-35.2
38.3	0.08	16.4	-0.24	-27.2
38.4	0.04	7.6	-0.18	-36.4
38.5	0.08	16.8	-0.22	-14.4
38.6	0.08	8	-0.18	-20.8
38.7	0.02	38.8	-0.22	-12.4
38.8	0.06	24	-0.18	-14.4
38.9	0.02	26	-0.22	-7.2
39	0.02	24.8	-0.16	-5.2
39.1	0.08	32.8	-0.26	-21.2
39.2	0.08	24.4	-0.22	-4.4
39.3	0.04	14.4	-0.4	-5.2
39.4	0.06	24.8	-0.36	-13.2
39.5	0	6	-0.44	8.8
39.6	0.04	16.8	-0.44	3.6
39.7	0	-1.2	-0.4	-30.4
39.8	0	8	-0.44	-31.2
39.9	0.06	-15.6	-0.26	-94
40	0.02	-8	-0.26	-92.4
40.1	0.08	-17.6	-0.12	-166.4
40.2	0.08	-32	-0.14	-164.8
40.3	-0.02	-1.2	-0.2	-212.8
40.4	-0.02	-24	-0.2	-206.4
40.5	-0.1	-16	-0.12	-246.8
40.6	-0.08	-12.8	-0.12	-238.4
40.7	0.04	-42.8	0	-262.4
40.8	0.02	-28.4	-0.02	-259.2
40.9	0.1	-82.8	0.04	-277.2
41	0.08	-76.4	0	-270.4
41.1	0.24	-142.4	0.06	-292.4
41.2	0.22	-125.2	0.04	-279.2
41.3	0.32	-206.4	0.12	-303.2
41.4	0.3	-200.4	0.08	-294.4
41.5	0.24	-232.8	0.16	-270.8
41.6	0.24	-230	0.14	-288.4
41.7	0.34	-256.8	0.24	-278.4
41.8	0.34	-238.4	0.2	-294.4
41.9	0.46	-276.4	0.26	-272.4
42	0.42	-253.2	0.22	-287.2
42.1	0.5	-284.4	0.28	-264.8
42.2	0.44	-269.2	0.24	-258.4
42.3	0.52	-284.4	0.36	-287.2
42.4	0.48	-289.2	0.32	-278.4
42.5	0.56	-259.2	0.4	-254.4
42.6	0.52	-281.6	0.36	-277.2
42.7	0.6	-257.2	0.42	-255.2

42.8	0.56	-275.2	0.38	-251.2
42.9	0.66	-266	0.48	-260.8
43	0.62	-282	0.44	-261.2
43.1	0.68	-252.4	0.52	-251.6
43.2	0.64	-266	0.48	-258.4
43.3	0.7	-254.4	0.56	-236.4
43.4	0.66	-244.4	0.52	-257.2
43.5	0.74	-275.2	0.6	-226.8
43.6	0.72	-266	0.54	-251.2
43.7	0.78	-243.2	0.62	-228.8
43.8	0.74	-259.2	0.58	-248
43.9	0.82	-244.8	0.64	-217.6
44	0.78	-251.2	0.6	-234
44.1	0.86	-261.2	0.7	-225.6
44.2	0.82	-236.8	0.66	-219.2
44.3	0.9	-261.2	0.74	-242.8
44.4	0.86	-260.4	0.68	-230.4
44.5	0.94	-229.2	0.76	-220.4
44.6	0.9	-252.4	0.7	-236.4
44.7	0.96	-227.2	0.78	-206.4
44.8	0.92	-244.4	0.74	-205.2
44.9	1	-235.2	0.82	-229.2
45	0.96	-243.2	0.76	-222.4
45.1	1.04	-228.4	0.84	-204.8
45.2	0.98	-233.2	0.8	-222.4
45.3	1.06	-229.2	0.88	-198.8
45.4	1.02	-238.8	0.84	-215.2
45.5	1.1	-220.4	0.92	-224.4
45.6	1.06	-246.4	0.86	-214.4
45.7	1.12	-214.4	0.94	-205.2
45.8	1.08	-220.4	0.88	-221.2
45.9	1.16	-229.2	0.94	-188.8
46	1.12	-217.6	0.9	-197.2
46.1	1.18	-223.6	0.98	-206.8
46.2	1.14	-224	0.94	-195.2
46.3	1.22	-206	1.02	-205.2
46.4	1.16	-217.2	0.96	-211.2
46.5	1.26	-207.2	1.04	-181.2
46.6	1.2	-223.2	0.98	-179.2
46.7	1.28	-199.6	1.06	-201.6
46.8	1.22	-217.2	1	-182.4
46.9	1.3	-202.8	1.08	-203.6
47	1.26	-218.8	1.04	-198.4
47.1	1.32	-196.4	1.1	-180.4
47.2	1.3	-217.6	1.04	-183.2
47.3	1.36	-194.8	1.12	-197.2
47.4	1.32	-198	1.08	-192.4
47.5	1.4	-222.4	1.16	-183.2

47.6	1.34	-215.2	1.1	-183.6
47.7	1.42	-192.4	1.18	-193.2
47.8	1.36	-208.4	1.12	-194.4
47.9	1.44	-199.2	1.18	-176
48	1.46	-197.2	1.14	-185.2
48.1	1.4	-206.8	1.2	-177.2
48.2	1.42	-205.6	1.14	-189.6
48.3	1.5	-182.8	1.22	-173.6
48.4	1.44	-196.8	1.18	-181.6
48.5	1.52	-187.2	1.24	-172.4
48.6	1.46	-196.8	1.2	-188.8
48.7	1.54	-187.2	1.26	-166
48.8	1.48	-182.4	1.22	-179.6
48.9	1.56	-203.6	1.28	-171.2
49	1.5	-190.8	1.24	-173.6
49.1	1.58	-181.2	1.3	-178.4
49.2	1.54	-192.4	1.26	-172.4
49.3	1.6	-182.8	1.32	-174
49.4	1.64	-191.2	1.26	-185.2
49.5	1.54	-182.4	1.34	-162.4
49.6	1.64	-286.4	1.28	-168.8
49.7	0.38	-96.4	1.36	-178.4
49.8	0.46	-136.8	1.38	-162.4
49.9	-0.42	-233.2	1.3	-175.2
50	-0.45	-209.2	1.32	-175.2
50.1	-0.42	-104.4	1.4	-153.2
50.2	-0.42	-111.2	1.42	-70.8
50.3	-0.32	83.6	0.92	-264.4
50.4	-0.32	79.2	1.26	-105.2
50.5	-0.4	225.2	0.74	-231.2
50.6	-0.36	225.2	0.76	-171.6
50.7	-0.5	367.2	0.22	-152.8
50.8	-0.48	351.2	0.22	-162.8
50.9	-0.52	471.6	-0.24	-103.2
51	-0.46	457.2	-0.22	-122.4
51.1	-0.52	528.8	-0.42	-41.2
51.2	-0.5	519.6	-0.4	-32.4
51.3	-0.56	536.4	-0.52	62.4
51.4	-0.56	537.2	-0.48	70
51.5	-0.52	519.6	-0.58	195.6
51.6	-0.56	535.6	-0.56	183.6
51.7	-0.5	503.6	-0.66	302.8
51.8	-0.52	512	-0.62	296.4
51.9	-0.58	487.6	-0.7	407.6
52	-0.58	496.8	-0.66	400.4
52.1	-0.5	456.8	-0.72	465.6
52.2	-0.5	466.8	-0.66	460
52.3	-0.54	431.6	-0.72	515.6

52.4	-0.54	437.6	-0.7	502.8
52.5	-0.5	392.4	-0.76	524.8
52.6	-0.54	406.8	-0.74	534.8
52.7	-0.48	352.8	-0.8	502.4
52.8	-0.52	360	-0.76	518
52.9	-0.46	325.6	-0.82	493.2
53	-0.5	329.6	-0.78	500.8
53.1	-0.46	295.2	-0.84	462.8
53.2	-0.48	294.8	-0.8	470.8
53.3	-0.44	262.8	-0.86	446
53.4	-0.46	255.6	-0.88	454.8
53.5	-0.42	233.6	-0.8	415.2
53.6	-0.42	241.2	-0.82	426.8
53.7	-0.46	200.4	-0.86	388.4
53.8	-0.44	202	-0.82	403.2
53.9	-0.38	184.8	-0.88	364.8
54	-0.38	186.8	-0.88	372
54.1	-0.42	162.8	-0.82	347.6
54.2	-0.4	169.2	-0.82	349.2
54.3	-0.36	131.6	-0.88	323.2
54.4	-0.4	147.2	-0.82	330.8
54.5	-0.34	109.2	-0.86	292.8
54.6	-0.38	119.6	-0.82	302.8
54.7	-0.32	90	-0.86	278.4
54.8	-0.36	100.8	-0.82	278.4
54.9	-0.3	83.2	-0.86	254
55	-0.34	91.2	-0.84	256
55.1	-0.28	62.4	-0.8	238.4
55.2	-0.32	82.8	-0.84	230.8
55.3	-0.28	44.8	-0.8	212.8
55.4	-0.3	60.8	-0.84	216
55.5	-0.26	39.6	-0.78	190
55.6	-0.28	51.2	-0.78	197.2
55.7	-0.24	29.2	-0.82	166
55.8	-0.26	42.8	-0.82	180.8
55.9	-0.22	11.6	-0.76	150.4
56	-0.24	19.6	-0.76	158
56.1	-0.2	9.6	-0.8	139.6
56.2	-0.24	19.6	-0.8	146.8
56.3	-0.2	3.6	-0.74	118
56.4	-0.22	10.8	-0.78	126.8
56.5	-0.18	-11.2	-0.74	112.4
56.6	-0.22	9.6	-0.78	119.6
56.7	-0.16	-20.4	-0.72	88.8
56.8	-0.2	-12.4	-0.76	96
56.9	-0.16	-22.4	-0.7	86.8
57	-0.16	-22.4	-0.74	96
57.1	-0.2	-21.2	-0.68	74

57.2	-0.14	-13.2	-0.72	88.8
57.3	-0.18	-36.4	-0.68	56.8
57.4	-0.14	-30.4	-0.68	64.4
57.5	-0.18	-20.4	-0.72	54.4
57.6	-0.12	-26.8	-0.68	56.8
57.7	-0.16	-27.2	-0.72	46
57.8	-0.16	-29.2	-0.72	48.8
57.9	-0.1	-28.4	-0.64	30.8
58	-0.14	-27.2	-0.68	32
58.1	-0.1	-37.2	-0.62	40.4
58.2	-0.12	-21.2	-0.62	32
58.3	-0.08	-44.4	-0.68	22.4
58.4	-0.1	-23.2	-0.66	24
58.5	-0.06	-44.8	-0.62	14.8
58.6	-0.1	-43.6	-0.66	16
58.7	-0.04	-21.2	-0.6	16
58.8	-0.06	-23.2	-0.64	19.2
58.9	-0.1	-44.8	-0.58	-2.8
59	-0.1	-38.8	-0.6	3.6
59.1	-0.06	-30.4	-0.56	13.2
59.2	-0.1	-36.8	-0.6	3.6
59.3	-0.06	-29.2	-0.54	-6.4
59.4	-0.08	-18.4	-0.58	-5.2
59.5	-0.04	-48.4	-0.54	-6.4
59.6	-0.02	-21.2	-0.56	-8.4
59.7	-0.08	-44.4	-0.52	-16.8
59.8	-0.04	-29.2	-0.56	-14
59.9	-0.08	-21.2	-0.5	-24.8
60	-0.08	-21.6	-0.54	-6.4
60.1	-0.04	-30.8	-0.48	-28.4
60.2	-0.08	-25.2	-0.48	-21.2
60.3	-0.02	-16	-0.54	-12.4
60.4	-0.06	-27.6	-0.5	-29.2
60.5	-0.02	-17.2	-0.46	-12.4
60.6	-0.06	-32.8	-0.5	-17.2
60.7	-0.02	-9.2	-0.44	-27.2
60.8	-0.02	-21.2	-0.44	-33.6
60.9	-0.06	-6.4	-0.48	-9.2
61	-0.06	-13.2	-0.48	-27.2
61.1	-0.04	-8.4	-0.42	-18
61.2	-0.06	-24.4	-0.46	-34
61.3	-0.02	0.8	-0.38	-12.4
61.4	-0.02	1.2	-0.42	-26
61.5	-0.06	-21.2	-0.36	-30.4
61.6	-0.06	-8.8	-0.36	-28.4
61.7	0	-0.8	-0.42	-27.2
61.8	-0.06	-18	-0.38	-34
61.9	-0.02	5.6	-0.44	-11.2

62	-0.04	-16	-0.42	-11.2
62.1	-0.02	7.2	-0.36	-28.8
62.2	0	2.8	-0.4	-27.2
62.3	-0.04	-0.4	-0.3	-5.2
62.4	0	-14	-0.32	-12.8
62.5	-0.04	15.6	-0.22	2.8
62.6	-0.04	1.6	-0.24	-4.4
62.7	0	1.2	-0.14	34.8
62.8	-0.04	-5.2	-0.18	19.6
62.9	0	17.6	-0.06	84.8
63	-0.04	10.8	-0.08	75.6
63.1	0	0.8	0	132.8
63.2	-0.04	12.8	-0.04	140.8
63.3	0.02	2.8	0.04	195.6
63.4	-0.02	12	0	198.8
63.5	0.02	2.8	0.04	250.8
63.6	-0.02	1.6	0.04	257.2
63.7	0.02	10.4	-0.02	307.6
63.8	0.02	9.6	0.02	305.6
63.9	-0.04	1.2	-0.06	361.6
64	-0.02	-1.6	-0.04	347.6
64.1	0.04	7.6	-0.12	370.8
64.2	0.04	-7.2	-0.1	358.4
64.3	0	15.6	-0.22	392.4
64.4	0.02	-0.4	-0.18	376
64.5	-0.04	9.6	-0.26	338
64.6	0	-5.2	-0.26	350.8
64.7	-0.04	17.6	-0.34	288.8
64.8	-0.02	9.6	-0.32	288.8
64.9	-0.06	18.8	-0.4	232.4
65	-0.04	7.2	-0.36	230.8
65.1	-0.1	17.6	-0.42	141.2
65.2	-0.1	7.6	-0.4	149.2
65.3	-0.06	25.2	-0.44	75.6
65.4	-0.08	17.6	-0.44	78.4
65.5	-0.04	34.8	-0.4	13.2
65.6	-0.08	25.6	-0.44	10
65.7	-0.02	73.2	-0.38	-54.4
65.8	-0.04	60.8	-0.4	-47.2
65.9	0.02	115.6	-0.34	-96.4
66	0	107.6	-0.38	-88.4
66.1	0.04	156.8	-0.3	-127.2
66.2	0.06	148.4	-0.34	-117.2
66.3	0.02	218.8	-0.26	-126.8
66.4	0.02	218.8	-0.28	-133.6
66.5	0.08	267.6	-0.2	-94.8
66.6	0.06	267.6	-0.24	-108.8
66.7	0.02	324.8	-0.16	-75.2

66.8	0.06	323.2	-0.2	-76.8
66.9	0	356.8	-0.14	-43.2
67	0.02	354.8	-0.18	-38.4
67.1	-0.04	378.8	-0.12	-3.6
67.2	-0.02	362.8	-0.12	1.2
67.3	-0.08	387.6	-0.16	34.8
67.4	-0.08	381.6	-0.12	31.6
67.5	-0.14	350.8	-0.18	73.2
67.6	-0.12	356.8	-0.14	72.8
67.7	-0.18	300.8	-0.2	97.6
67.8	-0.16	314.8	-0.16	89.6
67.9	-0.22	243.6	-0.24	111.6
68	-0.18	251.6	-0.18	103.2
68.1	-0.24	171.2	-0.26	110.8
68.2	-0.2	177.6	-0.22	110.8
68.3	-0.24	112.8	-0.3	95.6
68.4	-0.2	112	-0.26	96.8
68.5	-0.24	47.2	-0.32	67.6
68.6	-0.22	47.6	-0.3	79.2
68.7	-0.18	-14.8	-0.34	33.2
68.8	-0.22	-6.4	-0.36	41.2
68.9	-0.16	-55.2	-0.3	7.2
69	-0.18	-52	-0.32	-0.8
69.1	-0.12	-69.2	-0.36	-32.4
69.2	-0.16	-66.4	-0.36	-30.8
69.3	-0.1	-85.2	-0.3	-55.2
69.4	-0.1	-83.2	-0.34	-56.4
69.5	-0.04	-60.4	-0.28	-95.6
69.6	-0.08	-68.4	-0.32	-86.8
69.7	0	-41.2	-0.26	-104.4
69.8	-0.04	-37.2	-0.3	-96.4
69.9	0.02	-13.2	-0.22	-120.4
70	-0.02	-13.2	-0.26	-104
70.1	0.02	33.6	-0.2	-112.4
70.2	0	23.6	-0.22	-103.2
70.3	0.04	48.8	-0.18	-87.2
70.4	0.04	55.6	-0.2	-93.2
70.5	0	89.6	-0.14	-56.4
70.6	0.04	77.6	-0.14	-74.4
70.7	-0.02	102.8	-0.18	-39.6
70.8	0.02	96.8	-0.16	-41.2
70.9	-0.02	119.2	-0.1	-15.2
71	0	111.6	-0.1	-24
71.1	-0.06	101.6	-0.16	13.6
71.2	-0.06	119.6	-0.12	1.6
71.3	-0.02	87.6	-0.18	39.2
71.4	-0.08	94.8	-0.14	22.8
71.5	-0.04	70.8	-0.2	46.8

71.6	-0.04	72.4	-0.16	39.6
71.7	-0.08	39.2	-0.2	57.6
71.8	-0.1	46	-0.16	57.2
71.9	-0.06	-0.4	-0.2	32.4
72	-0.1	15.6	-0.18	50
72.1	-0.06	-17.2	-0.24	32.8
72.2	-0.1	-20.8	-0.22	42.8
72.3	-0.06	-46.4	-0.26	26.8
72.4	-0.08	-56.4	-0.22	33.2
72.5	-0.04	-72	-0.26	3.6
72.6	-0.08	-64.4	-0.22	19.2
72.7	-0.04	-86.4	-0.28	-18.8
72.8	-0.06	-77.2	-0.28	-8.4
72.9	0	-86.4	-0.22	-38
73	0	-93.2	-0.22	-27.2
73.1	-0.04	-70.4	-0.26	-44.8
73.2	-0.02	-78.4	-0.26	-44.8
73.3	0.02	-61.2	-0.2	-65.6
73.4	-0.02	-69.2	-0.26	-45.2
73.5	0.04	-38.4	-0.2	-75.2
73.6	0.04	-56	-0.24	-59.2
73.7	0	-11.2	-0.18	-80.4
73.8	0.04	-20.4	-0.22	-68.8
73.9	0	3.6	-0.16	-66.8
74	0.04	2.8	-0.16	-69.2
74.1	0	19.2	-0.2	-60.4
74.2	0	8.8	-0.2	-60.4
74.3	0.04	40.4	-0.14	-30.4
74.4	0	32.8	-0.14	-44.4
74.5	0.04	41.2	-0.18	-20.4
74.6	0.02	33.2	-0.18	-29.2
74.7	-0.02	55.6	-0.14	-3.2
74.8	0	37.6	-0.14	-14.4
74.9	-0.04	48	-0.2	3.6
75	-0.04	39.6	-0.2	-4.4
75.1	0	22.4	-0.14	17.6
75.2	-0.06	39.6	-0.16	1.6
75.3	-0.02	6.4	-0.2	26.8
75.4	-0.06	13.6	-0.18	26.8
75.5	-0.02	-0.4	-0.22	3.6
75.6	-0.06	7.2	-0.18	9.6
75.7	-0.02	-23.6	-0.24	19.6
75.8	-0.06	-6.4	-0.2	13.2
75.9	-0.02	-38.8	-0.24	12.8
76	-0.06	-32.4	-0.2	18.8
76.1	-0.02	-50.4	-0.26	-12.4
76.2	-0.02	-41.2	-0.26	4.8
76.3	-0.06	-59.6	-0.2	-13.2

76.4	-0.04	-55.6	-0.22	-5.2
76.5	0	-63.6	-0.26	-28.4
76.6	0	-61.2	-0.22	-15.2
76.7	-0.04	-48.4	-0.26	-36.8
76.8	-0.04	-56.4	-0.26	-35.6
76.9	0.02	-46.8	-0.22	-37.2
77	-0.02	-49.2	-0.24	-39.2
77.1	0.04	-39.6	-0.2	-52.8
77.2	0	-48.8	-0.2	-46.8
77.3	0.04	-26	-0.24	-38.4
77.4	0	-35.6	-0.24	-44.8
77.5	0.04	-16.4	-0.18	-37.2
77.6	0	-25.2	-0.24	-42.4
77.7	0.06	4.4	-0.18	-40.4
77.8	0.04	-18.4	-0.18	-37.2
77.9	0	4.4	-0.22	-28.4
78	0.04	-3.6	-0.16	-29.2
78.1	-0.02	14.4	-0.22	-21.2
78.2	-0.02	-2.4	-0.2	-29.6
78.3	0.02	20.4	-0.16	1.2
78.4	0	20.4	-0.16	-17.2
78.5	0.04	-2.8	-0.2	-8
78.6	0.04	-1.2	-0.14	-11.6
