# Supplementary material for: Nitrogen fixation efficiency enhancement using plasma jet: Impact of grounded electrode
Source: PLoS One. 2026 Sep 1;21(9):e0346219. doi: 10.1371/journal.pone.0346219 (PMC13533402; doi:10.1371/journal.pone.0346219)
Supplement: Supplementary file 7 [file pone.0346219.s007.pdf]

Data of fig 8(a).

Gas composition ratio	Energy cost (MJ/mole)	Error (±)	Power (W)	Error (±)
N_2	199.5878	7.53343	39.75846	1.50068
Air	51.02804	2.02547	27.15949	1.07805
Air:O_2::5:3	45.28728	2.33673	34.19888	1.76459
Air:O_2::1:3	52.14755	3.9436	26.44219	1.99966
O_2	38490.04	2237.301	34.83267	2.02471

Data of fig 8(b).

Gas flow rate (L/min)	Energy cost (MJ/mole)	Error (±)	Power (W)	Error (±)
2	42.36412	2.01301	31.58204	1.50068
3	33.29297	1.79517	27.41174	1.47805
4	45.28728	2.33673	34.19888	1.76459
5	80.26401	3.47571	39.25	1.69966
6	97.88655	1.69135	59.30501	1.02471

Data of fig 8(c).

Treatment Time (min)	Floating water ground electrode		Fixed ground electrode	
	Energy cost (MJ/mole)	Error (±)	Energy cost (MJ/mole)	Error (±)
10	71.49501	2.0394	25.73688	0.9621
20	72.60109	2.07095	33.29297	1.24457
30	107.9457	3.07916	49.63661	1.85553
40	100.4752	2.86606	60.00911	2.24328
50	96.54168	2.75386	61.52365	1.64329
